# Supplementary material for: Generalization of contextual fear is sex-specifically affected by high salt intake
Source: PLoS One. 2023 Jul 13;18(7):e0286221. doi: 10.1371/journal.pone.0286221 (PMC10343085; doi:10.1371/journal.pone.0286221)
Supplement: S1 Table — (PDF) [file pone.0286221.s001.pdf]

Supplemental Material for

Generalization of contextual fear is sex-specifically affected by high salt intake

Jasmin N. Beaver[1,2], Brady L. Weber[1,2], Matthew T. Ford[1], Anna E. Anello[1,2], Kaden M. Ruffin[1], Sarah K. Kassis[1,2], T. Lee Gilman[1,2,3]*

[1]Department of Psychological Sciences, Kent State University, Kent, Ohio, United States of America

[2]Brain Health Research Institute, Kent State University, Kent, Ohio, United States of America

[3]Healthy Communities Research Institute, Kent State University, Kent, Ohio, United States of America

*Corresponding Author

Email: lgilman1@kent.edu (TLG)

S1 Table. Three-way repeated measures ANOVAs on context fear 'training' for control no shock mice across Experiments.

S1A Table

**Experiment 1**	**No Shock – Context Fear Training**		
Sex	$F(1,30)=0.036$	$p=0.850$	partial $\eta^2=0.001$
Diet	$F(1,30)=1.003$	$p=0.325$	partial $\eta^2=0.032$
Time	$F(2.92,87.68)=3.688$	$\mathbf{p=0.016}$	partial $\eta^2=\mathbf{0.109}$
Time × Sex	$F(2.92,87.68)=2.055$	$p=0.114$	partial $\eta^2=0.064$
Time × Diet	$F(2.92,87.68)=0.653$	$p=0.579$	partial $\eta^2=0.021$
Sex × Diet	$F(1,30)=0.000$	$p=0.990$	partial $\eta^2=0.000$
Time × Sex × Diet	$F(2.92,87.68)=2.404$	$p=0.074$	partial $\eta^2=0.074$

S1B Table

**Experiment 2**	**No Shock – Context Fear Training**		
Sex	$F(1,30)=0.516$	$p=0.478$	partial $\eta^2=0.017$
Diet	$F(1,30)=0.012$	$p=0.915$	partial $\eta^2=0.000$
Time	$F(3.05,91.62)=6.466$	$\mathbf{p<0.001}$	partial $\eta^2=\mathbf{0.177}$
Time × Sex	$F(3.05,91.62)=1.258$	$p=0.294$	partial $\eta^2=0.040$
Time × Diet	$F(3.05,91.62)=1.032$	$p=0.383$	partial $\eta^2=0.033$
Sex × Diet	$F(1,30)=3.135$	$p=0.087$	partial $\eta^2=0.095$
Time × Sex × Diet	$F(3.05,91.62)=0.710$	$p=0.551$	partial $\eta^2=0.023$

S1C Table

**Experiment 3**	**No Shock – Context Fear Training**		
Sex	$F(1,27)=11.10$	$p=0.003$	partial $\eta^2=0.291$
Diet	$F(1,27)=0.245$	$p=0.625$	partial $\eta^2=0.009$
Time	$F(2.44,65.88)=3.016$	$p=0.046$	partial $\eta^2=0.100$
Time × Sex	$F(2.44,65.88)=3.091$	$\mathbf{p=0.042}$	partial $\eta^2=\mathbf{0.103}$
Time × Diet	$F(2.44,65.88)=0.668$	$p=0.545$	partial $\eta^2=0.024$
Sex × Diet	$F(1,27)=2.753$	$p=0.109$	partial $\eta^2=0.093$
Time × Sex × Diet	$F(2.44,65.88)=1.590$	$p=0.207$	partial $\eta^2=0.056$
