# Supplementary material for: Generalization of contextual fear is sex-specifically affected by high salt intake
Source: PLoS One. 2023 Jul 13;18(7):e0286221. doi: 10.1371/journal.pone.0286221 (PMC10343085; doi:10.1371/journal.pone.0286221)
Supplement: S2 Table — (PDF) [file pone.0286221.s002.pdf]

Supplemental Material for

Generalization of contextual fear is sex-specifically affected by high salt intake

Jasmin N. Beaver[1,2], Brady L. Weber[1,2], Matthew T. Ford[1], Anna E. Anello[1,2], Kaden M. Ruffin[1], Sarah K. Kassis[1,2], T. Lee Gilman[1,2,3]*

[1]Department of Psychological Sciences, Kent State University, Kent, Ohio, United States of America

[2]Brain Health Research Institute, Kent State University, Kent, Ohio, United States of America

[3]Healthy Communities Research Institute, Kent State University, Kent, Ohio, United States of America

*Corresponding Author

Email: lgilman1@kent.edu (TLG)

S2 Table. Three-way ANOVAs on context fear testing of control no shock mice across Experiments.

Context Fear Expression	No Shock Groups Across Experiments		
Sex	$F(1,87)=2.852$	$p=0.095$	partial $\eta^2=0.032$
Diet	$F(1,87)=0.497$	$p=0.483$	partial $\eta^2=0.006$
Experiment	$F(2,87)=3.187$	$p=0.046$	partial $\eta^2=0.068$
Sex × Diet	$F(1,87)=0.748$	$p=0.390$	partial $\eta^2=0.009$
Sex × Experiment	$F(2,87)=6.137$	**$p=0.003$**	partial $\eta^2=$**0.124**
Diet × Experiment	$F(2,87)=0.839$	$p=0.436$	partial $\eta^2=0.019$
Sex × Diet × Experiment	$F(2,87)=0.447$	$p=0.641$	partial $\eta^2=0.010$
