# Supplementary material for: Generalization of contextual fear is sex-specifically affected by high salt intake
Source: PLoS One. 2023 Jul 13;18(7):e0286221. doi: 10.1371/journal.pone.0286221 (PMC10343085; doi:10.1371/journal.pone.0286221)
Supplement: S3 Table — (PDF) [file pone.0286221.s003.pdf]

Supplemental Material for

Generalization of contextual fear is sex-specifically affected by high salt intake

Jasmin N. Beaver[1,2], Brady L. Weber[1,2], Matthew T. Ford[1], Anna E. Anello[1,2], Kaden M. Ruffin[1], Sarah K. Kassis[1,2], T. Lee Gilman[1,2,3]*

[1]Department of Psychological Sciences, Kent State University, Kent, Ohio, United States of America

[2]Brain Health Research Institute, Kent State University, Kent, Ohio, United States of America

[3]Healthy Communities Research Institute, Kent State University, Kent, Ohio, United States of America

*Corresponding Author

Email: lgilman1@kent.edu (TLG)

S3 Table. Three-way repeated measures ANOVAs on context fear training for context fear conditioned mice of both sexes in Experiment 1.

S3A Table

**Females**	**Experiment 1 – Context Fear Training**
Diet	$F(1,29)=0.145$ $p=0.706$ partial $\eta^2=0.005$
Context	$F(1,29)=0.009$ $p=0.927$ partial $\eta^2=0.000$
Time	$F(4.00,115.9)=111.3$ $\mathbf{p<0.001}$ partial $\eta^2=\mathbf{0.793}$
Time × Diet	$F(4.00,115.9)=0.377$ $p=0.824$ partial $\eta^2=0.013$
Time × Context	$F(4.00,115.9)=0.844$ $p=0.500$ partial $\eta^2=0.028$
Diet × Context	$F(1,29)=0.024$ $p=0.879$ partial $\eta^2=0.001$
Time × Diet × Context	$F(4.00,115.9)=0.849$ $p=0.497$ partial $\eta^2=0.028$

S3B Table

**Males**	**Experiment 1 – Context Fear Training**
Diet	$F(1,28)=0.538$ $p=0.470$ partial $\eta^2=0.019$
Context	$F(1,28)=0.033$ $p=0.857$ partial $\eta^2=0.001$
Time	$F(3.56,99.68)=59.79$ $\mathbf{p<0.001}$ partial $\eta^2=\mathbf{0.681}$
Time × Diet	$F(3.56,99.68)=0.167$ $p=0.941$ partial $\eta^2=0.006$
Time × Context	$F(3.56,99.68)=0.271$ $p=0.877$ partial $\eta^2=0.010$
Diet × Context	$F(1,28)=0.013$ $p=0.909$ partial $\eta^2=0.000$
Time × Diet × Context	$F(3.56,99.68)=0.266$ $p=0.880$ partial $\eta^2=0.009$
