# Supplementary material for: Generalization of contextual fear is sex-specifically affected by high salt intake
Source: PLoS One. 2023 Jul 13;18(7):e0286221. doi: 10.1371/journal.pone.0286221 (PMC10343085; doi:10.1371/journal.pone.0286221)
Supplement: S4 Table — (PDF) [file pone.0286221.s004.pdf]

Supplemental Material for

Generalization of contextual fear is sex-specifically affected by high salt intake

Jasmin N. Beaver[1,2], Brady L. Weber[1,2], Matthew T. Ford[1], Anna E. Anello[1,2], Kaden M. Ruffin[1], Sarah K. Kassis[1,2], T. Lee Gilman[1,2,3]*

[1]Department of Psychological Sciences, Kent State University, Kent, Ohio, United States of America

[2]Brain Health Research Institute, Kent State University, Kent, Ohio, United States of America

[3]Healthy Communities Research Institute, Kent State University, Kent, Ohio, United States of America

*Corresponding Author

Email: lgilman1@kent.edu (TLG)

S4 Table. Three-way repeated measures ANOVAs on context fear training for context fear conditioned mice of both sexes in Experiment 2.

S4A Table

**Females**	**Experiment 2 – Context Fear Training**		
Diet	$F(1,29)=0.083$	$p=0.775$	partial $\eta^2=0.003$
Context	$F(1,29)=0.097$	$p=0.758$	partial $\eta^2=0.003$
Time	$F(4.11,119.1)=121.2$	**$p<0.001$**	partial $\eta^2=$**0.807**
Time × Diet	$F(4.11,119.1)=0.354$	$p=0.845$	partial $\eta^2=0.012$
Time × Context	$F(4.11,119.1)=0.259$	$p=0.908$	partial $\eta^2=0.009$
Diet × Context	$F(1,29)=0.104$	$p=0.750$	partial $\eta^2=0.004$
Time × Diet × Context	$F(4.11,119.1)=0.537$	$p=0.714$	partial $\eta^2=0.018$

S4 Table

**Males**	**Experiment 2 – Context Fear Training**		
Diet	$F(1,31)=2.080$	$p=0.159$	partial $\eta^2=0.063$
Context	$F(1,31)=0.111$	$p=0.741$	partial $\eta^2=0.004$
Time	$F(3.26,101.0)=103.1$	**$p<0.001$**	partial $\eta^2=$**0.769**
Time × Diet	$F(3.26,101.0)=2.166$	$p=0.091$	partial $\eta^2=0.065$
Time × Context	$F(3.26,101.0)=0.402$	$p=0.768$	partial $\eta^2=0.013$
Diet × Context	$F(1,31)=1.332$	$p=0.257$	partial $\eta^2=0.041$
Time × Diet × Context	$F(3.26,101.0)=1.512$	$p=0.213$	partial $\eta^2=0.047$
