# Supplementary material for: Generalization of contextual fear is sex-specifically affected by high salt intake
Source: PLoS One. 2023 Jul 13;18(7):e0286221. doi: 10.1371/journal.pone.0286221 (PMC10343085; doi:10.1371/journal.pone.0286221)
Supplement: S5 Table — (PDF) [file pone.0286221.s005.pdf]

Supplemental Material for

Generalization of contextual fear is sex-specifically affected by high salt intake

Jasmin N. Beaver[1,2], Brady L. Weber[1,2], Matthew T. Ford[1], Anna E. Anello[1,2], Kaden M. Ruffin[1], Sarah K. Kassis[1,2], T. Lee Gilman[1,2,3]*

[1]Department of Psychological Sciences, Kent State University, Kent, Ohio, United States of America

[2]Brain Health Research Institute, Kent State University, Kent, Ohio, United States of America

[3]Healthy Communities Research Institute, Kent State University, Kent, Ohio, United States of America

*Corresponding Author

Email: lgilman1@kent.edu (TLG)

S5 Table. Three-way repeated measures ANOVAs on context fear training for context fear conditioned mice of both sexes in Experiment 3.

S5A Table

**Females**	**Experiment 3 – Context Fear Training**
Diet	$F(1,30)=0.777$ $p=0.385$ partial $\eta^2=0.025$
Context	$F(1,30)=0.514$ $p=0.479$ partial $\eta^2=0.017$
Time	$F(3.82,114.7)=100.9$ $\mathbf{p<0.001}$ partial $\eta^2=\mathbf{0.771}$
Time × Diet	$F(3.82,114.7)=2.287$ $p=0.067$ partial $\eta^2=0.071$
Time × Context	$F(3.82,114.7)=1.008$ $p=0.404$ partial $\eta^2=0.033$
Diet × Context	$F(1,30)=0.250$ $p=0.621$ partial $\eta^2=0.008$
Time × Diet × Context	$F(3.82,114.7)=0.185$ $p=0.940$ partial $\eta^2=0.006$

S5B Table

**Males**	**Experiment 3 – Context Fear Training**
Diet	$F(1,27)=1.070$ $p=0.310$ partial $\eta^2=0.038$
Context	$F(1,27)=0.241$ $p=0.628$ partial $\eta^2=0.009$
Time	$F(2.84,76.71)=79.15$ $\mathbf{p<0.001}$ partial $\eta^2=\mathbf{0.746}$
Time × Diet	$F(2.84,76.71)=0.332$ $p=0.792$ partial $\eta^2=0.012$
Time × Context	$F(2.84,76.71)=0.146$ $p=0.924$ partial $\eta^2=0.005$
Diet × Context	$F(1,27)=0.533$ $p=0.472$ partial $\eta^2=0.019$
Time × Diet × Context	$F(2.84,76.71)=0.413$ $p=0.733$ partial $\eta^2=0.015$
