# Supplementary material for: Generalization of contextual fear is sex-specifically affected by high salt intake
Source: PLoS One. 2023 Jul 13;18(7):e0286221. doi: 10.1371/journal.pone.0286221 (PMC10343085; doi:10.1371/journal.pone.0286221)
Supplement: S6 Table — (PDF) [file pone.0286221.s006.pdf]

Supplemental Material for

Generalization of contextual fear is sex-specifically affected by high salt intake

Jasmin N. Beaver[1,2], Brady L. Weber[1,2], Matthew T. Ford[1], Anna E. Anello[1,2], Kaden M. Ruffin[1], Sarah K. Kassis[1,2], T. Lee Gilman[1,2,3*]

[1]Department of Psychological Sciences, Kent State University, Kent, Ohio, United States of America

[2]Brain Health Research Institute, Kent State University, Kent, Ohio, United States of America

[3]Healthy Communities Research Institute, Kent State University, Kent, Ohio, United States of America

*Corresponding Author

Email: lgilman1@kent.edu (TLG)

S6 Table. Three-way repeated measures ANOVAs on full 10 min time course of context fear testing for control no shock mice across Experiments.

**Experiment 1**	**No Shock – Context Fear Testing**		
Sex	$F(1,30)=5.414$	**$p=0.027$**	partial η^2=**0.153**
Diet	$F(1,30)=1.972$	$p=0.170$	partial $\eta^2=0.062$
Time	$F(8.43,252.8)=1.005$	$p=0.453$	partial $\eta^2=0.032$
Time × Sex	$F(8.43,252.8)=0.602$	$p=0.784$	partial $\eta^2=0.020$
Time × Diet	$F(8.43,252.8)=0.706$	$p=0.693$	partial $\eta^2=0.023$
Sex × Diet	$F(1,30)=0.138$	$p=0.713$	partial $\eta^2=0.005$
Time × Sex × Diet	$F(8.43,252.8)=1.022$	$p=0.421$	partial $\eta^2=0.033$

**Experiment 2**	**No Shock – Context Fear Testing**		
Sex	$F(1,30)=5.647$	**$p=0.024$**	partial η^2=**0.158**
Diet	$F(1,30)=0.000$	$p=0.992$	partial $\eta^2=0.000$
Time	$F(7.15,214.5)=3.614$	**$p<0.001$**	partial η^2=**0.108**
Time × Sex	$F(7.15,214.5)=1.753$	$p=0.097$	partial $\eta^2=0.055$
Time × Diet	$F(7.15,214.5)=1.166$	$p=0.323$	partial $\eta^2=0.037$
Sex × Diet	$F(1,30)=0.317$	$p=0.578$	partial $\eta^2=0.010$
Time × Sex × Diet	$F(7.15,214.5)=0.863$	$p=0.538$	partial $\eta^2=0.028$

**Experiment 3**	**No Shock – Context Fear Testing**		
Sex	$F(1,27)=3.799$	$p=0.062$	partial $\eta^2=0.123$
Diet	$F(1,27)=0.002$	$p=0.966$	partial $\eta^2=0.000$
Time	$F(7.48,202.0)=1.440$	$p=0.186$	partial $\eta^2=0.051$
Time × Sex	$F(7.48,202.0)=1.459$	$p=0.179$	partial $\eta^2=0.051$
Time × Diet	$F(7.48,202.0)=0.549$	$p=0.807$	partial $\eta^2=0.020$
Sex × Diet	$F(1,27)=0.890$	$p=0.354$	partial $\eta^2=0.032$
Time × Sex × Diet	$F(7.48,202.0)=0.459$	$p=0.874$	partial $\eta^2=0.017$
