# Supplementary material for: Generalization of contextual fear is sex-specifically affected by high salt intake
Source: PLoS One. 2023 Jul 13;18(7):e0286221. doi: 10.1371/journal.pone.0286221 (PMC10343085; doi:10.1371/journal.pone.0286221)
Supplement: S7 Table — (PDF) [file pone.0286221.s007.pdf]

Supplemental Material for

Generalization of contextual fear is sex-specifically affected by high salt intake

Jasmin N. Beaver[1,2], Brady L. Weber[1,2], Matthew T. Ford[1], Anna E. Anello[1,2], Kaden M. Ruffin[1], Sarah K. Kassis[1,2], T. Lee Gilman[1,2,3]*

[1]Department of Psychological Sciences, Kent State University, Kent, Ohio, United States of America

[2]Brain Health Research Institute, Kent State University, Kent, Ohio, United States of America

[3]Healthy Communities Research Institute, Kent State University, Kent, Ohio, United States of America

*Corresponding Author

Email: lgilman1@kent.edu (TLG)

S7 Table. Three-way ANOVAs on context fear expression (min 2-6) for context fear conditioned mice across Experiment.

	Context Trained Shock Groups		
**Context Fear Expression**	**Experiment 1**	**Experiment 2**	**Experiment 3**
Sex	$F(1,57)=7.538$<br>**$p=0.008$**<br>partial η^2=**0.117**	$F(1,60)=22.29$<br>$p<0.001$<br>partial $\eta^2=0.271$	$F(1,57)=15.19$<br>$p<0.001$<br>partial $\eta^2=0.210$
Diet	$F(1,57)=2.107$<br>$p=0.152$<br>partial $\eta^2=0.036$	$F(1,60)=6.665$<br>**$p=0.012$**<br>partial η^2=**0.100**	$F(1,57)=0.002$<br>$p=0.962$<br>partial $\eta^2=0.000$
Context	$F(1,57)=114.1$<br>**$p<0.001$**<br>partial η^2=**0.667**	$F(1,60)=171.5$<br>$p<0.001$<br>partial $\eta^2=0.741$	$F(1,57)=84.47$<br>$p<0.001$<br>partial $\eta^2=0.597$
Sex × Diet	$F(1,57)=0.191$<br>$p=0.663$<br>partial $\eta^2=0.003$	$F(1,60)=0.099$<br>$p=0.754$<br>partial $\eta^2=0.002$	$F(1,57)=5.380$<br>**$p=0.024$**<br>partial η^2=**0.086**
Sex × Context	$F(1,57)=1.297$<br>$p=0.259$<br>partial $\eta^2=0.022$	$F(1,60)=4.938$<br>**$p=0.030$**<br>partial η^2=**0.076**	$F(1,57)=9.414$<br>**$p=0.003$**<br>partial η^2=**0.142**
Diet × Context	$F(1,57)=0.486$<br>$p=0.489$<br>partial $\eta^2=0.008$	$F(1,60)=0.316$<br>$p=0.576$<br>partial $\eta^2=0.005$	$F(1,57)=0.014$<br>$p=0.905$<br>partial $\eta^2=0.000$
Sex × Diet × Context	$F(1,57)=3.235$<br>$p=0.077$<br>partial $\eta^2=0.054$	$F(1,60)=1.682$<br>$p=0.200$<br>partial $\eta^2=0.027$	$F(1,57)=2.642$<br>$p=0.110$<br>partial $\eta^2=0.044$
