# Supplementary material for: Generalization of contextual fear is sex-specifically affected by high salt intake
Source: PLoS One. 2023 Jul 13;18(7):e0286221. doi: 10.1371/journal.pone.0286221 (PMC10343085; doi:10.1371/journal.pone.0286221)
Supplement: S8 Table — (PDF) [file pone.0286221.s008.pdf]

Supplemental Material for

Generalization of contextual fear is sex-specifically affected by high salt intake

Jasmin N. Beaver[1,2], Brady L. Weber[1,2], Matthew T. Ford[1], Anna E. Anello[1,2], Kaden M. Ruffin[1], Sarah K. Kassis[1,2], T. Lee Gilman[1,2,3]*

[1]Department of Psychological Sciences, Kent State University, Kent, Ohio, United States of America

[2]Brain Health Research Institute, Kent State University, Kent, Ohio, United States of America

[3]Healthy Communities Research Institute, Kent State University, Kent, Ohio, United States of America

*Corresponding Author

Email: lgilman1@kent.edu (TLG)

S8 Table. Three-way repeated measures ANOVAs on full 10 min time course of context fear testing for mice of both sexes in Experiment 1.

S8A Table

**Females**	**Experiment 1 – Context Fear Testing**		
Diet	$F(1,29)=0.539$	$p=0.469$	partial $\eta^2=0.018$
Context	$F(1,29)=56.67$	$p<0.001$	partial $\eta^2=0.661$
Time	$F(8.41,244.0)=7.535$	$p<0.001$	partial $\eta^2=0.206$
Time × Diet	$F(8.41,244.0)=0.675$	$p=0.721$	partial $\eta^2=0.023$
Time × Context	$F(8.41,244.0)=2.142$	**$p=0.030$**	partial $\eta^2=$**0.069**
Diet × Context	$F(1,29)=0.491$	$p=0.489$	partial $\eta^2=0.017$
Time × Diet × Context	$F(8.41,244.0)=0.771$	$p=0.635$	partial $\eta^2=0.026$

S8B Table

**Males**	**Experiment 1 – Context Fear Testing**		
Diet	$F(1,28)=2.622$	$p=0.117$	partial $\eta^2=0.086$
Context	$F(1,28)=39.04$	$p=0.857$	partial $\eta^2=0.582$
Time	$F(7.48,209.4)=2.649$	$p=0.010$	partial $\eta^2=0.086$
Time × Diet	$F(7.48,209.4)=0.509$	$p=0.838$	partial $\eta^2=0.018$
Time × Context	$F(7.48,209.4)=3.909$	**$p<0.001$**	partial $\eta^2=$**0.122**
Diet × Context	$F(1,28)=3.371$	$p=0.077$	partial $\eta^2=0.107$
Time × Diet × Context	$F(7.48,209.4)=0.772$	$p=0.620$	partial $\eta^2=0.027$
