# Supplementary material for: Generalization of contextual fear is sex-specifically affected by high salt intake
Source: PLoS One. 2023 Jul 13;18(7):e0286221. doi: 10.1371/journal.pone.0286221 (PMC10343085; doi:10.1371/journal.pone.0286221)
Supplement: S9 Table — (PDF) [file pone.0286221.s009.pdf]

Supplemental Material for

Generalization of contextual fear is sex-specifically affected by high salt intake

Jasmin N. Beaver[1,2], Brady L. Weber[1,2], Matthew T. Ford[1], Anna E. Anello[1,2], Kaden M. Ruffin[1], Sarah K. Kassis[1,2], T. Lee Gilman[1,2,3]*

[1]Department of Psychological Sciences, Kent State University, Kent, Ohio, United States of America

[2]Brain Health Research Institute, Kent State University, Kent, Ohio, United States of America

[3]Healthy Communities Research Institute, Kent State University, Kent, Ohio, United States of America

*Corresponding Author

Email: lgilman1@kent.edu (TLG)

S9 Table. Three-way repeated measures ANOVAs on full 10 min time course of context fear testing for mice of both sexes in Experiment 2.

S9A Table

**Females**	**Experiment 2 – Context Fear Testing**		
Diet	$F(1,29)=0.858$	$p=0.362$	partial $\eta^2=0.029$
Context	$F(1,29)=109.8$	**$p<0.001$**	partial $\eta^2=$**0.791**
Time	$F(8.06,233.7)=4.180$	**$p<0.001$**	partial $\eta^2=$**0.126**
Time × Diet	$F(8.06,233.7)=0.873$	$p=0.541$	partial $\eta^2=0.029$
Time × Context	$F(8.06,233.7)=1.429$	$p=0.184$	partial $\eta^2=0.047$
Diet × Context	$F(1,29)=0.363$	$p=0.552$	partial $\eta^2=0.012$
Time × Diet × Context	$F(8.06,233.7)=0.739$	$p=0.658$	partial $\eta^2=0.025$

S9B Table

**Males**	**Experiment 2 – Context Fear Testing**		
Diet	$F(1,31)=3.042$	$p=0.091$	partial $\eta^2=0.089$
Context	$F(1,31)=52.22$	$p<0.001$	partial $\eta^2=0.628$
Time	$F(6.86,212.6)=6.425$	$p<0.001$	partial $\eta^2=0.172$
Time × Diet	$F(6.86,212.6)=1.542$	$p=0.066$	partial $\eta^2=0.047$
Time × Context	$F(6.86,212.6)=3.902$	**$p<0.001$**	partial $\eta^2=$**0.112**
Diet × Context	$F(1,31)=1.344$	$p=0.255$	partial $\eta^2=0.042$
Time × Diet × Context	$F(6.86,212.6)=1.188$	$p=0.312$	partial $\eta^2=0.037$
