# Supplementary material for: Generalization of contextual fear is sex-specifically affected by high salt intake
Source: PLoS One. 2023 Jul 13;18(7):e0286221. doi: 10.1371/journal.pone.0286221 (PMC10343085; doi:10.1371/journal.pone.0286221)
Supplement: S10 Table — (PDF) [file pone.0286221.s010.pdf]

Supplemental Material for

Generalization of contextual fear is sex-specifically affected by high salt intake

Jasmin N. Beaver[1,2], Brady L. Weber[1,2], Matthew T. Ford[1], Anna E. Anello[1,2], Kaden M. Ruffin[1], Sarah K. Kassis[1,2], T. Lee Gilman[1,2,3]*

[1]Department of Psychological Sciences, Kent State University, Kent, Ohio, United States of America

[2]Brain Health Research Institute, Kent State University, Kent, Ohio, United States of America

[3]Healthy Communities Research Institute, Kent State University, Kent, Ohio, United States of America

*Corresponding Author

Email: lgilman1@kent.edu (TLG)

S10 Table. Three-way repeated measures ANOVAs on full 10 min time course of context fear testing for mice of both sexes in Experiment 3.

S10A Table

Females	Experiment 3 – Context Fear Testing
Diet	$F(1,30)=0.646$ $p=0.428$ partial $\eta^2=0.021$
Context	$F(1,30)=15.64$ **$p<0.001$** partial $\eta^2=$**0.343**
Time	$F(6.57,197.0)=5.706$ **$p<0.001$** partial $\eta^2=$**0.160**
Time × Diet	$F(6.57,197.0)=0.862$ $p=0.532$ partial $\eta^2=0.028$
Time × Context	$F(6.57,197.0)=1.029$ $p=0.410$ partial $\eta^2=0.033$
Diet × Context	$F(1,30)=1.045$ $p=0.315$ partial $\eta^2=0.034$
Time × Diet × Context	$F(6.57,197.0)=0.614$ $p=0.733$ partial $\eta^2=0.020$

S10B Table

Males	Experiment 3 – Context Fear Testing
Diet	$F(1,27)=6.657$ **$p=0.016$** partial $\eta^2=$**0.198**
Context	$F(1,27)=109.0$ **$p<0.001$** partial $\eta^2=$**0.801**
Time	$F(6.91,186.7)=2.821$ **$p=0.008$** partial $\eta^2=$**0.095**
Time × Diet	$F(6.91,186.7)=0.980$ $p=0.447$ partial $\eta^2=0.035$
Time × Context	$F(6.91,186.7)=1.150$ $p=0.334$ partial $\eta^2=0.041$
Diet × Context	$F(1,27)=0.476$ $p=0.496$ partial $\eta^2=0.017$
Time × Diet × Context	$F(6.91,186.7)=1.474$ $p=0.180$ partial $\eta^2=0.052$
