# Supplementary material for: Generalization of contextual fear is sex-specifically affected by high salt intake
Source: PLoS One. 2023 Jul 13;18(7):e0286221. doi: 10.1371/journal.pone.0286221 (PMC10343085; doi:10.1371/journal.pone.0286221)
Supplement: S11 Table — (PDF) [file pone.0286221.s011.pdf]

Supplemental Material for

Generalization of contextual fear is sex-specifically affected by high salt intake

Jasmin N. Beaver[1,2], Brady L. Weber[1,2], Matthew T. Ford[1], Anna E. Anello[1,2], Kaden M. Ruffin[1], Sarah K. Kassis[1,2], T. Lee Gilman[1,2,3]*

[1]Department of Psychological Sciences, Kent State University, Kent, Ohio, United States of America

[2]Brain Health Research Institute, Kent State University, Kent, Ohio, United States of America

[3]Healthy Communities Research Institute, Kent State University, Kent, Ohio, United States of America

*Corresponding Author

Email: lgilman1@kent.edu (TLG)

S11 Table. Three-way ANOVAs on serum osmolality in control no shock mice across Experiments.

Osmolality	No Shock Groups Across Experiments		
Sex	$F(1,89)=0.424$	$p=0.517$	partial $\eta^2=0.005$
Diet	$F(1,89)=0.046$	$p=0.830$	partial $\eta^2=0.001$
Experiment	$F(1,89)=3.639$	$p=0.030$	partial $\eta^2=0.076$
Sex × Diet	$F(1,89)=0.510$	$p=0.477$	partial $\eta^2=0.006$
Sex × Experiment	$F(1,89)=4.287$	**$p=0.017$**	partial $\eta^2=$**0.088**
Diet × Experiment	$F(1,89)=1.729$	$p=0.183$	partial $\eta^2=0.037$
Sex × Diet × Experiment	$F(1,89)=1.331$	$p=0.270$	partial $\eta^2=0.029$
