# Supplementary material for: Generalization of contextual fear is sex-specifically affected by high salt intake
Source: PLoS One. 2023 Jul 13;18(7):e0286221. doi: 10.1371/journal.pone.0286221 (PMC10343085; doi:10.1371/journal.pone.0286221)
Supplement: S12 Table — (PDF) [file pone.0286221.s012.pdf]

Supplemental Material for

Generalization of contextual fear is sex-specifically affected by high salt intake

Jasmin N. Beaver[1,2], Brady L. Weber[1,2], Matthew T. Ford[1], Anna E. Anello[1,2], Kaden M. Ruffin[1], Sarah K. Kassis[1,2], T. Lee Gilman[1,2,3]*

[1]Department of Psychological Sciences, Kent State University, Kent, Ohio, United States of America

[2]Brain Health Research Institute, Kent State University, Kent, Ohio, United States of America

[3]Healthy Communities Research Institute, Kent State University, Kent, Ohio, United States of America

*Corresponding Author

Email: lgilman1@kent.edu (TLG)

S12 Table. Three-way ANOVAs on serum osmolality in context fear conditioned mice across Experiments.

	Context Trained Shock Groups		
**Osmolality**	**Experiment 1**	**Experiment 2**	**Experiment 3**
Sex	$F(1,59)=1.662$<br>$p=0.202$<br>partial $\eta^2=0.027$	$F(1,62)=0.096$<br>$p=0.758$<br>partial $\eta^2=0.002$	$F(1,55)=0.086$<br>$p=0.771$<br>partial $\eta^2=0.002$
Diet	$F(1,59)=001$<br>$p=0.979$<br>partial $\eta^2=0.000$	$F(1,62)=0.673$<br>$p=0.415$<br>partial $\eta^2=0.011$	$F(1,55)=0.745$<br>$p=0.392$<br>partial $\eta^2=0.013$
Context	$F(1,59)=1.339$<br>$p=0.252$<br>partial $\eta^2=0.022$	$F(1,62)=0.005$<br>$p=0.941$<br>partial $\eta^2=0.000$	$F(1,55)=1.440$<br>$p=0.235$<br>partial $\eta^2=0.026$
Sex × Diet	$F(1,59)=0.012$<br>$p=0.912$<br>partial $\eta^2=0.000$	$F(1,62)=0.152$<br>$p=0.698$<br>partial $\eta^2=0.002$	$F(1,55)=0.213$<br>$p=0.646$<br>partial $\eta^2=0.004$
Sex × Context	$F(1,59)=0.482$<br>$p=0.490$<br>partial $\eta^2=0.008$	$F(1,62)=0.020$<br>$p=0.888$<br>partial $\eta^2=0.000$	$F(1,55)=0.154$<br>$p=0.696$<br>partial $\eta^2=0.003$
Diet × Context	$F(1,59)=1.903$<br>$p=0.173$<br>partial $\eta^2=0.031$	$F(1,62)=0.055$<br>$p=0.815$<br>partial $\eta^2=0.001$	$F(1,55)=0.781$<br>$p=0.381$<br>partial $\eta^2=0.014$
Sex × Diet × Context	$F(1,59)=0.003$<br>$p=0.959$<br>partial $\eta^2=0.000$	$F(1,62)=1.901$<br>$p=0.173$<br>partial $\eta^2=0.030$	$F(1,55)=0.856$<br>$p=0.359$<br>partial $\eta^2=0.015$
