# Supplementary material for: Generalization of contextual fear is sex-specifically affected by high salt intake
Source: PLoS One. 2023 Jul 13;18(7):e0286221. doi: 10.1371/journal.pone.0286221 (PMC10343085; doi:10.1371/journal.pone.0286221)
Supplement: S13 Table — (PDF) [file pone.0286221.s013.pdf]

Supplemental Material for

Generalization of contextual fear is sex-specifically affected by high salt intake

Jasmin N. Beaver[1,2], Brady L. Weber[1,2], Matthew T. Ford[1], Anna E. Anello[1,2], Kaden M. Ruffin[1], Sarah K. Kassis[1,2], T. Lee Gilman[1,2,3]*

[1]Department of Psychological Sciences, Kent State University, Kent, Ohio, United States of America

[2]Brain Health Research Institute, Kent State University, Kent, Ohio, United States of America

[3]Healthy Communities Research Institute, Kent State University, Kent, Ohio, United States of America

*Corresponding Author

Email: lgilman1@kent.edu (TLG)

S13. Three-way ANOVAs on log-transformed serum corticosterone levels in control no shock mice across Experiments.

Corticosterone	No Shock Groups Across Experiments		
Sex	$F(1,81)=12.77$	$p<0.001$	partial $\eta^2=0.136$
Diet	$F(1,81)=2.498$	$p=0.118$	partial $\eta^2=0.030$
Experiment	$F(2,81)=12.81$	$p<0.001$	partial $\eta^2=0.240$
Sex × Diet	$F(1,81)=1.654$	$p=0.202$	partial $\eta^2=0.020$
Sex × Experiment	$F(2,81)=3.498$	**$p=0.035$**	partial $\eta^2=$**0.080**
Diet × Experiment	$F(2,81)=0.527$	$p=0.592$	partial $\eta^2=0.013$
Sex × Diet × Experiment	$F(2,81)=1.512$	$p=0.227$	partial $\eta^2=0.036$
