# Supplementary material for: Generalization of contextual fear is sex-specifically affected by high salt intake
Source: PLoS One. 2023 Jul 13;18(7):e0286221. doi: 10.1371/journal.pone.0286221 (PMC10343085; doi:10.1371/journal.pone.0286221)
Supplement: S14 Table — (PDF) [file pone.0286221.s014.pdf]

Supplemental Material for

Generalization of contextual fear is sex-specifically affected by high salt intake

Jasmin N. Beaver[1,2], Brady L. Weber[1,2], Matthew T. Ford[1], Anna E. Anello[1,2], Kaden M. Ruffin[1], Sarah K. Kassis[1,2], T. Lee Gilman[1,2,3]*

[1]Department of Psychological Sciences, Kent State University, Kent, Ohio, United States of America

[2]Brain Health Research Institute, Kent State University, Kent, Ohio, United States of America

[3]Healthy Communities Research Institute, Kent State University, Kent, Ohio, United States of America

*Corresponding Author

Email: lgilman1@kent.edu (TLG)

S14 Table. Three-way ANOVAs on log-transformed serum corticosterone in context fear conditioned mice across Experiments.

	Context Trained Shock Groups		
**Corticosterone**	**Experiment 1**	**Experiment 2**	**Experiment 3**
Sex	$F(1,52)=5.924$<br>**p=0.018**<br>partial η^2=**0.102**	$F(1,59)=0.973$<br>p=0.328<br>partial $\eta^2=0.016$	$F(1,53)=38.87$<br>**p<0.001**<br>partial η^2=**0.423**
Diet	$F(1,52)=1.107$<br>p=0.298<br>partial $\eta^2=0.021$	$F(1,59)=1.540$<br>p=0.220<br>partial $\eta^2=0.025$	$F(1,53)=1.170$<br>p=0.284<br>partial $\eta^2=0.022$
Context	$F(1,52)=0.325$<br>p=0.571<br>partial $\eta^2=0.006$	$F(1,59)=5.074$<br>**p=0.028**<br>partial η^2=**0.079**	$F(1,53)=0.000$<br>p=0.992<br>partial $\eta^2=0.000$
Sex × Diet	$F(1,52)=2.815$<br>p=0.099<br>partial $\eta^2=0.051$	$F(1,59)=1.041$<br>p=0.312<br>partial $\eta^2=0.017$	$F(1,53)=0.186$<br>p=0.668<br>partial $\eta^2=0.003$
Sex × Context	$F(1,52)=2.355$<br>p=0.131<br>partial $\eta^2=0.043$	$F(1,59)=19.69$<br>p=0.166<br>partial $\eta^2=0.032$	$F(1,53)=0.012$<br>p=0.913<br>partial $\eta^2=0.000$
Diet × Context	$F(1,52)=0.033$<br>p=0.856<br>partial $\eta^2=0.001$	$F(1,59)=0.056$<br>p=0.814<br>partial $\eta^2=0.001$	$F(1,53)=0.966$<br>p=0.330<br>partial $\eta^2=0.018$
Sex × Diet × Context	$F(1,52)=0.164$<br>p=0.687<br>partial $\eta^2=0.003$	$F(1,59)=0.155$<br>p=0.695<br>partial $\eta^2=0.003$	$F(1,53)=0.204$<br>p=0.654<br>partial $\eta^2=0.004$
