# Supplementary material for: Generalization of contextual fear is sex-specifically affected by high salt intake
Source: PLoS One. 2023 Jul 13;18(7):e0286221. doi: 10.1371/journal.pone.0286221 (PMC10343085; doi:10.1371/journal.pone.0286221)
Supplement: S15 Table — (PDF) [file pone.0286221.s015.pdf]

Supplemental Material for

Generalization of contextual fear is sex-specifically affected by high salt intake

Jasmin N. Beaver[1,2], Brady L. Weber[1,2], Matthew T. Ford[1], Anna E. Anello[1,2], Kaden M. Ruffin[1], Sarah K. Kassis[1,2], T. Lee Gilman[1,2,3]*

[1]Department of Psychological Sciences, Kent State University, Kent, Ohio, United States of America

[2]Brain Health Research Institute, Kent State University, Kent, Ohio, United States of America

[3]Healthy Communities Research Institute, Kent State University, Kent, Ohio, United States of America

*Corresponding Author

Email: lgilman1@kent.edu (TLG)

S15 Table. Three-way repeated measures ANOVAs on weekly average water consumption per day for context fear conditioned mice across Experiments.

S15A Table

Females	Experiment 1 – Water/day		
Diet	$F(1,30)=50.89$	**$p<0.001$**	partial η^2=**0.629**
Context	$F(1,30)=0.361$	$p=0.553$	partial $\eta^2=0.012$
Time	$F(1.64,49.08)=0.680$	$p=0.483$	partial $\eta^2=0.022$
Time × Diet	$F(1.64,49.08)=0.225$	$p=0.755$	partial $\eta^2=0.007$
Time × Context	$F(1.64,49.08)=0.011$	$p=0.977$	partial $\eta^2=0.000$
Diet × Context	$F(1,30)=0.000$	$p=0.985$	partial $\eta^2=0.000$
Time × Diet × Context	$F(1.64,49.08)=0.613$	$p=0.514$	partial $\eta^2=0.020$

S15B Table

Males	Experiment 1 – Water/day		
Diet	$F(1,29)=13.40$	**$p<0.001$**	partial η^2=**0.316**
Context	$F(1,29)=1.042$	$p=0.316$	partial $\eta^2=0.035$
Time	$F(1.27,36.92)=9.485$	**$p=0.002$**	partial η^2=**0.246**
Time × Diet	$F(1.27,36.92)=2.030$	$p=0.159$	partial $\eta^2=0.065$
Time × Context	$F(1.27,36.92)=0.411$	$p=0.574$	partial $\eta^2=0.014$
Diet × Context	$F(1,29)=1.036$	$p=0.317$	partial $\eta^2=0.035$
Time × Diet × Context	$F(1.27,36.92)=0.595$	$p=0.484$	partial $\eta^2=0.020$

S15C Table

Females	Experiment 2 – Water/day		
Diet	$F(1,30)=312.2$	$p<0.001$	partial $\eta^2=0.912$
Context	$F(1,30)=0.026$	$p=0.874$	partial $\eta^2=0.001$
Time	$F(4.11,123.3)=4.981$	$p<0.001$	partial $\eta^2=0.142$
Time × Diet	$F(4.11,123.3)=5.091$	**$p<0.001$**	partial η^2=**0.145**
Time × Context	$F(4.11,123.3)=1.342$	$p=0.258$	partial $\eta^2=0.043$
Diet × Context	$F(1,30)=3.278$	$p=0.080$	partial $\eta^2=0.099$
Time × Diet × Context	$F(4.11,123.3)=0.991$	$p=0.417$	partial $\eta^2=0.032$

S15D Table

Males	Experiment 2 – Water/day		
Diet	$F(1,32)=41.90$	$p<0.001$	partial $\eta^2=0.567$
Context	$F(1,32)=0.533$	$p=0.471$	partial $\eta^2=0.016$
Time	$F(3.25,103.9)=17.41$	$p<0.001$	partial $\eta^2=0.352$

Time × Diet	F(3.25,103.9)=3.360	**p=0.019**	partial η^2=**0.095**
Time × Context	F(3.25,103.9)=0.454	p=0.730	partial η^2=0.014
Diet × Context	F(1,32)=0.765	p=0.388	partial η^2=0.023
Time × Diet × Context	F(3.25,103.9)=0.938	p=0.431	partial η^2=0.028

S15E Table

**Females**	**Experiment 3 – Water/day**		
Diet	F(1,30)=91.88	**p<0.001**	partial η^2=**0.754**
Context	F(1,30)=0.009	p=0.927	partial η^2=0.000
Time	F(3.16,94.64)=13.26	**p<0.001**	partial η^2=**0.306**
Time × Diet	F(3.16,94.64)=1.647	p=0.182	partial η^2=0.052
Time × Context	F(3.16,94.64)=0.249	p=0.871	partial η^2=0.008
Diet × Context	F(1,30)=0.039	p=0.845	partial η^2=0.001
Time × Diet × Context	F(3.16,94.64)=0.163	p=0.928	partial η^2=0.005

S15F Table

**Males**	**Experiment 3 – Water/day**		
Diet	F(1,27)=45.38	**p<0.001**	partial η^2=**0.627**
Context	F(1,27)=0.201	p=0.657	partial η^2=0.007
Time	F(3.73,100.8)=17.83	**p<0.001**	partial η^2=**0.398**
Time × Diet	F(3.73,100.8)=2.515	p=0.050	partial η^2=0.085
Time × Context	F(3.73,100.8)=0.952	p=0.433	partial η^2=0.034
Diet × Context	F(1,27)=0.035	p=0.853	partial η^2=0.001
Time × Diet × Context	F(3.73,100.8)=0.445	p=0.763	partial η^2=0.016
