# Supplementary material for: Generalization of contextual fear is sex-specifically affected by high salt intake
Source: PLoS One. 2023 Jul 13;18(7):e0286221. doi: 10.1371/journal.pone.0286221 (PMC10343085; doi:10.1371/journal.pone.0286221)
Supplement: S16 Table — (PDF) [file pone.0286221.s016.pdf]

Supplemental Material for

Generalization of contextual fear is sex-specifically affected by high salt intake

Jasmin N. Beaver[1,2], Brady L. Weber[1,2], Matthew T. Ford[1], Anna E. Anello[1,2], Kaden M. Ruffin[1], Sarah K. Kassis[1,2], T. Lee Gilman[1,2,3]*

[1]Department of Psychological Sciences, Kent State University, Kent, Ohio, United States of America

[2]Brain Health Research Institute, Kent State University, Kent, Ohio, United States of America

[3]Healthy Communities Research Institute, Kent State University, Kent, Ohio, United States of America

*Corresponding Author

Email: lgilman1@kent.edu (TLG)

S16 Table. Three-way repeated measures ANOVAs on weekly average food consumption per day for context fear conditioned mice across Experiments.

S16A Table

**Females**	**Experiment 1 – Food/day**		
Diet	$F(1,30)=6.785$	$\mathbf{p=0.014}$	partial η^2=**0.184**
Context	$F(1,30)=0.099$	$p=0.756$	partial $\eta^2=0.003$
Time	$F(1.67,50.14)=0.650$	$p=0.500$	partial $\eta^2=0.021$
Time × Diet	$F(1.67,50.14)=0.578$	$p=0.535$	partial $\eta^2=0.019$
Time × Context	$F(1.67,50.14)=1.233$	$p=0.295$	partial $\eta^2=0.039$
Diet × Context	$F(1,30)=0.237$	$p=0.630$	partial $\eta^2=0.008$
Time × Diet × Context	$F(1.67,50.14)=0.809$	$p=0.431$	partial $\eta^2=0.026$

S16B Table

**Males**	**Experiment 1 – Food/day**		
Diet	$F(1,29)=1.427$	$p=0.242$	partial $\eta^2=0.047$
Context	$F(1,29)=0.013$	$p=0.910$	partial $\eta^2=0.000$
Time	$F(1.85,53.57)=1.825$	$p=0.174$	partial $\eta^2=0.059$
Time × Diet	$F(1.85,53.57)=0.332$	$p=0.702$	partial $\eta^2=0.011$
Time × Context	$F(1.85,53.57)=0.142$	$p=0.852$	partial $\eta^2=0.005$
Diet × Context	$F(1,29)=0.003$	$p=0.958$	partial $\eta^2=0.000$
Time × Diet × Context	$F(1.85,53.57)=0.866$	$p=0.419$	partial $\eta^2=0.029$

S16C Table

**Females**	**Experiment 2 – Food/day**		
Diet	$F(1,30)=41.57$	$\mathbf{p<0.001}$	partial η^2=**0.581**
Context	$F(1,30)=0.015$	$p=0.904$	partial $\eta^2=0.000$
Time	$F(3.72,111.5)=13.02$	$\mathbf{p<0.001}$	partial η^2=**0.303**
Time × Diet	$F(3.72,111.5)=0.188$	$p=0.936$	partial $\eta^2=0.006$
Time × Context	$F(3.72,111.5)=1.323$	$p=0.267$	partial $\eta^2=0.042$
Diet × Context	$F(1,30)=0.032$	$p=0.859$	partial $\eta^2=0.001$
Time × Diet × Context	$F(3.72,111.5)=0.433$	$p=0.771$	partial $\eta^2=0.014$

S16D Table

**Males**	**Experiment 2 – Food/day**		
Diet	$F(1,32)=3.836$	$p=0.059$	partial $\eta^2=0.107$
Context	$F(1,32)=0.128$	$p=0.722$	partial $\eta^2=0.004$
Time	$F(4.32,138.3)=10.30$	$\mathbf{p<0.001}$	partial η^2=**0.243**
Time × Diet	$F(4.32,138.3)=1.317$	$p=0.265$	partial $\eta^2=0.040$
Time × Context	$F(4.32,138.3)=0.451$	$p=0.786$	partial $\eta^2=0.014$

Diet × Context	$F(1,32)=0.672$ $p=0.418$ partial $\eta^2=0.021$
Time × Diet × Context	$F(4.32,138.3)=0.773$ $p=0.554$ partial $\eta^2=0.024$

S16E Table

**Females**	**Experiment 3 – Food/day**
Diet	$F(1,30)=16.65$ **$p<0.001$** partial $\eta^2=$**0.357**
Context	$F(1,30)=1.131$ $p=0.296$ partial $\eta^2=0.036$
Time	$F(2.78,83.39)=5.015$ **$p=0.004$** partial $\eta^2=$**0.143**
Time × Diet	$F(2.78,83.39)=1.584$ $p=0.202$ partial $\eta^2=0.050$
Time × Context	$F(2.78,83.39)=0.457$ $p=0.699$ partial $\eta^2=0.015$
Diet × Context	$F(1,30)=0.022$ $p=0.884$ partial $\eta^2=0.001$
Time × Diet × Context	$F(2.78,83.39)=0.436$ $p=0.713$ partial $\eta^2=0.014$

S16F Table

**Males**	**Experiment 3 – Food/day**
Diet	$F(1,28)=7.673$ **$p=0.010$** partial $\eta^2=$**0.215**
Context	$F(1,28)=0.017$ $p=0.897$ partial $\eta^2=0.001$
Time	$F(3.40,95.20)=5.087$ **$p=0.002$** partial $\eta^2=$**0.154**
Time × Diet	$F(3.40,95.20)=0.981$ $p=0.412$ partial $\eta^2=0.034$
Time × Context	$F(3.40,95.20)=1.256$ $p=0.294$ partial $\eta^2=0.043$
Diet × Context	$F(1,28)=0.482$ $p=0.482$ partial $\eta^2=0.017$
Time × Diet × Context	$F(3.40,95.20)=0.546$ $p=0.674$ partial $\eta^2=0.019$
