# Supplementary material for: Generalization of contextual fear is sex-specifically affected by high salt intake
Source: PLoS One. 2023 Jul 13;18(7):e0286221. doi: 10.1371/journal.pone.0286221 (PMC10343085; doi:10.1371/journal.pone.0286221)
Supplement: S17 Table — (PDF) [file pone.0286221.s017.pdf]

Supplemental Material for

Generalization of contextual fear is sex-specifically affected by high salt intake

Jasmin N. Beaver[1,2], Brady L. Weber[1,2], Matthew T. Ford[1], Anna E. Anello[1,2], Kaden M. Ruffin[1], Sarah K. Kassis[1,2], T. Lee Gilman[1,2,3]*

[1]Department of Psychological Sciences, Kent State University, Kent, Ohio, United States of America

[2]Brain Health Research Institute, Kent State University, Kent, Ohio, United States of America

[3]Healthy Communities Research Institute, Kent State University, Kent, Ohio, United States of America

*Corresponding Author

Email: lgilman1@kent.edu (TLG)

S17 Table. Three-way repeated measures ANOVAs on weekly body weight change for context fear conditioned mice across Experiments.

S17A Table

Females	Experiment 1 – Body Weight Change		
Diet	$F(1,30)=1.586$	$p=0.218$	partial $\eta^2=0.050$
Context	$F(1,30)=0.731$	$p=0.399$	partial $\eta^2=0.024$
Time	$F(1.73,51.83)=82.14$	$p<0.001$	partial $\eta^2=0.732$
Time × Diet	$F(1.73,51.83)=5.233$	**$p=0.011$**	partial $\eta^2=$**0.149**
Time × Context	$F(1.73,51.83)=3.272$	$p=0.053$	partial $\eta^2=0.098$
Diet × Context	$F(1,30)=0.675$	$p=0.418$	partial $\eta^2=0.022$
Time × Diet × Context	$F(1.73,51.83)=0.437$	$p=0.619$	partial $\eta^2=0.014$

S17B Table

Males	Experiment 1 – Body Weight Change		
Diet	$F(1,29)=0.281$	$p=0.600$	partial $\eta^2=0.010$
Context	$F(1,29)=0.998$	$p=0.326$	partial $\eta^2=0.033$
Time	$F(1.86,53.91)=30.34$	$p<0.001$	partial $\eta^2=0.511$
Time × Diet	$F(1.86,53.91)=0.330$	$p=0.705$	partial $\eta^2=0.011$
Time × Context	$F(1.86,53.91)=0.308$	$p=0.720$	partial $\eta^2=0.011$
Diet × Context	$F(1,29)=0.294$	$p=0.592$	partial $\eta^2=0.010$
Time × Diet × Context	$F(1.86,53.91)=3.318$	**$p=0.047$**	partial $\eta^2=$**0.103**

S17C Table

Females	Experiment 2 – Body Weight Change		
Diet	$F(1,30)=6.764$	**$p=0.014$**	partial $\eta^2=$**0.184**
Context	$F(1,30)=1.677$	$p=0.205$	partial $\eta^2=0.053$
Time	$F(4.06,121.8)=60.14$	**$p<0.001$**	partial $\eta^2=$**0.667**
Time × Diet	$F(4.06,121.8)=1.821$	$p=0.128$	partial $\eta^2=0.057$
Time × Context	$F(4.06,121.8)=0.594$	$p=0.670$	partial $\eta^2=0.019$
Diet × Context	$F(1,30)=0.216$	$p=0.646$	partial $\eta^2=0.007$
Time × Diet × Context	$F(4.06,121.8)=0.017$	$p=0.999$	partial $\eta^2=0.001$

S17D Table

Males	Experiment 2 – Body Weight Change		
Diet	$F(1,32)=2.211$	$p=0.147$	partial $\eta^2=0.065$
Context	$F(1,32)=0.806$	$p=0.376$	partial $\eta^2=0.025$
Time	$F(4.63,148.3)=7.269$	**$p<0.001$**	partial $\eta^2=$**0.185**
Time × Diet	$F(4.63,148.3)=2.292$	$p=0.053$	partial $\eta^2=0.067$
Time × Context	$F(4.63,148.3)=0.580$	$p=0.702$	partial $\eta^2=0.018$

Diet × Context	F(1,32)=0.257 p=0.616 partial η^2=0.008
Time × Diet × Context	F(4.63,148.3)=1.694 p=0.145 partial η^2=0.050

S17E Table

**Females**	**Experiment 3 – Body Weight Change**
Diet	F(1,30)=1.695 p=0.203 partial η^2=0.053
Context	F(1,30)=0.127 p=0.724 partial η^2=0.004
Time	F(4.26,127.8)=74.03 p<0.001 partial η^2=0.712
Time × Diet	F(4.26,127.8)=3.422 **p=0.009** partial η^2=**0.102**
Time × Context	F(4.26,127.8)=0.055 p=0.996 partial η^2=0.002
Diet × Context	F(1,30)=1.156 p=0.291 partial η^2=0.037
Time × Diet × Context	F(4.26,127.8)=2.173 p=0.072 partial η^2=0.068

S17F Table

**Males**	**Experiment 3 – Body Weight Change**
Diet	F(1,28)=1.021 p=0.321 partial η^2=0.035
Context	F(1,28)=0.231 p=0.634 partial η^2=0.008
Time	F(3.86,107.9)=6.806 **p<0.001** partial η^2=**0.196**
Time × Diet	F(3.86,107.9)=1.575 p=0.188 partial η^2=0.053
Time × Context	F(3.86,107.9)=1.271 p=0.287 partial η^2=0.043
Diet × Context	F(1,28)=0.002 p=0.962 partial η^2=0.000
Time × Diet × Context	F(3.86,107.9)=1.244 p=0.297 partial η^2=0.043
