# Supplementary material for: Generalization of contextual fear is sex-specifically affected by high salt intake
Source: PLoS One. 2023 Jul 13;18(7):e0286221. doi: 10.1371/journal.pone.0286221 (PMC10343085; doi:10.1371/journal.pone.0286221)
Supplement: S18 Table — (PDF) [file pone.0286221.s018.pdf]

Supplemental Material for

Generalization of contextual fear is sex-specifically affected by high salt intake

Jasmin N. Beaver[1,2], Brady L. Weber[1,2], Matthew T. Ford[1], Anna E. Anello[1,2], Kaden M. Ruffin[1], Sarah K. Kassis[1,2], T. Lee Gilman[1,2,3]*

[1]Department of Psychological Sciences, Kent State University, Kent, Ohio, United States of America

[2]Brain Health Research Institute, Kent State University, Kent, Ohio, United States of America

[3]Healthy Communities Research Institute, Kent State University, Kent, Ohio, United States of America

*Corresponding Author

Email: lgilman1@kent.edu (TLG)

S18 Table. Three-way repeated measures ANOVAs on twice weekly body weight measurements of context fear conditioned mice across Experiments.

S18A Table

Females	Experiment 1 – Body Weight		
Diet	$F(1,30)=0.388$	$p=0.538$	partial $\eta^2=0.013$
Context	$F(1,30)=2.823$	$p=0.103$	partial $\eta^2=0.086$
Time	$F(3.24,97.25)=30.28$	**$p<0.001$**	partial $\eta^2=$**0.502**
Time × Diet	$F(3.24,97.25)=1.859$	$p=0.137$	partial $\eta^2=0.058$
Time × Context	$F(3.24,97.25)=0.767$	$p=0.524$	partial $\eta^2=0.025$
Diet × Context	$F(1,30)=0.015$	$p=0.904$	partial $\eta^2=0.000$
Time × Diet × Context	$F(3.24,97.25)=1.035$	$p=0.384$	partial $\eta^2=0.033$

S18B Table

Males	Experiment 1 – Body Weight		
Diet	$F(1,29)=0.316$	$p=0.579$	partial $\eta^2=0.011$
Context	$F(1,29)=0.061$	$p=0.806$	partial $\eta^2=0.002$
Time	$F(2.43,70.56)=9.973$	**$p<0.001$**	partial $\eta^2=$**0.256**
Time × Diet	$F(2.43,70.56)=0.357$	$p=0.742$	partial $\eta^2=0.012$
Time × Context	$F(2.43,70.56)=1.979$	$p=0.137$	partial $\eta^2=0.064$
Diet × Context	$F(1,29)=0.076$	$p=0.785$	partial $\eta^2=0.003$
Time × Diet × Context	$F(2.43,70.56)=0.962$	$p=0.401$	partial $\eta^2=0.032$

S18C Table

Females	Experiment 2 – Body Weight		
Diet	$F(1,30)=0.074$	$p=0.787$	partial $\eta^2=0.002$
Context	$F(1,30)=0.369$	$p=0.548$	partial $\eta^2=0.012$
Time	$F(4.96,148.9)=40.23$	$p<0.001$	partial $\eta^2=0.573$
Time × Diet	$F(4.96,148.9)=2.823$	**$p=0.018$**	partial $\eta^2=$**0.086**
Time × Context	$F(4.96,148.9)=1.563$	$p=0.175$	partial $\eta^2=0.050$
Diet × Context	$F(1,30)=1.463$	$p=0.236$	partial $\eta^2=0.046$
Time × Diet × Context	$F(4.96,148.9)=0.356$	$p=0.876$	partial $\eta^2=0.012$

S18D Table

Males	Experiment 2 – Body Weight		
Diet	$F(1,32)=0.006$	$p=0.937$	partial $\eta^2=0.000$
Context	$F(1,32)=0.063$	$p=0.803$	partial $\eta^2=0.002$
Time	$F(2.28,73.02)=4.422$	**$p=0.012$**	partial $\eta^2=$**0.121**
Time × Diet	$F(2.28,73.02)=2.370$	$p=0.093$	partial $\eta^2=0.069$
Time × Context	$F(2.28,73.02)=0.784$	$p=0.476$	partial $\eta^2=0.024$

Diet × Context	$F(1,32)=1.073$	$p=0.308$	partial $\eta^2=0.032$
Time × Diet × Context	$F(2.28,73.02)=0.383$	$p=0.710$	partial $\eta^2=0.012$

S18E Table

**Females**	**Experiment 3 – Body Weight**		
Diet	$F(1,30)=2.455$	$p=0.128$	partial $\eta^2=0.076$
Context	$F(1,30)=3.497$	$p=0.071$	partial $\eta^2=0.104$
Time	$F(4.57,137.0)=51.87$	$\mathbf{p<0.001}$	partial $\eta^2=\mathbf{0.634}$
Time × Diet	$F(4.57,137.0)=1.496$	$p=0.200$	partial $\eta^2=0.047$
Time × Context	$F(4.57,137.0)=0.288$	$p=0.906$	partial $\eta^2=0.010$
Diet × Context	$F(1,30)=2.245$	$p=0.145$	partial $\eta^2=0.070$
Time × Diet × Context	$F(4.57,137.0)=1.660$	$p=0.154$	partial $\eta^2=0.052$

S18F Table

**Males**	**Experiment 3 – Body Weight**		
Diet	$F(1,28)=0.120$	$p=0.732$	partial $\eta^2=0.004$
Context	$F(1,28)=1.016$	$p=0.322$	partial $\eta^2=0.035$
Time	$F(2.67,74.86)=12.22$	$p<0.001$	partial $\eta^2=0.304$
Time × Diet	$F(2.67,74.86)=3.019$	$\mathbf{p=0.040}$	partial $\eta^2=\mathbf{0.097}$
Time × Context	$F(2.67,74.86)=0.426$	$p=0.713$	partial $\eta^2=0.015$
Diet × Context	$F(1,28)=0.150$	$p=0.701$	partial $\eta^2=0.005$
Time × Diet × Context	$F(2.67,74.86)=0.422$	$p=0.715$	partial $\eta^2=0.015$
