# Supplementary material for: Generalization of contextual fear is sex-specifically affected by high salt intake
Source: PLoS One. 2023 Jul 13;18(7):e0286221. doi: 10.1371/journal.pone.0286221 (PMC10343085; doi:10.1371/journal.pone.0286221)
Supplement: S19 Table — (PDF) [file pone.0286221.s019.pdf]

Supplemental Material for

Generalization of contextual fear is sex-specifically affected by high salt intake

Jasmin N. Beaver[1,2], Brady L. Weber[1,2], Matthew T. Ford[1], Anna E. Anello[1,2], Kaden M. Ruffin[1], Sarah K. Kassis[1,2], T. Lee Gilman[1,2,3*]

[1]Department of Psychological Sciences, Kent State University, Kent, Ohio, United States of America

[2]Brain Health Research Institute, Kent State University, Kent, Ohio, United States of America

[3]Healthy Communities Research Institute, Kent State University, Kent, Ohio, United States of America

*Corresponding Author

Email: lgilman1@kent.edu (TLG)

S19 Table. Three-way repeated measures ANOVAs on weekly average water consumption per day for control no shock mice across Experiments.

S19A Table

Experiment 1	Water/day		
Sex	F(1,31)=3.245	p=0.081	partial η^2=0.095
Diet	F(1,31)=50.99	**p<0.001**	partial η^2=**0.622**
Time	F(1.67,51.69)=3.219	p=0.057	partial η^2=0.094
Time × Sex	F(1.67,51.69)=4.157	**p=0.027**	partial η^2=**0.118**
Time × Diet	F(1.67,51.69)=1.459	p=0.242	partial η^2=0.045
Sex × Diet	F(1,31)=3.868	p=0.058	partial η^2=0.111
Time × Sex × Diet	F(1.67,51.69)=0.910	p=0.393	partial η^2=0.029

S19B Table

Experiment 2	Water/day		
Sex	F(1,29)=9.248	p=0.005	partial η^2=0.242
Diet	F(1,29)=204.6	p<0.001	partial η^2=0.876
Time	F(3.98,115.4)=17.93	p<0.001	partial η^2=0.382
Time × Sex	F(3.98,115.4)=6.510	**p<0.001**	partial η^2=**0.183**
Time × Diet	F(3.98,115.4)=5.307	**p<0.001**	partial η^2=**0.155**
Sex × Diet	F(1,29)=0.231	p=0.635	partial η^2=0.008
Time × Sex × Diet	F(3.98,115.4)=0.084	p=0.987	partial η^2=0.003

S19C Table

Experiment 3	Water/day		
Sex	F(1,28)=0.188	p=0.668	partial η^2=0.007
Diet	F(1,28)=37.59	p<0.001	partial η^2=0.573
Time	F(3.47,97.21)=5.224	p=0.001	partial η^2=0.157
Time × Sex	F(3.47,97.21)=0.962	p=0.423	partial η^2=0.033
Time × Diet	F(3.47,97.21)=3.541	p=0.013	partial η^2=0.112
Sex × Diet	F(1,28)=3.551	p=0.070	partial η^2=0.113
Time × Sex × Diet	F(3.47,97.21)=3.387	**p=0.016**	partial η^2=**0.108**
