# Supplementary material for: Generalization of contextual fear is sex-specifically affected by high salt intake
Source: PLoS One. 2023 Jul 13;18(7):e0286221. doi: 10.1371/journal.pone.0286221 (PMC10343085; doi:10.1371/journal.pone.0286221)
Supplement: S20 Table — (PDF) [file pone.0286221.s020.pdf]

Supplemental Material for

Generalization of contextual fear is sex-specifically affected by high salt intake

Jasmin N. Beaver[1,2], Brady L. Weber[1,2], Matthew T. Ford[1], Anna E. Anello[1,2], Kaden M. Ruffin[1], Sarah K. Kassis[1,2], T. Lee Gilman[1,2,3*]

[1]Department of Psychological Sciences, Kent State University, Kent, Ohio, United States of America

[2]Brain Health Research Institute, Kent State University, Kent, Ohio, United States of America

[3]Healthy Communities Research Institute, Kent State University, Kent, Ohio, United States of America

*Corresponding Author

Email: lgilman1@kent.edu (TLG)

S20 Table. Three-way repeated measures ANOVAs on weekly average food consumption per day for control no shock mice across Experiments.

S20A Table

Experiment 1	Food/day		
Sex	F(1,31)=17.58	**p<0.001**	partial η^2=**0.362**
Diet	F(1,31)=8.329	**p=0.007**	partial η^2=**0.212**
Time	F(1.56,48.19)=0.003	p=0.990	partial η^2=0.000
Time × Sex	F(1.56,48.19)=2.401	p=0.113	partial η^2=0.072
Time × Diet	F(1.56,48.19)=0.223	p=0.745	partial η^2=0.007
Sex × Diet	F(1,31)=0.130	p=0.721	partial η^2=0.004
Time × Sex × Diet	F(1.56,48.19)=1.258	p=0.286	partial η^2=0.039

S20B Table

Experiment 2	Food/day		
Sex	F(1,29)=22.47	**p<0.001**	partial η^2=**0.437**
Diet	F(1,29)=15.77	p<0.001	partial η^2=0.352
Time	F(3.08,89.34)=2.245	p=0.087	partial η^2=0.072
Time × Sex	F(3.08,89.34)=0.476	p=0.705	partial η^2=0.016
Time × Diet	F(3.08,89.34)=3.463	**p=0.019**	partial η^2=**0.107**
Sex × Diet	F(1,29)=0.969	p=0.333	partial η^2=0.032
Time × Sex × Diet	F(3.08,89.34)=0.617	p=0.610	partial η^2=0.021

S20C Table

Experiment 3	Food/day		
Sex	F(1,28)=24.30	**p<0.001**	partial η^2=**0.465**
Diet	F(1,28)=7.983	**p=0.009**	partial η^2=**0.222**
Time	F(2.76,77.39)=1.692	p=0.179	partial η^2=0.057
Time × Sex	F(2.76,77.39)=1.523	p=0.218	partial η^2=0.052
Time × Diet	F(2.76,77.39)=1.496	p=0.224	partial η^2=0.051
Sex × Diet	F(1,28)=0.735	p=0.398	partial η^2=0.026
Time × Sex × Diet	F(2.76,77.39)=1.174	p=0.324	partial η^2=0.040
