# Supplementary material for: Generalization of contextual fear is sex-specifically affected by high salt intake
Source: PLoS One. 2023 Jul 13;18(7):e0286221. doi: 10.1371/journal.pone.0286221 (PMC10343085; doi:10.1371/journal.pone.0286221)
Supplement: S21 Table — (PDF) [file pone.0286221.s021.pdf]

Supplemental Material for

Generalization of contextual fear is sex-specifically affected by high salt intake

Jasmin N. Beaver[1,2], Brady L. Weber[1,2], Matthew T. Ford[1], Anna E. Anello[1,2], Kaden M. Ruffin[1], Sarah K. Kassis[1,2], T. Lee Gilman[1,2,3]*

[1]Department of Psychological Sciences, Kent State University, Kent, Ohio, United States of America

[2]Brain Health Research Institute, Kent State University, Kent, Ohio, United States of America

[3]Healthy Communities Research Institute, Kent State University, Kent, Ohio, United States of America

*Corresponding Author

Email: lgilman1@kent.edu (TLG)

S21 Table. Three-way repeated measures ANOVAs on weekly body weight changes for control no shock mice across Experiments.

S21A Table

**Experiment 1**	**Body Weight Change**		
Sex	$F(1,31)=0.236$	$p=0.631$	partial $\eta^2=0.008$
Diet	$F(1,31)=0.443$	$p=0.511$	partial $\eta^2=0.014$
Time	$F(1.84,42.05)=59.12$	**$p<0.001$**	partial $\eta^2=$**0.656**
Time × Sex	$F(1.84,42.05)=0.032$	$p=0.960$	partial $\eta^2=0.001$
Time × Diet	$F(1.84,42.05)=1.319$	$p=0.274$	partial $\eta^2=0.041$
Sex × Diet	$F(1,31)=6.755$	**$p=0.014$**	partial $\eta^2=$**0.179**
Time × Sex × Diet	$F(1.84,42.05)=0.491$	$p=0.599$	partial $\eta^2=0.016$

S21B Table

**Experiment 2**	**Body Weight Change**		
Sex	$F(1,29)=0.966$	$p=0.334$	partial $\eta^2=0.032$
Diet	$F(1,29)=1.596$	$p=0.216$	partial $\eta^2=0.052$
Time	$F(4.00,115.9)=29.74$	$p<0.001$	partial $\eta^2=0.506$
Time × Sex	$F(4.00,115.9)=4.637$	**$p=0.002$**	partial $\eta^2=$**0.138**
Time × Diet	$F(4.00,115.9)=3.270$	**$p=0.014$**	partial $\eta^2=$**0.101**
Sex × Diet	$F(1,29)=0.562$	$p=0.460$	partial $\eta^2=0.019$
Time × Sex × Diet	$F(4.00,115.9)=0.530$	$p=0.713$	partial $\eta^2=0.018$

S21C Table

**Experiment 3**	**Body Weight Change**		
Sex	$F(1,28)=6.100$	$p=0.020$	partial $\eta^2=0.179$
Diet	$F(1,28)=3.939$	$p=0.057$	partial $\eta^2=0.123$
Time	$F(3.14,87.80)=18.35$	$p<0.001$	partial $\eta^2=0.396$
Time × Sex	$F(3.14,87.80)=4.494$	**$p=0.005$**	partial $\eta^2=$**0.138**
Time × Diet	$F(3.14,87.80)=4.231$	**$p=0.007$**	partial $\eta^2=$**0.131**
Sex × Diet	$F(1,28)=0.136$	$p=0.715$	partial $\eta^2=0.005$
Time × Sex × Diet	$F(3.14,87.80)=1.041$	$p=0.380$	partial $\eta^2=0.036$
