# Supplementary material for: Generalization of contextual fear is sex-specifically affected by high salt intake
Source: PLoS One. 2023 Jul 13;18(7):e0286221. doi: 10.1371/journal.pone.0286221 (PMC10343085; doi:10.1371/journal.pone.0286221)
Supplement: S22 Table — (PDF) [file pone.0286221.s022.pdf]

Supplemental Material for

Generalization of contextual fear is sex-specifically affected by high salt intake

Jasmin N. Beaver[1,2], Brady L. Weber[1,2], Matthew T. Ford[1], Anna E. Anello[1,2], Kaden M. Ruffin[1], Sarah K. Kassis[1,2], T. Lee Gilman[1,2,3]*

[1]Department of Psychological Sciences, Kent State University, Kent, Ohio, United States of America

[2]Brain Health Research Institute, Kent State University, Kent, Ohio, United States of America

[3]Healthy Communities Research Institute, Kent State University, Kent, Ohio, United States of America

*Corresponding Author

Email: lgilman1@kent.edu (TLG)

S22 Table. Three-way repeated measures ANOVAs on twice weekly body weights of control no shock mice across Experiments.

S22A Table

Experiment 1	Body Weight		
Sex	$F(1,31)=25.84$	$p<0.001$	partial $\eta^2=0.455$
Diet	$F(1,31)=0.018$	$p=0.893$	partial $\eta^2=0.001$
Time	$F(2.31,71.60)=21.32$	$p<0.001$	partial $\eta^2=0.408$
Time × Sex	$F(2.31,71.60)=0.174$	$p=0.869$	partial $\eta^2=0.006$
Time × Diet	$F(2.31,71.60)=0.667$	$p=0.537$	partial $\eta^2=0.021$
Sex × Diet	$F(1,31)=0.155$	$p=0.697$	partial $\eta^2=0.179$
Time × Sex × Diet	$F(2.31,71.60)=3.707$	**$p=0.024$**	partial $\eta^2=$**0.107**

S22B Table

Experiment 2	Body Weight		
Sex	$F(1,29)=38.62$	$p<0.001$	partial $\eta^2=0.571$
Diet	$F(1,29)=2.447$	$p=0.129$	partial $\eta^2=0.078$
Time	$F(3.30,95.56)=13.28$	$p<0.001$	partial $\eta^2=0.314$
Time × Sex	$F(3.30,95.56)=4.031$	**$p=0.008$**	partial $\eta^2=$**0.122**
Time × Diet	$F(3.30,95.56)=2.077$	$p=0.102$	partial $\eta^2=0.067$
Sex × Diet	$F(1,29)=0.418$	$p=0.523$	partial $\eta^2=0.014$
Time × Sex × Diet	$F(3.30,95.56)=0.961$	$p=0.421$	partial $\eta^2=0.032$

S22C Table

Experiment 3	Body Weight		
Sex	$F(1,28)=59.03$	$p<0.001$	partial $\eta^2=0.678$
Diet	$F(1,28)=1.095$	$p=0.304$	partial $\eta^2=0.038$
Time	$F(2.64,73.82)=11.92$	$p<0.001$	partial $\eta^2=0.299$
Time × Sex	$F(2.64,73.82)=3.879$	**$p=0.016$**	partial $\eta^2=$**0.122**
Time × Diet	$F(2.64,73.82)=2.216$	$p=0.101$	partial $\eta^2=0.073$
Sex × Diet	$F(1,28)=0.039$	$p=0.845$	partial $\eta^2=0.001$
Time × Sex × Diet	$F(2.64,73.82)=0.564$	$p=0.618$	partial $\eta^2=0.020$
