# Supplementary material for: Generalization of contextual fear is sex-specifically affected by high salt intake
Source: PLoS One. 2023 Jul 13;18(7):e0286221. doi: 10.1371/journal.pone.0286221 (PMC10343085; doi:10.1371/journal.pone.0286221)
Supplement: S23 Table — (PDF) [file pone.0286221.s023.pdf]

Supplemental Material for

Generalization of contextual fear is sex-specifically affected by high salt intake

Jasmin N. Beaver[1,2], Brady L. Weber[1,2], Matthew T. Ford[1], Anna E. Anello[1,2], Kaden M. Ruffin[1], Sarah K. Kassis[1,2], T. Lee Gilman[1,2,3]*

[1]Department of Psychological Sciences, Kent State University, Kent, Ohio, United States of America

[2]Brain Health Research Institute, Kent State University, Kent, Ohio, United States of America

[3]Healthy Communities Research Institute, Kent State University, Kent, Ohio, United States of America

*Corresponding Author

Email: lgilman1@kent.edu (TLG)

S23 Table. Three-way repeated measures ANOVAs on weekly average NaCl consumed per day by context fear conditioned mice across Experiments.

S23A Table

Females	Experiment 1 – NaCl/day		
Diet	$F(1,30)=635.7$	**$p<0.001$**	partial η^2=**0.955**
Context	$F(1,30)=0.004$	$p=0.951$	partial $\eta^2=0.000$
Time	$F(1.61,48.36)=0.846$	$p=0.413$	partial $\eta^2=0.027$
Time × Diet	$F(1.61,48.36)=0.834$	$p=0.418$	partial $\eta^2=0.027$
Time × Context	$F(1.61,48.36)=0.698$	$p=0.473$	partial $\eta^2=0.023$
Diet × Context	$F(1,30)=0.029$	$p=0.866$	partial $\eta^2=0.001$
Time × Diet × Context	$F(1.61,48.36)=0.627$	$p=0.505$	partial $\eta^2=0.020$

S23B Table

Males	Experiment 1 – NaCl/day		
Diet	$F(1,29)=784.5$	**$p<0.001$**	partial η^2=**0.964**
Context	$F(1,29)=0.017$	$p=0.899$	partial $\eta^2=0.001$
Time	$F(1.64,47.50)=0.759$	$p=0.449$	partial $\eta^2=0.025$
Time × Diet	$F(1.64,47.50)=0.377$	$p=0.646$	partial $\eta^2=0.013$
Time × Context	$F(1.64,47.50)=0.312$	$p=0.690$	partial $\eta^2=0.011$
Diet × Context	$F(1,29)=0.014$	$p=0.906$	partial $\eta^2=0.000$
Time × Diet × Context	$F(1.64,47.50)=0.497$	$p=0.574$	partial $\eta^2=0.017$

S23C Table

Females	Experiment 2 – NaCl/day		
Diet	$F(1,30)=1412$	$p<0.001$	partial $\eta^2=0.979$
Context	$F(1,30)=0.000$	$p=0.988$	partial $\eta^2=0.000$
Time	$F(3.22,96.53)=6.807$	$p<0.001$	partial $\eta^2=0.185$
Time × Diet	$F(3.22,96.53)=4.373$	**$p=0.005$**	partial η^2=**0.127**
Time × Context	$F(3.22,96.53)=0.929$	$p=0.435$	partial $\eta^2=0.030$
Diet × Context	$F(1,30)=0.002$	$p=0.962$	partial $\eta^2=0.000$
Time × Diet × Context	$F(3.22,96.53)=0.760$	$p=0.528$	partial $\eta^2=0.025$

S23D Table

Males	Experiment 2 – NaCl/day		
Diet	$F(1,32)=2164$	$p<0.001$	partial $\eta^2=0.985$
Context	$F(1,32)=0.904$	$p=0.349$	partial $\eta^2=0.027$
Time	$F(4.11,131.4)=6.013$	$p<0.001$	partial $\eta^2=0.158$
Time × Diet	$F(4.11,131.4)=4.350$	**$p=0.002$**	partial η^2=**0.120**
Time × Context	$F(4.11,131.4)=0.943$	$p=0.443$	partial $\eta^2=0.029$

Diet × Context	F(1,32)=1.057	p=0.312	partial η^2=0.032
Time × Diet × Context	F(4.11,131.4)=1.003	p=0.410	partial η^2=0.030

S23E Table

**Females**	**Experiment 3 – NaCl/day**		
Diet	F(1,30)=621.9	**p<0.001**	partial η^2=**0.954**
Context	F(1,30)=0.301	p=0.587	partial η^2=0.010
Time	F(2.27,68.08)=1.194	p=0.313	partial η^2=0.038
Time × Diet	F(2.27,68.08)=0.710	p=0.512	partial η^2=0.023
Time × Context	F(2.27,68.08)=0.192	p=0.852	partial η^2=0.006
Diet × Context	F(1,30)=0.177	p=0.677	partial η^2=0.006
Time × Diet × Context	F(2.27,68.08)=0.190	p=0.853	partial η^2=0.006

S23F Table

**Males**	**Experiment 3 – NaCl/day**		
Diet	F(1,28)=398.8	**p<0.001**	partial η^2=**0.934**
Context	F(1,28)=0.186	p=0.670	partial η^2=0.007
Time	F(2.94,82.41)=2.553	p=0.062	partial η^2=0.084
Time × Diet	F(2.94,82.41)=1.989	p=0.123	partial η^2=0.066
Time × Context	F(2.94,82.41)=0.723	p=0.538	partial η^2=0.025
Diet × Context	F(1,28)=0.244	p=0.625	partial η^2=0.009
Time × Diet × Context	F(2.94,82.41)=0.626	p=0.597	partial η^2=0.022
