# Supplementary material for: Generalization of contextual fear is sex-specifically affected by high salt intake
Source: PLoS One. 2023 Jul 13;18(7):e0286221. doi: 10.1371/journal.pone.0286221 (PMC10343085; doi:10.1371/journal.pone.0286221)
Supplement: S24 Table — (PDF) [file pone.0286221.s024.pdf]

Supplemental Material for

Generalization of contextual fear is sex-specifically affected by high salt intake

Jasmin N. Beaver[1,2], Brady L. Weber[1,2], Matthew T. Ford[1], Anna E. Anello[1,2], Kaden M. Ruffin[1], Sarah K. Kassis[1,2], T. Lee Gilman[1,2,3*]

[1]Department of Psychological Sciences, Kent State University, Kent, Ohio, United States of America

[2]Brain Health Research Institute, Kent State University, Kent, Ohio, United States of America

[3]Healthy Communities Research Institute, Kent State University, Kent, Ohio, United States of America

*Corresponding Author

Email: lgilman1@kent.edu (TLG)

S24 Table. Three-way repeated measures ANOVAs on weekly average NaCl consumed as a percentage of body weight in context fear conditioned mice across Experiments.

S24A Table

**Females**	**Experiment 1 – NaCl as % BW**		
Diet	F(1,30)=532.7	**p<0.001**	partial η²=**0.947**
Context	F(1,30)=0.408	p=0.528	partial η²=0.013
Time	F(1.51,45.38)=2.849	p=0.082	partial η²=0.087
Time × Diet	F(1.51,45.38)=2.372	p=0.117	partial η²=0.073
Time × Context	F(1.51,45.38)=0.648	p=0.487	partial η²=0.021
Diet × Context	F(1,30)=0.378	p=0.544	partial η²=0.012
Time × Diet × Context	F(1.51,45.38)=0.578	p=0.519	partial η²=0.019

S24B Table

**Males**	**Experiment 1 – NaCl as % BW**		
Diet	F(1,29)=496.0	**p<0.001**	partial η²=**0.945**
Context	F(1,29)=0.002	p=0.968	partial η²=0.000
Time	F(1.75,50.83)=0.847	p=0.421	partial η²=0.028
Time × Diet	F(1.75,50.83)=0.841	p=0.424	partial η²=0.028
Time × Context	F(1.75,50.83)=0.937	p=0.388	partial η²=0.031
Diet × Context	F(1,29)=0.005	p=0.944	partial η²=0.000
Time × Diet × Context	F(1.75,50.83)=1.047	p=0.351	partial η²=0.035

S24C Table

**Females**	**Experiment 2 – NaCl as % BW**		
Diet	F(1,30)=1804	**p<0.001**	partial η²=**0.984**
Context	F(1,30)=0.102	p=0.752	partial η²=0.003
Time	F(3.02,90.73)=2.635	p=0.054	partial η²=0.081
Time × Diet	F(3.02,90.73)=1.623	p=0.189	partial η²=0.051
Time × Context	F(3.02,90.73)=1.068	p=0.367	partial η²=0.034
Diet × Context	F(1,30)=0.162	p=0.690	partial η²=0.005
Time × Diet × Context	F(3.02,90.73)=0.897	p=0.447	partial η²=0.029

S24D Table

**Males**	**Experiment 2 – NaCl as % BW**		
Diet	F(1,32)=819.4	p<0.001	partial η²=0.962
Context	F(1,32)=1.631	p=0.211	partial η²=0.048
Time	F(3.82,122.1)=4.817	p=0.001	partial η²=0.131
Time × Diet	F(3.82,122.1)=3.901	**p=0.006**	partial η²=**0.109**
Time × Context	F(3.82,122.1)=0.665	p=0.610	partial η²=0.020

Diet × Context	$F(1,32)=1.895$ $p=0.178$ partial $\eta^2=0.056$
Time × Diet × Context	$F(3.82,122.1)=0.665$ $p=0.610$ partial $\eta^2=0.020$

S24E Table

**Females**	**Experiment 3 – NaCl as % BW**
Diet	$F(1,30)=873.8$ **$p<0.001$** partial $\eta^2=$**0.967**
Context	$F(1,30)=0.439$ $p=0.513$ partial $\eta^2=0.014$
Time	$F(1.92,57.61)=1.531$ $p=0.225$ partial $\eta^2=0.049$
Time × Diet	$F(1.92,57.61)=0.948$ $p=0.390$ partial $\eta^2=0.031$
Time × Context	$F(1.92,57.61)=0.237$ $p=0.781$ partial $\eta^2=0.008$
Diet × Context	$F(1,30)=0.621$ $p=0.437$ partial $\eta^2=0.020$
Time × Diet × Context	$F(1.92,57.61)=0.197$ $p=0.813$ partial $\eta^2=0.007$

S24F Table

**Males**	**Experiment 3 – NaCl as % BW**
Diet	$F(1,28)=772.2$ **$p<0.001$** partial $\eta^2=$**0.965**
Context	$F(1,28)=0.941$ $p=0.340$ partial $\eta^2=0.033$
Time	$F(2.75,76.95)=3.738$ **$p=0.017$** partial $\eta^2=$**0.118**
Time × Diet	$F(2.75,76.95)=2.772$ $p=0.052$ partial $\eta^2=0.090$
Time × Context	$F(2.75,76.95)=0.699$ $p=0.544$ partial $\eta^2=0.024$
Diet × Context	$F(1,28)=0.807$ $p=0.377$ partial $\eta^2=0.028$
Time × Diet × Context	$F(2.75,76.95)=0.627$ $p=0.586$ partial $\eta^2=0.022$
