# Supplementary material for: Generalization of contextual fear is sex-specifically affected by high salt intake
Source: PLoS One. 2023 Jul 13;18(7):e0286221. doi: 10.1371/journal.pone.0286221 (PMC10343085; doi:10.1371/journal.pone.0286221)
Supplement: S25 Table — (PDF) [file pone.0286221.s025.pdf]

Supplemental Material for

Generalization of contextual fear is sex-specifically affected by high salt intake

Jasmin N. Beaver[1,2], Brady L. Weber[1,2], Matthew T. Ford[1], Anna E. Anello[1,2], Kaden M. Ruffin[1], Sarah K. Kassis[1,2], T. Lee Gilman[1,2,3]*

[1]Department of Psychological Sciences, Kent State University, Kent, Ohio, United States of America

[2]Brain Health Research Institute, Kent State University, Kent, Ohio, United States of America

[3]Healthy Communities Research Institute, Kent State University, Kent, Ohio, United States of America

*Corresponding Author

Email: lgilman1@kent.edu (TLG)

S25 Table. Three-way repeated measures ANOVAs on weekly ratio of water to NaCl consumed in context fear conditioned mice across Experiments.

S25A Table

**Females**	**Experiment 1 – Water:NaCl Ratio**		
Diet	$F(1,30)=158.3$	**$p<0.001$**	partial η^2=**0.841**
Context	$F(1,30)=0.020$	$p=0.890$	partial $\eta^2=0.001$
Time	$F(1.86,55.66)=1.501$	$p=0.232$	partial $\eta^2=0.048$
Time × Diet	$F(1.86,55.66)=1.044$	$p=0.354$	partial $\eta^2=0.034$
Time × Context	$F(1.86,55.66)=0.511$	$p=0.589$	partial $\eta^2=0.017$
Diet × Context	$F(1,30)=0.000$	$p=0.996$	partial $\eta^2=0.000$
Time × Diet × Context	$F(1.86,55.66)=0.264$	$p=0.752$	partial $\eta^2=0.009$

S25B Table

**Males**	**Experiment 1 – Water:NaCl Ratio**		
Diet	$F(1,26)=88.66$	$p<0.001$	partial $\eta^2=0.773$
Context	$F(1,26)=0.351$	$p=0.559$	partial $\eta^2=0.013$
Time	$F(1.91,49.53)=13.16$	$p<0.001$	partial $\eta^2=0.336$
Time × Diet	$F(1.91,49.53)=11.52$	**$p<0.001$**	partial η^2=**0.307**
Time × Context	$F(1.91,49.53)=0.923$	$p=0.400$	partial $\eta^2=0.034$
Diet × Context	$F(1,26)=0.896$	$p=0.353$	partial $\eta^2=0.033$
Time × Diet × Context	$F(1.91,49.53)=1.376$	$p=0.262$	partial $\eta^2=0.050$

S25C Table

**Females**	**Experiment 2 – Water:NaCl Ratio**		
Diet	$F(1,29)=417.8$	$p<0.001$	partial $\eta^2=0.935$
Context	$F(1,29)=0.839$	$p=0.367$	partial $\eta^2=0.028$
Time	$F(3.96,114.7)=4.894$	$p=0.001$	partial $\eta^2=0.144$
Time × Diet	$F(3.96,114.7)=4.698$	**$p=0.002$**	partial η^2=**0.139**
Time × Context	$F(3.96,114.7)=0.952$	$p=0.436$	partial $\eta^2=0.032$
Diet × Context	$F(1,29)=1.627$	$p=0.212$	partial $\eta^2=0.053$
Time × Diet × Context	$F(3.96,114.7)=0.792$	$p=0.532$	partial $\eta^2=0.027$

S25D Table

**Males**	**Experiment 2 – Water:NaCl Ratio**		
Diet	$F(1,31)=154.6$	$p<0.001$	partial $\eta^2=0.833$
Context	$F(1,31)=1.027$	$p=0.319$	partial $\eta^2=0.032$
Time	$F(3.70,114.6)=8.018$	$p<0.001$	partial $\eta^2=0.205$
Time × Diet	$F(1.17,37.97)=4.476$	**$p=0.003$**	partial η^2=**0.126**
Time × Context	$F(1.17,37.97)=0.161$	$p=0.949$	partial $\eta^2=0.005$

Diet × Context	F(1,31)=0.615 p=0.439 partial η^2=0.019
Time × Diet × Context	F(1.17,37.97)=0.170 p=0.945 partial η^2=0.005

S25E Table

**Females**	**Experiment 3 – Water:NaCl Ratio**
Diet	F(1,28)=225.1 p<0.001 partial η^2=0.889
Context	F(1,28)=0.184 p=0.671 partial η^2=0.007
Time	F(2.64,73.98)=4.532 p=0.008 partial η^2=0.139
Time × Diet	F(2.64,73.98)=3.094 **p=0.038** partial η^2=**0.100**
Time × Context	F(2.64,73.98)=0.358 p=0.758 partial η^2=0.013
Diet × Context	F(1,28)=0.165 p=0.687 partial η^2=0.006
Time × Diet × Context	F(2.64,73.98)=0.394 p=0.732 partial η^2=0.014

S25F Table

**Males**	**Experiment 3 – Water:NaCl Ratio**
Diet	F(1,27)=212.8 p<0.001 partial η^2=0.887
Context	F(1,27)=0.999 p=0.326 partial η^2=0.036
Time	F(2.82,76.01)=8.667 p<0.001 partial η^2=0.243
Time × Diet	F(2.82,76.01)=4.683 **p=0.006** partial η^2=**0.148**
Time × Context	F(2.82,76.01)=1.027 p=0.382 partial η^2=0.037
Diet × Context	F(1,27)=1.140 p=0.295 partial η^2=0.041
Time × Diet × Context	F(2.82,76.01)=0.879 p=0.450 partial η^2=0.032
