# Supplementary material for: Generalization of contextual fear is sex-specifically affected by high salt intake
Source: PLoS One. 2023 Jul 13;18(7):e0286221. doi: 10.1371/journal.pone.0286221 (PMC10343085; doi:10.1371/journal.pone.0286221)
Supplement: S26 Table — (PDF) [file pone.0286221.s026.pdf]

Supplemental Material for

Generalization of contextual fear is sex-specifically affected by high salt intake

Jasmin N. Beaver[1,2], Brady L. Weber[1,2], Matthew T. Ford[1], Anna E. Anello[1,2], Kaden M. Ruffin[1], Sarah K. Kassis[1,2], T. Lee Gilman[1,2,3*]

[1]Department of Psychological Sciences, Kent State University, Kent, Ohio, United States of America

[2]Brain Health Research Institute, Kent State University, Kent, Ohio, United States of America

[3]Healthy Communities Research Institute, Kent State University, Kent, Ohio, United States of America

*Corresponding Author

Email: lgilman1@kent.edu (TLG)

S26 Table. Three-way repeated measures ANOVAs on weekly average kcal consumed per day by context fear conditioned mice across Experiments.

S26A Table

**Females**	**Experiment 1 – kcal/day**		
Diet	F(1,30)=3.622	p=**0.067**	partial η^2=**0.108**
Context	F(1,30)=0.105	p=0.749	partial η^2=0.003
Time	F(1.67,50.22)=0.640	p=0.505	partial η^2=0.021
Time × Diet	F(1.67,50.22)=0.568	p=0.540	partial η^2=0.019
Time × Context	F(1.67,50.22)=1.251	p=0.290	partial η^2=0.040
Diet × Context	F(1,30)=0.244	p=0.625	partial η^2=0.008
Time × Diet × Context	F(1.67,50.22)=0.823	p=0.426	partial η^2=0.027

S26B Table

**Males**	**Experiment 1 – kcal/day**		
Diet	F(1,29)=0.204	p=0.655	partial η^2=0.007
Context	F(1,29)=0.013	p=0.911	partial η^2=0.000
Time	F(1.85,53.70)=1.833	p=0.172	partial η^2=0.059
Time × Diet	F(1.85,53.70)=0.354	p=0.687	partial η^2=0.012
Time × Context	F(1.85,53.70)=0.148	p=0.847	partial η^2=0.005
Diet × Context	F(1,29)=0.003	p=0.959	partial η^2=0.000
Time × Diet × Context	F(1.85,53.70)=0.866	p=0.419	partial η^2=0.029

S26C Table

**Females**	**Experiment 2 – kcal/day**		
Diet	F(1,30)=27.67	**p<0.001**	partial η^2=**0.480**
Context	F(1,30)=0.016	p=0.901	partial η^2=0.001
Time	F(3.72,111.7)=13.06	**p<0.001**	partial η^2=**0.303**
Time × Diet	F(3.72,111.7)=0.221	p=0.916	partial η^2=0.007
Time × Context	F(3.72,111.7)=1.325	p=0.267	partial η^2=0.042
Diet × Context	F(1,30)=0.034	p=0.856	partial η^2=0.001
Time × Diet × Context	F(3.72,111.7)=0.434	p=0.771	partial η^2=0.014

S26D Table

**Males**	**Experiment 2 – kcal/day**		
Diet	F(1,32)=0.756	p=0.391	partial η^2=0.023
Context	F(1,32)=0.117	p=0.735	partial η^2=0.004
Time	F(4.32,138.1)=10.33	**p<0.001**	partial η^2=**0.244**
Time × Diet	F(4.32,138.1)=1.334	p=0.258	partial η^2=0.040
Time × Context	F(4.32,138.1)=0.436	p=0.797	partial η^2=0.013

Diet × Context	F(1,32)=0.654 p=0.425 partial η^2=0.020
Time × Diet × Context	F(4.32,138.1)=0.759 p=0.563 partial η^2=0.023

S26E Table

**Females**	**Experiment 3 – kcal/day**
Diet	F(1,30)=10.63 **p=0.003** partial η^2=**0.262**
Context	F(1,30)=1.166 p=0.289 partial η^2=0.037
Time	F(2.81,84.25)=5.167 **p=0.003** partial η^2=**0.147**
Time × Diet	F(2.81,84.25)=1.684 p=0.180 partial η^2=0.053
Time × Context	F(2.81,84.25)=0.462 p=0.697 partial η^2=0.015
Diet × Context	F(1,30)=0.029 p=0.866 partial η^2=0.001
Time × Diet × Context	F(2.81,84.25)=0.453 p=0.703 partial η^2=0.015

S26F Table

**Males**	**Experiment 3 – kcal/day**
Diet	F(1,28)=4.534 **p=0.042** partial η^2=**0.139**
Context	F(1,28)=0.014 p=0.906 partial η^2=0.001
Time	F(3.42,95.83)=5.161 **p=0.002** partial η^2=**0.156**
Time × Diet	F(3.42,95.83)=0.990 p=0.408 partial η^2=0.034
Time × Context	F(3.42,95.83)=1.274 p=0.287 partial η^2=0.044
Diet × Context	F(1,28)=0.489 p=0.490 partial η^2=0.017
Time × Diet × Context	F(3.42,95.83)=0.552 p=0.671 partial η^2=0.019
