# Supplementary material for: Generalization of contextual fear is sex-specifically affected by high salt intake
Source: PLoS One. 2023 Jul 13;18(7):e0286221. doi: 10.1371/journal.pone.0286221 (PMC10343085; doi:10.1371/journal.pone.0286221)
Supplement: S27 Table — (PDF) [file pone.0286221.s027.pdf]

Supplemental Material for

Generalization of contextual fear is sex-specifically affected by high salt intake

Jasmin N. Beaver[1,2], Brady L. Weber[1,2], Matthew T. Ford[1], Anna E. Anello[1,2], Kaden M. Ruffin[1], Sarah K. Kassis[1,2], T. Lee Gilman[1,2,3]*

[1]Department of Psychological Sciences, Kent State University, Kent, Ohio, United States of America

[2]Brain Health Research Institute, Kent State University, Kent, Ohio, United States of America

[3]Healthy Communities Research Institute, Kent State University, Kent, Ohio, United States of America

*Corresponding Author

Email: lgilman1@kent.edu (TLG)

S27 Table. Three-way repeated measures ANOVAs on weekly kcal consumed as a percentage of body weight in context fear conditioned mice across Experiments.

S27A Table

Females	Experiment 1 – kcal as % BW		
Diet	$F(1,30)=2.043$	$p=0.163$	partial $\eta^2=0.064$
Context	$F(1,30)=0.320$	$p=0.576$	partial $\eta^2=0.011$
Time	$F(1.61,48.41)=3.279$	$p=0.056$	partial $\eta^2=0.099$
Time × Diet	$F(1.61,48.41)=0.462$	$p=0.591$	partial $\eta^2=0.015$
Time × Context	$F(1.61,48.41)=1.166$	$p=0.311$	partial $\eta^2=0.037$
Diet × Context	$F(1,30)=0.140$	$p=0.710$	partial $\eta^2=0.005$
Time × Diet × Context	$F(1.61,48.41)=0.756$	$p=0.448$	partial $\eta^2=0.025$

S27B Table

Males	Experiment 1 – kcal as % BW		
Diet	$F(1,29)=0.000$	$p=0.993$	partial $\eta^2=0.000$
Context	$F(1,29)=0.006$	$p=0.941$	partial $\eta^2=0.000$
Time	$F(1.71,49.67)=0.479$	$p=0.593$	partial $\eta^2=0.016$
Time × Diet	$F(1.71,49.67)=0.459$	$p=0.605$	partial $\eta^2=0.016$
Time × Context	$F(1.71,49.67)=0.272$	$p=0.729$	partial $\eta^2=0.009$
Diet × Context	$F(1,29)=0.024$	$p=0.878$	partial $\eta^2=0.001$
Time × Diet × Context	$F(1.71,49.67)=0.631$	$p=0.512$	partial $\eta^2=0.021$

S27C Table

Females	Experiment 2 – kcal as % BW		
Diet	$F(1,30)=25.14$	**$p<0.001$**	partial $\eta^2=$**0.456**
Context	$F(1,30)=0.003$	$p=0.953$	partial $\eta^2=0.000$
Time	$F(3.40,102.1)=5.508$	**$p<0.001$**	partial $\eta^2=$**0.155**
Time × Diet	$F(3.40,102.1)=0.541$	$p=0.677$	partial $\eta^2=0.018$
Time × Context	$F(3.40,102.1)=1.330$	$p=0.267$	partial $\eta^2=0.042$
Diet × Context	$F(1,30)=0.428$	$p=0.518$	partial $\eta^2=0.014$
Time × Diet × Context	$F(3.40,102.1)=0.487$	$p=0.487$	partial $\eta^2=0.016$

S27D Table

Males	Experiment 2 – kcal as % BW		
Diet	$F(1,32)=0.793$	$p=0.380$	partial $\eta^2=0.024$
Context	$F(1,32)=0.424$	$p=0.520$	partial $\eta^2=0.013$
Time	$F(3.71,118.6)=6.216$	**$p<0.001$**	partial $\eta^2=$**0.163**
Time × Diet	$F(3.71,118.6)=1.178$	$p=0.324$	partial $\eta^2=0.036$
Time × Context	$F(3.71,118.6)=0.414$	$p=0.784$	partial $\eta^2=0.013$

Diet × Context	F(1,32)=2.189	p=0.149	partial η^2=0.064
Time × Diet × Context	F(3.71,118.6)=0.413	p=0.785	partial η^2=0.013

S27E Table

**Females**	**Experiment 3 – kcal as % BW**		
Diet	F(1,30)=6.312	**p=0.018**	partial η^2=**0.174**
Context	F(1,30)=0.005	p=0.947	partial η^2=0.000
Time	F(2.59,77.66)=5.137	**p=0.004**	partial η^2=**0.146**
Time × Diet	F(2.59,77.66)=1.132	p=0.337	partial η^2=0.036
Time × Context	F(2.59,77.66)=0.494	p=0.660	partial η^2=0.016
Diet × Context	F(1,30)=1.458	p=0.237	partial η^2=0.046
Time × Diet × Context	F(2.59,77.66)=0.217	p=0.858	partial η^2=0.007

S27F Table

**Males**	**Experiment 3 – kcal as % BW**		
Diet	F(1,28)=12.08	**p=0.002**	partial η^2=**0.301**
Context	F(1,28)=1.490	p=0.232	partial η^2=0.051
Time	F(3.06,85.77)=8.143	**p<0.001**	partial η^2=**0.225**
Time × Diet	F(3.06,85.77)=0.870	p=0.462	partial η^2=0.030
Time × Context	F(3.06,85.77)=1.053	p=0.374	partial η^2=0.036
Diet × Context	F(1,28)=0.277	p=0.603	partial η^2=0.010
Time × Diet × Context	F(3.06,85.77)=0.509	p=0.681	partial η^2=0.018

S19 Figure

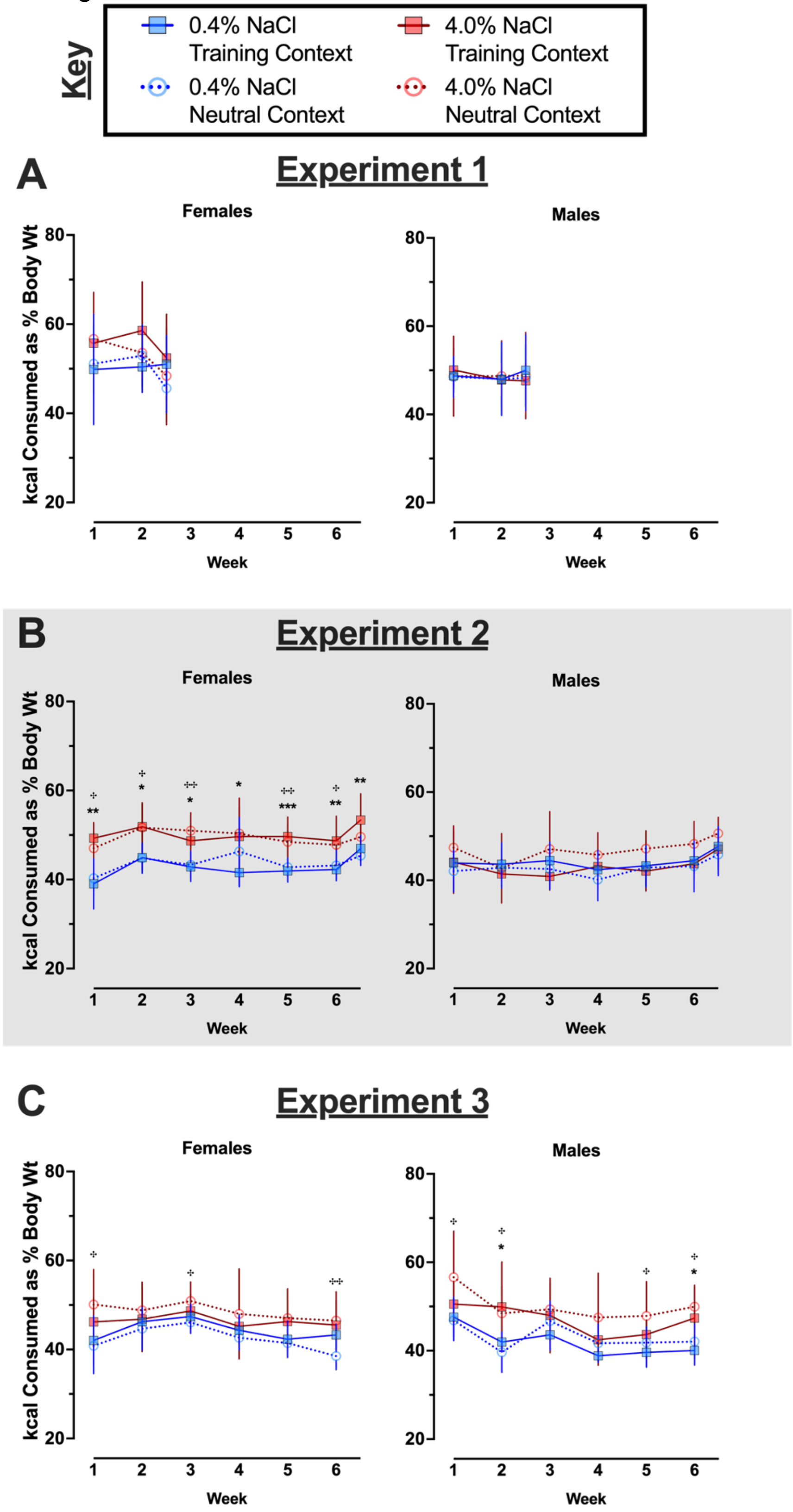
Key
0.4% NaCl Training Context
4.0% NaCl Training Context
0.4% NaCl Neutral Context
4.0% NaCl Neutral Context
A
Experiment 1
Females
Males
kcal Consumed as % Body Wt
Week
B
Experiment 2
Females
Males
kcal Consumed as % Body Wt
Week
C
Experiment 3
Females
Males
kcal Consumed as % Body Wt
Week

S19 Fig. Consumed kcal as a percentage of body weight by context fear conditioned mice across Experiments.

Mice assigned to 0.4% NaCl represented by blue symbols, mice assigned to 4.0% NaCl represented by red symbols; mice to be tested in Training Context represented by squares and solid lines, mice to be tested in Neutral Context represented by circles and dotted lines. Consumed kcal as a percentage of body weight was calculated for each full week, plus the partial weeks at the conclusion of A) Experiment 1 and B) Experiment 2 (grey shading). Some data loss occurred on the very last weighing day for a subset of animals in C) Experiment 3, thus graphs and repeated measures statistical analyses for Experiment 3 calculations cease at week 6 to maximize inclusion of mice in repeated measures analyses. Experiment 1: 0.4% NaCl females Training Context, n=8; 0.4% NaCl females Neutral Context, n=9; 4.0% NaCl females Training Context, n=8; 4.0% NaCl females Neutral Context, n=9; 0.4% NaCl males Training Context, n=8; 0.4% NaCl males Neutral Context, n=9; 4.0% NaCl males Training Context, n=8; 4.0% NaCl males Neutral Context, n=8. Experiment 2: 0.4% NaCl females Training Context, n=9; 0.4% NaCl females Neutral Context, n=9; 4.0% NaCl females Training Context, n=8; 4.0% NaCl females Neutral Context, n=8; 0.4% NaCl males Training Context, n=9; 0.4% NaCl males Neutral Context, n=9; 4.0% NaCl males Training Context, n=8; 4.0% NaCl males Neutral Context, n=10. Experiment 3: 0.4% NaCl females Training Context, n=8; 0.4% NaCl females Neutral Context, n=9; 4.0% NaCl females Training Context, n=9; 4.0% NaCl females Neutral Context, n=8; 0.4% NaCl males Training Context, n=8; 0.4% NaCl males Neutral Context, n=8; 4.0% NaCl males Training Context, n=8; 4.0% NaCl males Neutral Context, n=8. Data are graphed as mean ± 95% confidence interval. *$p<0.05$, **$p<0.01$, ***$p<0.001$ indicate difference between mice within the same sex consuming 0.4% NaCl versus 4.0% NaCl and tested in Training Context. $^{+}p<0.05$, $^{++}p<0.01$, $^{+++}p<0.001$ indicate difference

between mice within the same sex consuming 0.4% NaCl versus 4.0% NaCl and tested in Neutral Context.

S28 Table. Three-way repeated measures ANOVAs on weekly average NaCl consumed per day by control no shock mice across Experiments.

S28A Table

Experiment 1	NaCl/day		
Sex	$F(1,31)=6.870$	$p=0.013$	partial $\eta^2=0.181$
Diet	$F(1,31)=585.5$	$p<0.001$	partial $\eta^2=0.950$
Time	$F(1.55,47.89)=0.069$	$p=0.890$	partial $\eta^2=0.002$
Time × Sex	$F(1.55,47.89)=1.907$	$p=0.168$	partial $\eta^2=0.058$
Time × Diet	$F(1.55,47.89)=0.103$	$p=0.853$	partial $\eta^2=0.003$
Sex × Diet	$F(1,31)=4.262$	**$p=0.047$**	partial $\eta^2=$**0.121**
Time × Sex × Diet	$F(1.55,47.89)=1.733$	$p=0.193$	partial $\eta^2=0.053$

S28B Table

Experiment 2	NaCl/day		
Sex	$F(1,29)=14.80$	$p<0.001$	partial $\eta^2=0.338$
Diet	$F(1,29)=1821$	$p<0.001$	partial $\eta^2=0.984$
Time	$F(2.40,69.59)=3.232$	$p=0.037$	partial $\eta^2=0.100$
Time × Sex	$F(2.40,69.59)=0.157$	$p=0.889$	partial $\eta^2=0.005$
Time × Diet	$F(2.40,69.59)=3.330$	**$p=0.033$**	partial $\eta^2=$**0.103**
Sex × Diet	$F(1,29)=11.10$	**$p=0.002$**	partial $\eta^2=$**0.277**
Time × Sex × Diet	$F(2.40,69.59)=0.132$	$p=0.909$	partial $\eta^2=0.005$

S28C Table

Experiment 3	NaCl/day		
Sex	$F(1,28)=6.847$	$p=0.014$	partial $\eta^2=0.196$
Diet	$F(1,28)=318.3$	$p<0.001$	partial $\eta^2=0.919$
Time	$F(2.37,66.44)=0.759$	$p=0.493$	partial $\eta^2=0.026$
Time × Sex	$F(2.37,66.44)=1.633$	$p=0.199$	partial $\eta^2=0.055$
Time × Diet	$F(2.37,66.44)=0.729$	$p=0.508$	partial $\eta^2=0.025$
Sex × Diet	$F(1,28)=3.864$	**$p=0.059$**	partial $\eta^2=$**0.121**
Time × Sex × Diet	$F(2.37,66.44)=1.580$	$p=0.210$	partial $\eta^2=0.053$

S29 Table. Three-way repeated measures ANOVAs on weekly NaCl consumed as a percentage of body weight by control no shock mice across Experiments.

S29A Table

**Experiment 1**	**NaCl as % BW**		
Sex	F(1,31)=0.304	p=0.585	partial η^2=0.010
Diet	F(1,31)=323.9	p<0.001	partial η^2=0.913
Time	F(1.52,47.12)=0.936	p=0.376	partial η^2=0.029
Time × Sex	F(1.52,47.12)=3.195	p=0.063	partial η^2=0.093
Time × Diet	F(1.52,47.12)=0.743	p=0.447	partial η^2=0.023
Sex × Diet	F(1,31)=0.329	p=0.570	partial η^2=0.011
Time × Sex × Diet	F(1.52,47.12)=2.829	**p=0.083**	partial **η^2=0.084**

S29B Table

**Experiment 2**	**NaCl as % BW**		
Sex	F(1,29)=4.453	p=0.044	partial η^2=0.133
Diet	F(1,29)=2475	p<0.001	partial η^2=0.988
Time	F(1.92,55.69)=2.794	p=0.072	partial η^2=0.088
Time × Sex	F(1.92,55.69)=0.109	p=0.889	partial η^2=0.004
Time × Diet	F(1.92,55.69)=3.222	**p=0.049**	partial η^2=**0.100**
Sex × Diet	F(1,29)=3.125	**p=0.088**	partial η^2=**0.097**
Time × Sex × Diet	F(1.92,55.69)=0.074	p=0.923	partial η^2=0.003

S29C Table

**Experiment 3**	**NaCl as % BW**		
Sex	F(1,28)=0.211	p=0.649	partial η^2=0.007
Diet	F(1,28)=438.4	**p<0.001**	partial η^2=**0.940**
Time	F(2.91,81.55)=1.500	p=0.222	partial η^2=0.051
Time × Sex	F(2.91,81.55)=1.040	p=0.378	partial η^2=0.036
Time × Diet	F(2.91,81.55)=1.330	p=0.271	partial η^2=0.045
Sex × Diet	F(1,28)=0.521	p=0.476	partial η^2=0.018
Time × Sex × Diet	F(2.91,81.55)=0.940	p=0.423	partial η^2=0.032

S20 Figure

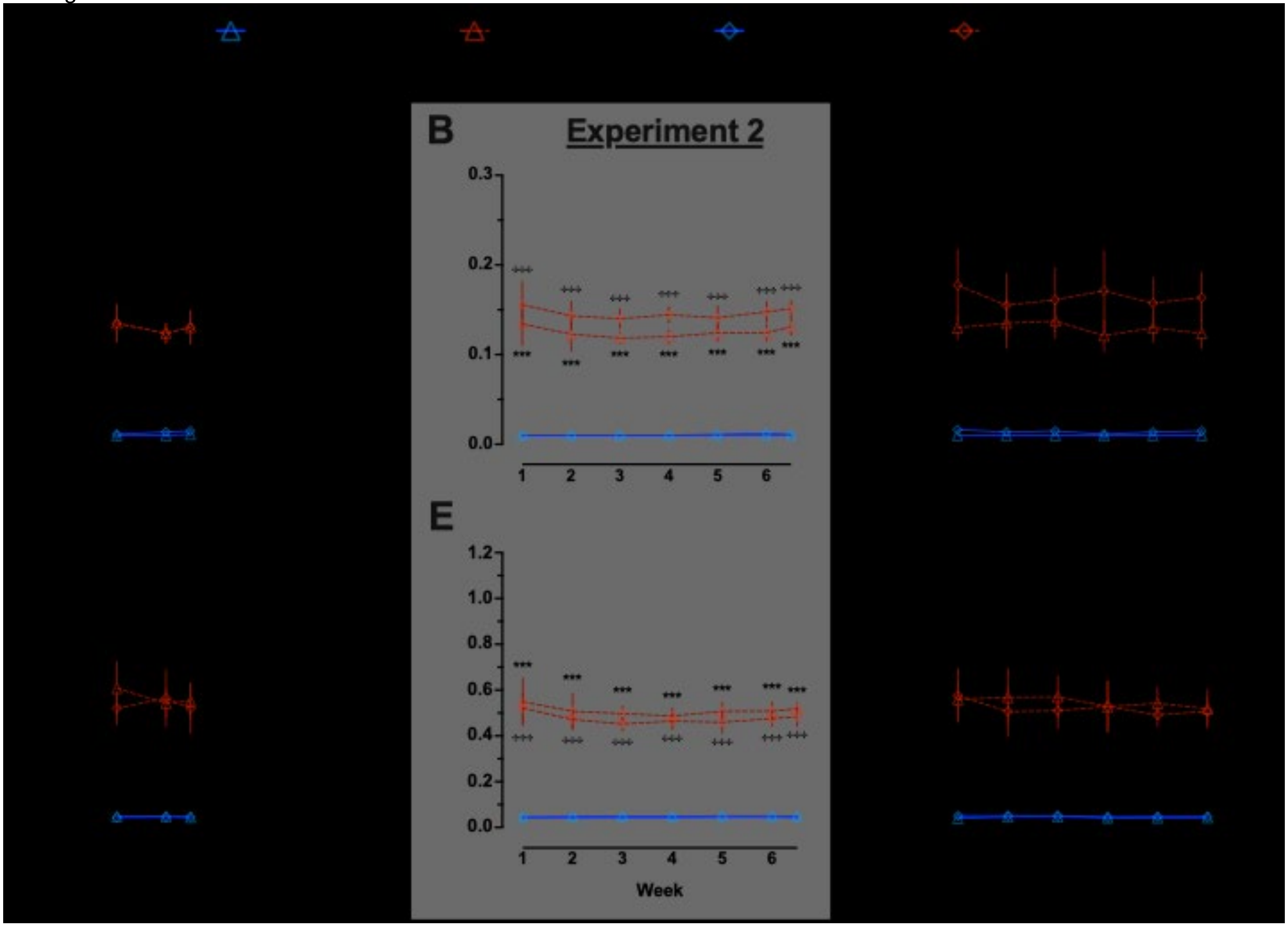

B
Experiment 2
0.3
0.2
0.1
0.0
1
2
3
4
5
6
E
1.2
1.0
0.8
0.6
0.4
0.2
0.0
1
2
3
4
5
6
Week

S20 Fig. Average NaCl consumed per day and NaCl consumed as a percentage of body weight by control no shock mice across Experiments.

Females represented by triangles, males by diamonds; 0.4% NaCl represented by blue symbols and solid lines, 4.0% NaCl represented by red symbols and dashed lines. A, B, C) NaCl consumed per day and D, E, F) NaCl consumption as a percentage of body weight were calculated for each full week, and for the partial week at the conclusion of A, D) Experiment 1 and B, E) Experiment 2 (grey shading). Some data loss occurred on the very last weighing day for a subset of animals in C, F) Experiment 3, thus graphs and repeated measures statistical analyses for Experiment 3 calculations cease at week 6 to maximize inclusion of mice in repeated measures analyses. Experiment 1: 0.4% NaCl females, n=9; 4.0% NaCl females, n=9; 0.4% NaCl males, n=8; 4.0% NaCl males, n=9. Experiment 2: 0.4% NaCl females, n=8; 4.0% NaCl females, n=7; 0.4% NaCl males, n=9; 4.0% NaCl males, n=9. Experiment 3: 0.4% NaCl females, n=8; 4.0% NaCl females, n=8; 0.4% NaCl males, n=8; 4.0% NaCl males, n=8. Data are graphed as mean ± 95% confidence interval. *p<0.05, **p<0.01, ***p<0.001 indicate difference between females consuming 0.4% NaCl versus 4.0% NaCl. $^{+}$p<0.05, $^{++}$p<0.01, $^{+++}$p<0.001 indicate difference between males consuming 0.4% NaCl versus 4.0% NaCl.

S30 Table. Three-way repeated measures ANOVAs on weekly water to NaCl ratio consumed by control no shock mice across Experiments.

S30A Table

**Experiment 1**	**Water:NaCl Ratio**		
Sex	$F(1,31)=13.68$	$p<0.001$	partial $\eta^2=0.306$
Diet	$F(1,31)=487.2$	$p<0.001$	partial $\eta^2=0.940$
Time	$F(1.39,43.09)=3.731$	**$p=0.047$**	partial $\eta^2=$**0.107**
Time × Sex	$F(1.39,43.09)=2.248$	$p=0.133$	partial $\eta^2=0.068$
Time × Diet	$F(1.39,43.09)=3.027$	$p=0.076$	partial $\eta^2=0.089$
Sex × Diet	$F(1,31)=18.22$	**$p<0.001$**	partial $\eta^2=$**0.370**
Time × Sex × Diet	$F(1.39,43.09)=2.048$	$p=0.154$	partial $\eta^2=0.062$

S30B Table

**Experiment 2**	**Water:NaCl Ratio**		
Sex	$F(1,29)=1.913$	$p=0.177$	partial $\eta^2=0.062$
Diet	$F(1,29)=589.6$	$p<0.001$	partial $\eta^2=0.953$
Time	$F(3.93,113.8)=2.126$	$p=0.083$	partial $\eta^2=0.068$
Time × Sex	$F(3.93,113.8)=5.567$	$p<0.001$	partial $\eta^2=0.161$
Time × Diet	$F(3.93,113.8)=1.161$	$p=0.332$	partial $\eta^2=0.038$
Sex × Diet	$F(1,29)=2.208$	$p=0.148$	partial $\eta^2=0.071$
Time × Sex × Diet	$F(3.93,113.8)=4.342$	**$p=0.003$**	partial $\eta^2=$**0.130**

S30C Table

**Experiment 3**	**Water:NaCl Ratio**		
Sex	$F(1,28)=2.707$	$p=0.111$	partial $\eta^2=0.088$
Diet	$F(1,28)=137.4$	$p<0.001$	partial $\eta^2=0.831$
Time	$F(2.85,79.82)=1.662$	$p=0.184$	partial $\eta^2=0.056$
Time × Sex	$F(2.85,79.82)=4.474$	$p=0.007$	partial $\eta^2=0.138$
Time × Diet	$F(2.85,79.82)=1.050$	$p=0.373$	partial $\eta^2=0.036$
Sex × Diet	$F(1,28)=0.347$	$p=0.561$	partial $\eta^2=0.012$
Time × Sex × Diet	$F(2.85,79.82)=4.428$	**$p=0.007$**	partial $\eta^2=$**0.137**

S21 Figure

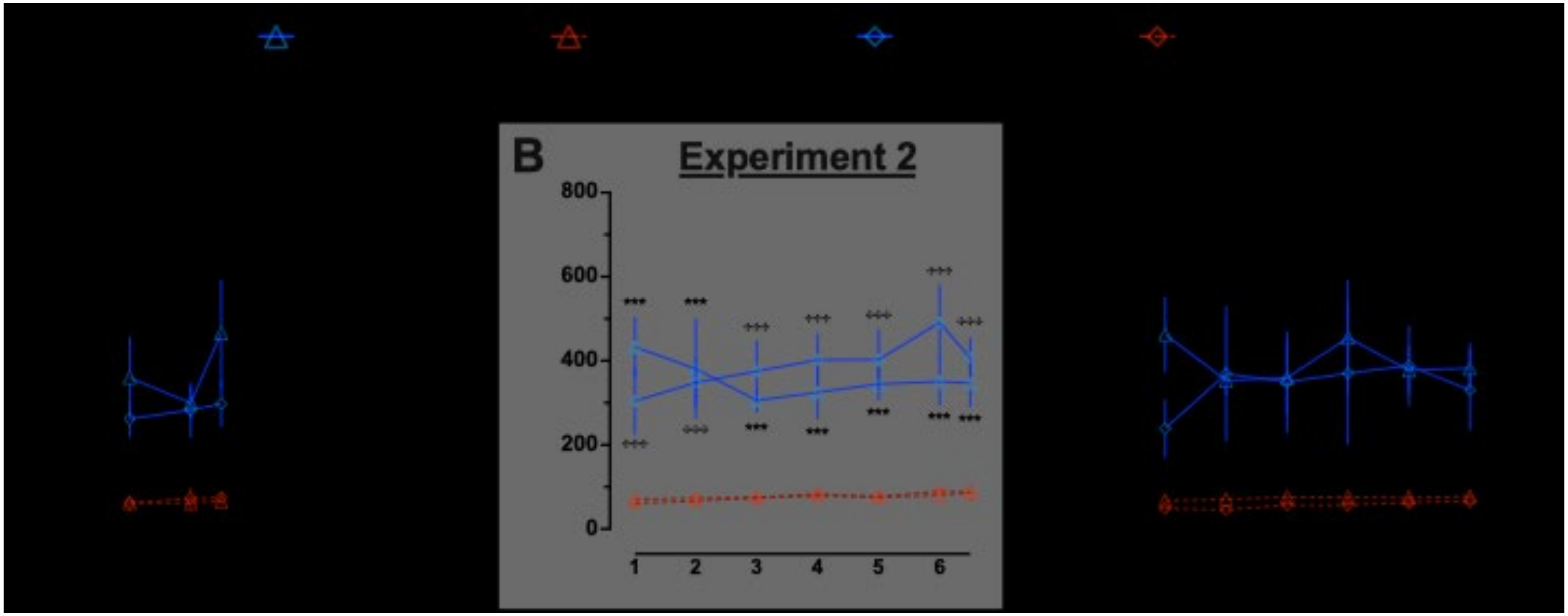

S21 Fig. Ratio of water to NaCl consumed by control no shock mice across Experiments. Females represented by triangles, males by diamonds; 0.4% NaCl represented by blue symbols and solid lines, 4.0% NaCl represented by red symbols and dashed lines. Water to NaCl consumption ratio was calculated for each full week, and for the partial week at the conclusion of A) Experiment 1 and B) Experiment 2 (grey shading). Some data loss occurred on the very last weighing day for a subset of animals in C) Experiment 3, thus graphs and repeated measures statistical analyses for Experiment 3 calculations cease at week 6 to maximize inclusion of mice in repeated measures analyses. Experiment 1: 0.4% NaCl females, n=9; 4.0% NaCl females, n=9; 0.4% NaCl males, n=8; 4.0% NaCl males, n=9. Experiment 2: 0.4% NaCl females, n=8; 4.0% NaCl females, n=7; 0.4% NaCl males, n=9; 4.0% NaCl males, n=9. Experiment 3: 0.4% NaCl females, n=8; 4.0% NaCl females, n=8; 0.4% NaCl males, n=8; 4.0% NaCl males, n=8. Data are graphed as mean ± 95% confidence interval. *$p<0.05$, **$p<0.01$, ***$p<0.001$ indicate difference between females consuming 0.4% NaCl versus 4.0% NaCl. $^{\ddagger}p<0.05$, $^{\ddagger\ddagger}p<0.01$, $^{\ddagger\ddagger\ddagger}p<0.001$ indicate difference between males consuming 0.4% NaCl versus 4.0% NaCl.

S31 Table. Three-way repeated measures ANOVAs on weekly average kcal consumed per day by control no shock mice across Experiments.

S31A Table

Experiment 1	kcal/day		
Sex	F(1,31)=17.84	**p<0.001**	partial η^2=**0.365**
Diet	F(1,31)=4.655	**p=0.039**	partial η^2=**0.131**
Time	F(1.55,48.15)=0.003	p=0.990	partial η^2=0.000
Time × Sex	F(1.55,48.15)=2.408	p=0.113	partial η^2=0.072
Time × Diet	F(1.55,48.15)=0.225	p=0.743	partial η^2=0.007
Sex × Diet	F(1,31)=0.193	p=0.663	partial η^2=0.006
Time × Sex × Diet	F(1.55,48.15)=1.253	p=0.288	partial η^2=0.039

S31B Table

Experiment 2	kcal/day		
Sex	F(1,29)=20.20	**p<0.001**	partial η^2=**0.411**
Diet	F(1,29)=6.500	p=0.016	partial η^2=0.183
Time	F(3.19,92.45)=3.755	p=0.012	partial η^2=0.115
Time × Sex	F(3.19,92.45)=0.524	p=0.678	partial η^2=0.018
Time × Diet	F(3.19,92.45)=4.385	**p=0.005**	partial η^2=**0.131**
Sex × Diet	F(1,29)=0.433	p=0.516	partial η^2=0.015
Time × Sex × Diet	F(3.19,92.45)=0.359	p=0.795	partial η^2=0.012

S31C Table

Experiment 3	kcal/day		
Sex	F(1,28)=24.96	**p<0.001**	partial η^2=**0.471**
Diet	F(1,28)=5.135	**p=0.031**	partial η^2=**0.155**
Time	F(2.77,77.66)=1.733	p=0.171	partial η^2=0.058
Time × Sex	F(2.77,77.66)=1.512	p=0.220	partial η^2=0.051
Time × Diet	F(2.77,77.66)=1.539	p=0.214	partial η^2=0.052
Sex × Diet	F(1,28)=0.916	p=0.347	partial η^2=0.032
Time × Sex × Diet	F(2.77,77.66)=1.162	p=0.328	partial η^2=0.040

S32 Table. Three-way repeated measures ANOVAs on weekly average kcal consumed as a percentage of body weight by control no shock mice across Experiments.

S32A Table

Experiment 1	Kcal as % BW
Sex	$F(1,31)=0.151$ $p=0.700$ partial $\eta^2=0.005$
Diet	$F(1,31)=3.279$ $p=0.080$ partial $\eta^2=0.096$
Time	$F(1.65,51.04)=1.353$ $p=0.265$ partial $\eta^2=0.042$
Time × Sex	$F(1.65,51.04)=3.592$ $\mathbf{p=0.043}$ partial $\eta^2=\mathbf{0.104}$
Time × Diet	$F(1.65,51.04)=0.089$ $p=0.880$ partial $\eta^2=0.003$
Sex × Diet	$F(1,31)=0.348$ $p=0.560$ partial $\eta^2=0.011$
Time × Sex × Diet	$F(1.65,51.04)=1.194$ $p=0.304$ partial $\eta^2=0.037$

S32B Table

Experiment 2	Kcal as % BW
Sex	$F(1,29)=7.328$ $\mathbf{p=0.011}$ partial $\eta^2=\mathbf{0.202}$
Diet	$F(1,29)=1.017$ $p=0.322$ partial $\eta^2=0.034$
Time	$F(2.59,75.20)=1.345$ $p=0.267$ partial $\eta^2=0.044$
Time × Sex	$F(2.59,75.20)=0.667$ $p=0.554$ partial $\eta^2=0.022$
Time × Diet	$F(2.59,75.20)=4.208$ $\mathbf{p=0.011}$ partial $\eta^2=\mathbf{0.127}$
Sex × Diet	$F(1,29)=0.014$ $p=0.906$ partial $\eta^2=0.000$
Time × Sex × Diet	$F(2.59,75.20)=0.430$ $p=0.704$ partial $\eta^2=0.015$

S32C Table

Experiment 3	Kcal as % BW
Sex	$F(1,28)=0.431$ $p=0.517$ partial $\eta^2=0.015$
Diet	$F(1,28)=4.291$ $\mathbf{p=0.048}$ partial $\eta^2=\mathbf{0.133}$
Time	$F(3.02,84.44)=3.121$ $\mathbf{p=0.030}$ partial $\eta^2=\mathbf{0.100}$
Time × Sex	$F(3.02,84.44)=1.336$ $p=0.268$ partial $\eta^2=0.046$
Time × Diet	$F(3.02,84.44)=1.945$ $p=0.128$ partial $\eta^2=0.065$
Sex × Diet	$F(1,28)=2.776$ $p=0.107$ partial $\eta^2=0.090$
Time × Sex × Diet	$F(3.02,84.44)=0.648$ $p=0.587$ partial $\eta^2=0.023$

S22 Figure

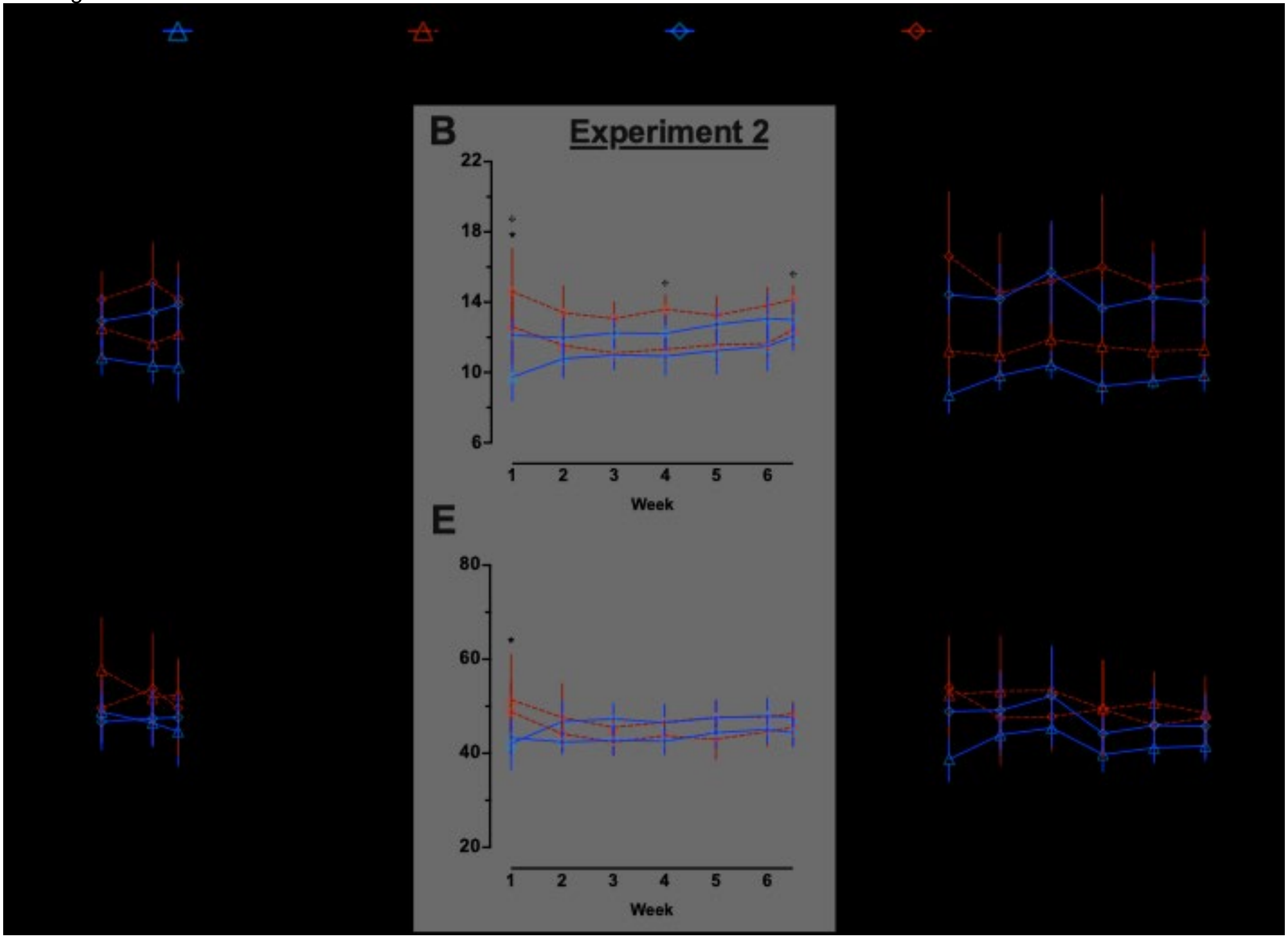
B
Experiment 2
22
18
14
10
6
1 2 3 4 5 6
Week
E
80
60
40
20
1 2 3 4 5 6
Week

S22 Fig. Average kcal consumed per day and kcal consumed as a percentage of body weight by control no shock mice across Experiments.

Females represented by triangles, males by diamonds; 0.4% NaCl represented by blue symbols and solid lines, 4.0% NaCl represented by red symbols and dashed lines. A, B, C) Consumption of kcal per day and D, E, F) kcal consumption as a percentage of body weight were calculated for each full week, and for the partial week at the conclusion of A, D) Experiment 1 and B, E) Experiment 2 (grey shading). Some data loss occurred on the very last weighing day for a subset of animals in C, F) Experiment 3, thus graphs and repeated measures statistical analyses for Experiment 3 calculations cease at week 6 to maximize inclusion of mice in repeated measures analyses. Experiment 1: 0.4% NaCl females, n=9; 4.0% NaCl females, n=9; 0.4% NaCl males, n=8; 4.0% NaCl males, n=9. Experiment 2: 0.4% NaCl females, n=8; 4.0% NaCl females, n=7; 0.4% NaCl males, n=9; 4.0% NaCl males, n=9. Experiment 3: 0.4% NaCl females, n=8; 4.0% NaCl females, n=8; 0.4% NaCl males, n=8; 4.0% NaCl males, n=8. Data are graphed as mean ± 95% confidence interval. *p<0.05, **p<0.01, ***p<0.001 indicate difference between females consuming 0.4% NaCl versus 4.0% NaCl. $^{\ddagger}p<0.05$, $^{\ddagger\ddagger}p<0.01$, $^{\ddagger\ddagger\ddagger}p<0.001$ indicate difference between males consuming 0.4% NaCl versus 4.0% NaCl.

S23 Figure

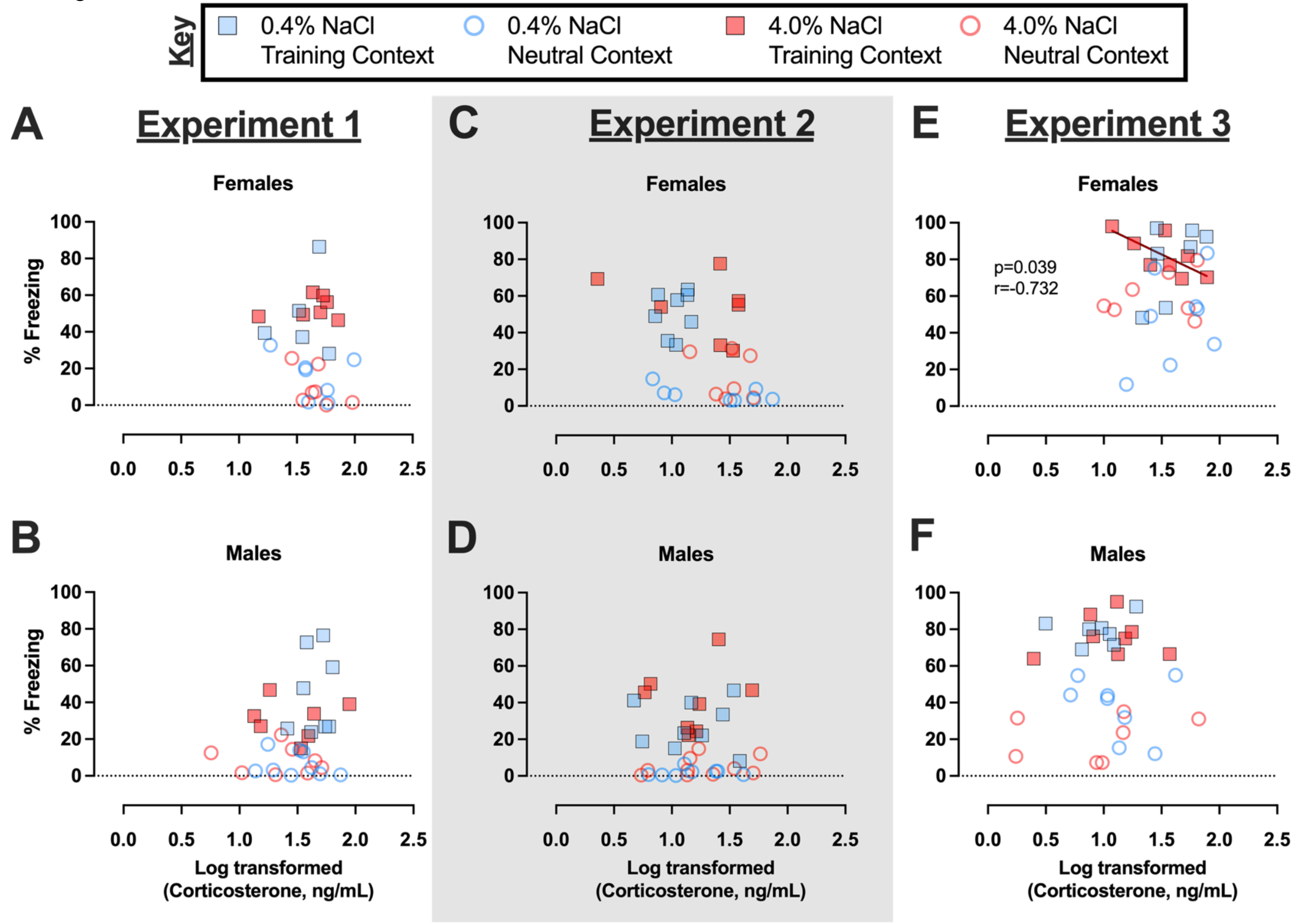

Key
0.4% NaCl Training Context
0.4% NaCl Neutral Context
4.0% NaCl Training Context
4.0% NaCl Neutral Context
A
Experiment 1
Females
% Freezing
B
Males
C
Experiment 2
D
E
Experiment 3
p=0.039
r=-0.732
F
Log transformed (Corticosterone, ng/mL)

S23 Fig. Correlations between context fear expression and log-transformed serum corticosterone levels across contexts and Experiments.

Mice assigned to 0.4% NaCl represented by blue symbols, mice assigned to 4.0% NaCl represented by red symbols; mice tested in Training Context represented by squares, mice tested in Neutral Context represented by circles. Individual log-transformed serum corticosterone levels were plotted on the x-axis against the same individual's contextual fear expression during minutes two through six of the 10 min testing session plotted on the y-axis. Data for each sex were graphed for A, B) Experiment 1, C, D) Experiment 2 (grey shading), and E, F) Experiment 3. Significant correlation indicated with solid line (female). Experiment 1: 0.4% NaCl females Training Context, n=5; 0.4% NaCl females Neutral Context, n=7; 4.0% NaCl females Training Context, n=7; 4.0% NaCl females Neutral Context, n=7; 0.4% NaCl males Training Context, n=8; 0.4% NaCl males Neutral Context, n=9; 4.0% NaCl males Training Context, n=7; 4.0% NaCl males Neutral Context, n=8. Experiment 2: 0.4% NaCl females Training Context, n=8; 0.4% NaCl females Neutral Context, n=8; 4.0% NaCl females Training Context, n=7; 4.0% NaCl females Neutral Context, n=7; 0.4% NaCl males Training Context, n=9; 0.4% NaCl males Neutral Context, n=8; 4.0% NaCl males Training Context, n=8; 4.0% NaCl males Neutral Context, n=10. Experiment 3: 0.4% NaCl females Training Context, n=7; 0.4% NaCl females Neutral Context, n=8; 4.0% NaCl females Training Context, n=8; 4.0% NaCl females Neutral Context, n=7; 0.4% NaCl males Training Context, n=7; 0.4% NaCl males Neutral Context, n=8; 4.0% NaCl males Training Context, n=8; 4.0% NaCl males Neutral Context, n=7.

S24 Figure

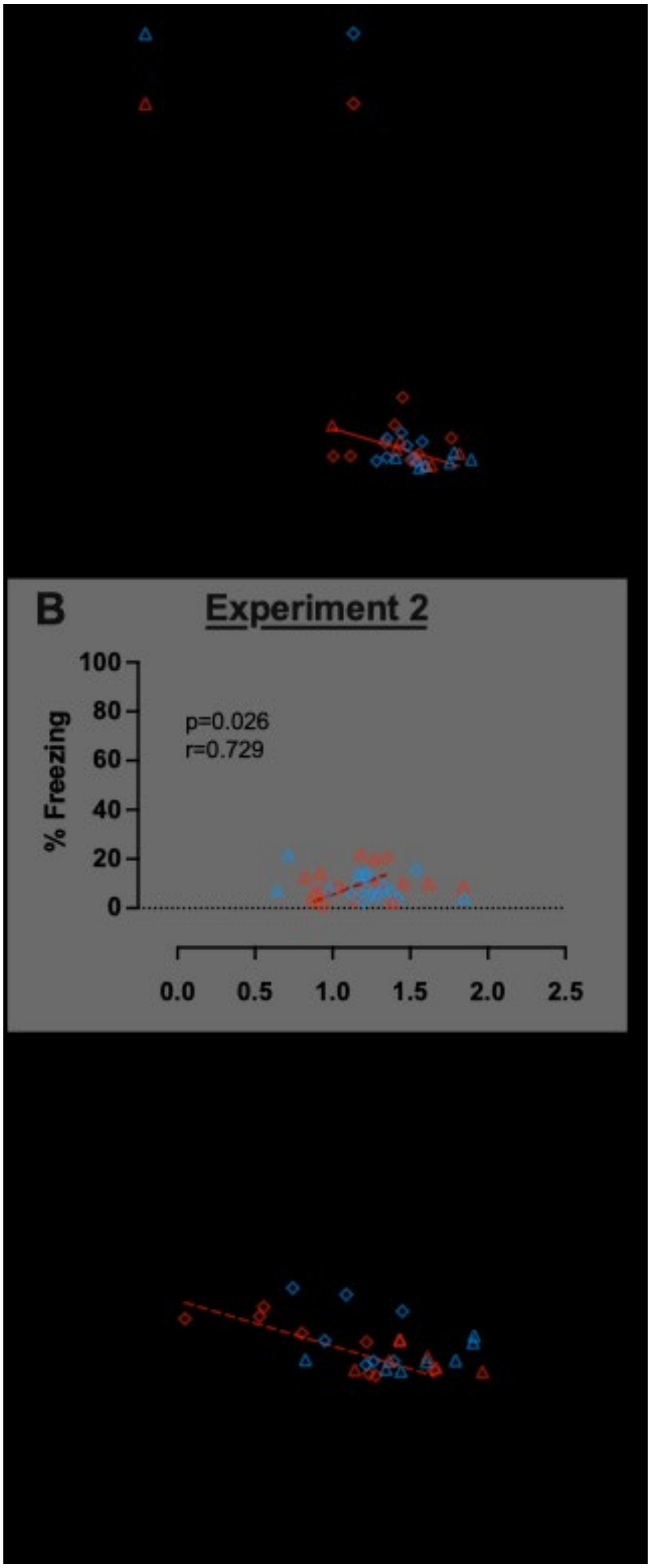
B
Experiment 2
% Freezing
100
80
60
40
20
0
p=0.026
r=0.729
0.0
0.5
1.0
1.5
2.0
2.5

S24 Figure. Correlations between context fear expression and log-transformed serum corticosterone levels in control no shock mice across Experiments.

Females represented by triangles, males by diamonds; 0.4% NaCl represented by blue symbols, 4.0% NaCl represented by red symbols. Individual log-transformed serum corticosterone levels were plotted on the x-axis against the same individual's contextual fear expression during minutes two through six of the 10 min testing session plotted on the y-axis. Data for each sex of control no shock mice were graphed for A) Experiment 1, B) Experiment 2 (grey shading), and C) Experiment 3. Significant correlations indicated with solid (female) or dashed (male) lines. Experiment 1: 0.4% NaCl females, n=6; 4.0% NaCl females, n=7; 0.4% NaCl males, n=8; 4.0% NaCl males, n=8. Experiment 2: 0.4% NaCl females, n=7; 4.0% NaCl females, n=8; 0.4% NaCl males, n=9; 4.0% NaCl males, n=9. Experiment 3: 0.4% NaCl females, n=7; 4.0% NaCl females, n=7; 0.4% NaCl males, n=7; 4.0% NaCl males, n=8.

S33 Table. Details of confirmed outliers (> 5 standard deviations (SD) ± average of data set sans suspected outlier) within respective data sets. Outliers are marked with red font.

S33A Table

Male • Experiment 3 • 0.4%NaCl<br>No Shock • Training Context • Training baseline			
**Animal ID**	**Data**	**Outlier Determination**	
0445	6.36		
0447	8.93		
0514	24.01	>6 SD beyond mean	
0519	0.88	2.66	*Avg*
0523	0.69	3.54	*SD*
0529	0.00	23.88	*Avg + 6 SD*
0697	0.00		
0796	1.75		

S33B Table

Male • Experiment 1 • 4.0%NaCl<br>No Shock • Training Context • Testing			
**Animal ID**	**Data**	**Outlier Determination**	
0331	30.21		
0345	18.41		
0352	75.65	>7 SD beyond mean	
0377	3.63		
0388	10.92	11.56	*Avg*
0391	6.03	9.04	*SD*
0409	5.15	74.81	*Avg + 7 SD*
0415	12.92		
0422	5.24		

S33C Table

Male • Experiment 1 • 4.0%NaCl<br>Shock • Training Context • Testing		
**Animal ID**	**Data**	**Outlier Determination**
0342	86.01	>5 SD beyond mean

0344	39.07	30.84	*Avg*
0357	33.78	10.65	*SD*
0367	21.75	84.09	*Avg + 5 SD*
0387	14.97		
0413	32.57		
0416	46.73		
0423	27.04		

S33D Table

Female • Experiment 2 • 0.4%NaCl<br>Shock • Neutral Context • Testing			
**Animal ID**	**Data**	**Outlier Determination**	
0608	3.56		
0610	9.26		
0613	3.16		
0622	6.15		
0626	3.12	6.35	*Avg*
0667	3.71	4.05	*SD*
0677	7.10	30.63	*Avg + 6 SD*
0678	14.73		
0682	32.69	>6 SD beyond mean	

S33E Table

Male • Experiment 2 • 0.4%NaCl<br>Shock • Neutral Context • Testing			
**Animal ID**	**Data**	**Outlier Determination**	
0445	2.31		
0447	2.58		
0514	0.58	2.04	*Avg*
0519	6.52	2.05	*SD*
0523	0.76	26.61	*Avg + 12 SD*
0529	2.57		
0697	27.97	>12 SD beyond mean	
0796	0.27		
0807	0.75		

S33F Table

Male・Experiment 3・0.4%NaCl<br>Shock・Training Context・Testing			
**Animal ID**	**Data**	**Outlier Determination**	
0073	69.01	79.18	*Avg*
0187	80.66	7.74	*SD*
0278	83.16	40.46	*Avg - 5 SD*
0312	80.07		
0324	77.38		
0428	92.45		
0434	38.13	>5 SD beyond mean	
0436	71.55		

S33G Table

Male・Experiment 3・4.0%NaCl<br>Osmolality			
**Animal ID**	**Data**	**Outlier Determination**	
0077	338	>7 SD beyond mean	
0188	315		
0306	316		
0311	322	316.29	*Avg*
0322	314	2.87	*SD*
0427	318	336.38	*Avg + 7 SD*
0435	314		
0438	315		

S33H Table

Female・Experiment 1・0.4%NaCl<br>No Shock・Training Context・Corticosterone Log			
**Animal ID**	**Data**	**Outlier Determination**	
0394	1.67		
0531	1.84		
0539	1.99		
0544	1.64	1.75	*Avg*
0550	1.48	0.19	*SD*
0556	1.87	0.82	*Avg - 5 SD*
0642	0.79	>5 SD beyond mean	

S33I Table

Female・Experiment 2・0.4%NaCl
Shock・Training Context・Corticosterone Log

Animal ID	Data	Outlier Determination	
0616	1.13		
0618	1.94	>7 SD beyond mean	
0620	1.17		
0625	0.86	1.03	*Avg*
0628	0.88	0.12	*SD*
0676	1.05	1.86	*Avg + 7 SD*
0681	1.14		
0683	1.04		
0771	0.96		

S33J Table

Male・Experiment 1・0.4%NaCl・Shock
Training Context・Water:NaCl Ratio Wk 1

Animal ID	Data	Outlier Determination	
0341	333.63		
0353	153.57		
0354	441.7	>6 SD beyond mean	
0368	229.87		
0389	218.90	231.82	*Avg*
0407	218.37	34.28	*SD*
0417	257.84	437.51	*Avg + 6 SD*
0419	210.53		

S33K Table

Male・Experiment 1・0.4%NaCl・Shock
Neutral Context・Water:NaCl Ratio Wk 2

Animal ID	Data	Outlier Determination	
0333	349.81	346.46	*Avg*
0343	342.23	84.92	*SD*
0350	301.87	1110.78	*Avg + 9 SD*
0358	262.82		
0376	383.72		
0390	486.90		
0408	1127.25	>5 SD beyond mean	
0418	224.22		

0421	420.07

S33L Table

Male・Experiment 1・0.4%NaCl・Shock<br>Neutral Context・Water:NaCl Ratio Wk 3			
**Animal ID**	**Data**	**Outlier Determination**	
0333	302.97	275.09	*Avg*
0343	258.75	47.54	*SD*
0350	272.09	1225.95	*Avg + 20 SD*
0358	188.51		
0376	320.86		
0390	258.38		
0408	1228.7	>20 SD beyond mean	
0418	256.58		
0421	342.59		

S33M Table

Male・Experiment 1・4.0%NaCl・Shock<br>Neutral Context・Water:NaCl Ratio Wk 2.5			
**Animal ID**	**Data**	**Outlier Determination**	
0329	75.44	64.79	*Avg*
0332	59.85	9.38	*SD*
0340	65.41	139.82	*Avg + 8 SD*
0351	140.65	>8 SD beyond mean	
0371	75.72		
0411	68.95		
0412	57.52		
0420	50.65		

S33N Table

Female・Experiment 2・0.4%NaCl・Shock<br>Training Context・Water:NaCl Ratio Wk 4		
**Animal ID**	**Data**	**Outlier Determination**
0616	375.63	
0618	900.00	>5 SD beyond mean
0620	433.96	
0625	309.68	

0628	376.56	424.32	*Avg*
0676	474.04	86.14	*SD*
0681	377.40	855.01	*Avg + 5 SD*
0683	454.14		
0771	593.11		

S33O Table

Male・Experiment 2・0.4%NaCl・Shock Training Context・Water:NaCl Ratio Wk 2			
**Animal ID**	**Data**	**Outlier Determination**	
0451	310.26	329.64	*Avg*
0453	364.10	81.02	*SD*
0802	243.72	815.77	*Avg + 6 SD*
0512	257.58		
0516	234.38		
0520	459.49		
0526	381.22		
0702	386.36		
0795	848.89	>6 SD beyond mean	

S33P Table

Female・Experiment 3・0.4%NaCl・Shock Neutral Context・Water:NaCl Ratio Wk 2			
**Animal ID**	**Data**	**Outlier Determination**	
0559	777.14	>5 SD beyond mean	
0568	443.72	329.34	*Avg*
0570	416.26	74.83	*SD*
0589	245.56	703.51	*Avg + 5 SD*
0591	257.72		
0651	270.49		
0656	341.22		
0663	367.35		
0664	292.40		

S33Q Table

Female・Experiment 3・4.0%NaCl・Shock Neutral Context・Water:NaCl Ratio Wk 4

Animal ID	Data	Outlier Determination	
0561	75.48	75.50	*Avg*
0563	80.97	7.02	*SD*
0567	70.87	110.58	*Avg + 5 SD*
0573	111.94	>5 SD beyond mean	
0586	76.37		
0660	87.56		
0662	67.32		
0665	69.94		
