# Supplementary material for: Generalization of contextual fear is sex-specifically affected by high salt intake
Source: PLoS One. 2023 Jul 13;18(7):e0286221. doi: 10.1371/journal.pone.0286221 (PMC10343085; doi:10.1371/journal.pone.0286221)
Supplement: S28 Table — (PDF) [file pone.0286221.s028.pdf]

Supplemental Material for

Generalization of contextual fear is sex-specifically affected by high salt intake

Jasmin N. Beaver[1,2], Brady L. Weber[1,2], Matthew T. Ford[1], Anna E. Anello[1,2], Kaden M. Ruffin[1], Sarah K. Kassis[1,2], T. Lee Gilman[1,2,3]*

[1]Department of Psychological Sciences, Kent State University, Kent, Ohio, United States of America

[2]Brain Health Research Institute, Kent State University, Kent, Ohio, United States of America

[3]Healthy Communities Research Institute, Kent State University, Kent, Ohio, United States of America

*Corresponding Author

Email: lgilman1@kent.edu (TLG)

S28 Table. Three-way repeated measures ANOVAs on weekly average NaCl consumed per day by control no shock mice across Experiments.

S28A Table

Experiment 1	NaCl/day		
Sex	$F(1,31)=6.870$	$p=0.013$	partial $\eta^2=0.181$
Diet	$F(1,31)=585.5$	$p<0.001$	partial $\eta^2=0.950$
Time	$F(1.55,47.89)=0.069$	$p=0.890$	partial $\eta^2=0.002$
Time × Sex	$F(1.55,47.89)=1.907$	$p=0.168$	partial $\eta^2=0.058$
Time × Diet	$F(1.55,47.89)=0.103$	$p=0.853$	partial $\eta^2=0.003$
Sex × Diet	$F(1,31)=4.262$	**$p=0.047$**	partial $\eta^2=$**0.121**
Time × Sex × Diet	$F(1.55,47.89)=1.733$	$p=0.193$	partial $\eta^2=0.053$

S28B Table

Experiment 2	NaCl/day		
Sex	$F(1,29)=14.80$	$p<0.001$	partial $\eta^2=0.338$
Diet	$F(1,29)=1821$	$p<0.001$	partial $\eta^2=0.984$
Time	$F(2.40,69.59)=3.232$	$p=0.037$	partial $\eta^2=0.100$
Time × Sex	$F(2.40,69.59)=0.157$	$p=0.889$	partial $\eta^2=0.005$
Time × Diet	$F(2.40,69.59)=3.330$	**$p=0.033$**	partial $\eta^2=$**0.103**
Sex × Diet	$F(1,29)=11.10$	**$p=0.002$**	partial $\eta^2=$**0.277**
Time × Sex × Diet	$F(2.40,69.59)=0.132$	$p=0.909$	partial $\eta^2=0.005$

S28C Table

Experiment 3	NaCl/day		
Sex	$F(1,28)=6.847$	$p=0.014$	partial $\eta^2=0.196$
Diet	$F(1,28)=318.3$	$p<0.001$	partial $\eta^2=0.919$
Time	$F(2.37,66.44)=0.759$	$p=0.493$	partial $\eta^2=0.026$
Time × Sex	$F(2.37,66.44)=1.633$	$p=0.199$	partial $\eta^2=0.055$
Time × Diet	$F(2.37,66.44)=0.729$	$p=0.508$	partial $\eta^2=0.025$
Sex × Diet	$F(1,28)=3.864$	**$p=0.059$**	partial $\eta^2=$**0.121**
Time × Sex × Diet	$F(2.37,66.44)=1.580$	$p=0.210$	partial $\eta^2=0.053$
