# Supplementary material for: Generalization of contextual fear is sex-specifically affected by high salt intake
Source: PLoS One. 2023 Jul 13;18(7):e0286221. doi: 10.1371/journal.pone.0286221 (PMC10343085; doi:10.1371/journal.pone.0286221)
Supplement: S29 Table — (PDF) [file pone.0286221.s029.pdf]

Supplemental Material for

Generalization of contextual fear is sex-specifically affected by high salt intake

Jasmin N. Beaver[1,2], Brady L. Weber[1,2], Matthew T. Ford[1], Anna E. Anello[1,2], Kaden M. Ruffin[1], Sarah K. Kassis[1,2], T. Lee Gilman[1,2,3]*

[1]Department of Psychological Sciences, Kent State University, Kent, Ohio, United States of America

[2]Brain Health Research Institute, Kent State University, Kent, Ohio, United States of America

[3]Healthy Communities Research Institute, Kent State University, Kent, Ohio, United States of America

*Corresponding Author

Email: lgilman1@kent.edu (TLG)

S29 Table. Three-way repeated measures ANOVAs on weekly NaCl consumed as a percentage of body weight by control no shock mice across Experiments.

S29A Table

**Experiment 1**	**NaCl as % BW**
Sex	F(1,31)=0.304 p=0.585 partial η^2=0.010
Diet	F(1,31)=323.9 p<0.001 partial η^2=0.913
Time	F(1.52,47.12)=0.936 p=0.376 partial η^2=0.029
Time × Sex	F(1.52,47.12)=3.195 p=0.063 partial η^2=0.093
Time × Diet	F(1.52,47.12)=0.743 p=0.447 partial η^2=0.023
Sex × Diet	F(1,31)=0.329 p=0.570 partial η^2=0.011
Time × Sex × Diet	F(1.52,47.12)=2.829 **p=0.083** partial **η^2=0.084**

S29B Table

**Experiment 2**	**NaCl as % BW**
Sex	F(1,29)=4.453 p=0.044 partial η^2=0.133
Diet	F(1,29)=2475 p<0.001 partial η^2=0.988
Time	F(1.92,55.69)=2.794 p=0.072 partial η^2=0.088
Time × Sex	F(1.92,55.69)=0.109 p=0.889 partial η^2=0.004
Time × Diet	F(1.92,55.69)=3.222 **p=0.049** partial η^2=**0.100**
Sex × Diet	F(1,29)=3.125 **p=0.088** partial η^2=**0.097**
Time × Sex × Diet	F(1.92,55.69)=0.074 p=0.923 partial η^2=0.003

S29C Table

**Experiment 3**	**NaCl as % BW**
Sex	F(1,28)=0.211 p=0.649 partial η^2=0.007
Diet	F(1,28)=438.4 **p<0.001** partial η^2=**0.940**
Time	F(2.91,81.55)=1.500 p=0.222 partial η^2=0.051
Time × Sex	F(2.91,81.55)=1.040 p=0.378 partial η^2=0.036
Time × Diet	F(2.91,81.55)=1.330 p=0.271 partial η^2=0.045
Sex × Diet	F(1,28)=0.521 p=0.476 partial η^2=0.018
Time × Sex × Diet	F(2.91,81.55)=0.940 p=0.423 partial η^2=0.032
