# Supplementary material for: Generalization of contextual fear is sex-specifically affected by high salt intake
Source: PLoS One. 2023 Jul 13;18(7):e0286221. doi: 10.1371/journal.pone.0286221 (PMC10343085; doi:10.1371/journal.pone.0286221)
Supplement: S30 Table — (PDF) [file pone.0286221.s030.pdf]

Supplemental Material for

Generalization of contextual fear is sex-specifically affected by high salt intake

Jasmin N. Beaver[1,2], Brady L. Weber[1,2], Matthew T. Ford[1], Anna E. Anello[1,2], Kaden M. Ruffin[1], Sarah K. Kassis[1,2], T. Lee Gilman[1,2,3]*

[1]Department of Psychological Sciences, Kent State University, Kent, Ohio, United States of America

[2]Brain Health Research Institute, Kent State University, Kent, Ohio, United States of America

[3]Healthy Communities Research Institute, Kent State University, Kent, Ohio, United States of America

*Corresponding Author

Email: lgilman1@kent.edu (TLG)

S30 Table. Three-way repeated measures ANOVAs on weekly water to NaCl ratio consumed by control no shock mice across Experiments.

S30A Table

**Experiment 1**	**Water:NaCl Ratio**		
Sex	$F(1,31)=13.68$	$p<0.001$	partial $\eta^2=0.306$
Diet	$F(1,31)=487.2$	$p<0.001$	partial $\eta^2=0.940$
Time	$F(1.39,43.09)=3.731$	**$p=0.047$**	partial $\eta^2=$**0.107**
Time × Sex	$F(1.39,43.09)=2.248$	$p=0.133$	partial $\eta^2=0.068$
Time × Diet	$F(1.39,43.09)=3.027$	$p=0.076$	partial $\eta^2=0.089$
Sex × Diet	$F(1,31)=18.22$	**$p<0.001$**	partial $\eta^2=$**0.370**
Time × Sex × Diet	$F(1.39,43.09)=2.048$	$p=0.154$	partial $\eta^2=0.062$

S30B Table

**Experiment 2**	**Water:NaCl Ratio**		
Sex	$F(1,29)=1.913$	$p=0.177$	partial $\eta^2=0.062$
Diet	$F(1,29)=589.6$	$p<0.001$	partial $\eta^2=0.953$
Time	$F(3.93,113.8)=2.126$	$p=0.083$	partial $\eta^2=0.068$
Time × Sex	$F(3.93,113.8)=5.567$	$p<0.001$	partial $\eta^2=0.161$
Time × Diet	$F(3.93,113.8)=1.161$	$p=0.332$	partial $\eta^2=0.038$
Sex × Diet	$F(1,29)=2.208$	$p=0.148$	partial $\eta^2=0.071$
Time × Sex × Diet	$F(3.93,113.8)=4.342$	**$p=0.003$**	partial $\eta^2=$**0.130**

S30C Table

**Experiment 3**	**Water:NaCl Ratio**		
Sex	$F(1,28)=2.707$	$p=0.111$	partial $\eta^2=0.088$
Diet	$F(1,28)=137.4$	$p<0.001$	partial $\eta^2=0.831$
Time	$F(2.85,79.82)=1.662$	$p=0.184$	partial $\eta^2=0.056$
Time × Sex	$F(2.85,79.82)=4.474$	$p=0.007$	partial $\eta^2=0.138$
Time × Diet	$F(2.85,79.82)=1.050$	$p=0.373$	partial $\eta^2=0.036$
Sex × Diet	$F(1,28)=0.347$	$p=0.561$	partial $\eta^2=0.012$
Time × Sex × Diet	$F(2.85,79.82)=4.428$	**$p=0.007$**	partial $\eta^2=$**0.137**
