# Supplementary material for: Generalization of contextual fear is sex-specifically affected by high salt intake
Source: PLoS One. 2023 Jul 13;18(7):e0286221. doi: 10.1371/journal.pone.0286221 (PMC10343085; doi:10.1371/journal.pone.0286221)
Supplement: S31 Table — (PDF) [file pone.0286221.s031.pdf]

Supplemental Material for

Generalization of contextual fear is sex-specifically affected by high salt intake

Jasmin N. Beaver[1,2], Brady L. Weber[1,2], Matthew T. Ford[1], Anna E. Anello[1,2], Kaden M. Ruffin[1], Sarah K. Kassis[1,2], T. Lee Gilman[1,2,3]*

[1]Department of Psychological Sciences, Kent State University, Kent, Ohio, United States of America

[2]Brain Health Research Institute, Kent State University, Kent, Ohio, United States of America

[3]Healthy Communities Research Institute, Kent State University, Kent, Ohio, United States of America

*Corresponding Author

Email: lgilman1@kent.edu (TLG)

S31 Table. Three-way repeated measures ANOVAs on weekly average kcal consumed per day by control no shock mice across Experiments.

S31A Table

Experiment 1	kcal/day		
Sex	$F(1,31)=17.84$	**$p<0.001$**	partial η^2=**0.365**
Diet	$F(1,31)=4.655$	**$p=0.039$**	partial η^2=**0.131**
Time	$F(1.55,48.15)=0.003$	$p=0.990$	partial η^2=0.000
Time × Sex	$F(1.55,48.15)=2.408$	$p=0.113$	partial η^2=0.072
Time × Diet	$F(1.55,48.15)=0.225$	$p=0.743$	partial η^2=0.007
Sex × Diet	$F(1,31)=0.193$	$p=0.663$	partial η^2=0.006
Time × Sex × Diet	$F(1.55,48.15)=1.253$	$p=0.288$	partial η^2=0.039

S31B Table

Experiment 2	kcal/day		
Sex	$F(1,29)=20.20$	**$p<0.001$**	partial η^2=**0.411**
Diet	$F(1,29)=6.500$	$p=0.016$	partial η^2=0.183
Time	$F(3.19,92.45)=3.755$	$p=0.012$	partial η^2=0.115
Time × Sex	$F(3.19,92.45)=0.524$	$p=0.678$	partial η^2=0.018
Time × Diet	$F(3.19,92.45)=4.385$	**$p=0.005$**	partial η^2=**0.131**
Sex × Diet	$F(1,29)=0.433$	$p=0.516$	partial η^2=0.015
Time × Sex × Diet	$F(3.19,92.45)=0.359$	$p=0.795$	partial η^2=0.012

S31C Table

Experiment 3	kcal/day		
Sex	$F(1,28)=24.96$	**$p<0.001$**	partial η^2=**0.471**
Diet	$F(1,28)=5.135$	**$p=0.031$**	partial η^2=**0.155**
Time	$F(2.77,77.66)=1.733$	$p=0.171$	partial η^2=0.058
Time × Sex	$F(2.77,77.66)=1.512$	$p=0.220$	partial η^2=0.051
Time × Diet	$F(2.77,77.66)=1.539$	$p=0.214$	partial η^2=0.052
Sex × Diet	$F(1,28)=0.916$	$p=0.347$	partial η^2=0.032
Time × Sex × Diet	$F(2.77,77.66)=1.162$	$p=0.328$	partial η^2=0.040
