# Supplementary material for: Generalization of contextual fear is sex-specifically affected by high salt intake
Source: PLoS One. 2023 Jul 13;18(7):e0286221. doi: 10.1371/journal.pone.0286221 (PMC10343085; doi:10.1371/journal.pone.0286221)
Supplement: S32 Table — (PDF) [file pone.0286221.s032.pdf]

Supplemental Material for

Generalization of contextual fear is sex-specifically affected by high salt intake

Jasmin N. Beaver[1,2], Brady L. Weber[1,2], Matthew T. Ford[1], Anna E. Anello[1,2], Kaden M. Ruffin[1], Sarah K. Kassis[1,2], T. Lee Gilman[1,2,3]*

[1]Department of Psychological Sciences, Kent State University, Kent, Ohio, United States of America

[2]Brain Health Research Institute, Kent State University, Kent, Ohio, United States of America

[3]Healthy Communities Research Institute, Kent State University, Kent, Ohio, United States of America

*Corresponding Author

Email: lgilman1@kent.edu (TLG)

S32 Table. Three-way repeated measures ANOVAs on weekly average kcal consumed as a percentage of body weight by control no shock mice across Experiments.

S32A Table

Experiment 1	Kcal as % BW
Sex	$F(1,31)=0.151$ $p=0.700$ partial $\eta^2=0.005$
Diet	$F(1,31)=3.279$ $p=0.080$ partial $\eta^2=0.096$
Time	$F(1.65,51.04)=1.353$ $p=0.265$ partial $\eta^2=0.042$
Time × Sex	$F(1.65,51.04)=3.592$ $\mathbf{p=0.043}$ partial $\eta^2=\mathbf{0.104}$
Time × Diet	$F(1.65,51.04)=0.089$ $p=0.880$ partial $\eta^2=0.003$
Sex × Diet	$F(1,31)=0.348$ $p=0.560$ partial $\eta^2=0.011$
Time × Sex × Diet	$F(1.65,51.04)=1.194$ $p=0.304$ partial $\eta^2=0.037$

S32B Table

Experiment 2	Kcal as % BW
Sex	$F(1,29)=7.328$ $\mathbf{p=0.011}$ partial $\eta^2=\mathbf{0.202}$
Diet	$F(1,29)=1.017$ $p=0.322$ partial $\eta^2=0.034$
Time	$F(2.59,75.20)=1.345$ $p=0.267$ partial $\eta^2=0.044$
Time × Sex	$F(2.59,75.20)=0.667$ $p=0.554$ partial $\eta^2=0.022$
Time × Diet	$F(2.59,75.20)=4.208$ $\mathbf{p=0.011}$ partial $\eta^2=\mathbf{0.127}$
Sex × Diet	$F(1,29)=0.014$ $p=0.906$ partial $\eta^2=0.000$
Time × Sex × Diet	$F(2.59,75.20)=0.430$ $p=0.704$ partial $\eta^2=0.015$

S32C Table

Experiment 3	Kcal as % BW
Sex	$F(1,28)=0.431$ $p=0.517$ partial $\eta^2=0.015$
Diet	$F(1,28)=4.291$ $\mathbf{p=0.048}$ partial $\eta^2=\mathbf{0.133}$
Time	$F(3.02,84.44)=3.121$ $\mathbf{p=0.030}$ partial $\eta^2=\mathbf{0.100}$
Time × Sex	$F(3.02,84.44)=1.336$ $p=0.268$ partial $\eta^2=0.046$
Time × Diet	$F(3.02,84.44)=1.945$ $p=0.128$ partial $\eta^2=0.065$
Sex × Diet	$F(1,28)=2.776$ $p=0.107$ partial $\eta^2=0.090$
Time × Sex × Diet	$F(3.02,84.44)=0.648$ $p=0.587$ partial $\eta^2=0.023$
