# Supplementary material for: Generalization of contextual fear is sex-specifically affected by high salt intake
Source: PLoS One. 2023 Jul 13;18(7):e0286221. doi: 10.1371/journal.pone.0286221 (PMC10343085; doi:10.1371/journal.pone.0286221)
Supplement: S33 Table — Outliers are marked with red font. (PDF) [file pone.0286221.s033.pdf]

Supplemental Material for

Generalization of contextual fear is sex-specifically affected by high salt intake

Jasmin N. Beaver[1,2], Brady L. Weber[1,2], Matthew T. Ford[1], Anna E. Anello[1,2], Kaden M. Ruffin[1], Sarah K. Kassis[1,2], T. Lee Gilman[1,2,3]*

[1]Department of Psychological Sciences, Kent State University, Kent, Ohio, United States of America

[2]Brain Health Research Institute, Kent State University, Kent, Ohio, United States of America

[3]Healthy Communities Research Institute, Kent State University, Kent, Ohio, United States of America

*Corresponding Author

Email: lgilman1@kent.edu (TLG)

S33 Table. Details of confirmed outliers (> 5 standard deviations (SD) ± average of data set sans suspected outlier) within respective data sets. Outliers are marked with red font.

S33A Table

Male・Experiment 3・0.4%NaCl<br>No Shock・Training Context・Training baseline			
**Animal ID**	**Data**	**Outlier Determination**	
0445	6.36		
0447	8.93		
0514	24.01	>6 SD beyond mean	
0519	0.88	2.66	*Avg*
0523	0.69	3.54	*SD*
0529	0.00	23.88	*Avg + 6 SD*
0697	0.00		
0796	1.75		

S33B Table

Male・Experiment 1・4.0%NaCl<br>No Shock・Training Context・Testing			
**Animal ID**	**Data**	**Outlier Determination**	
0331	30.21		
0345	18.41		
0352	75.65	>7 SD beyond mean	
0377	3.63		
0388	10.92	11.56	*Avg*
0391	6.03	9.04	*SD*
0409	5.15	74.81	*Avg + 7 SD*
0415	12.92		
0422	5.24		

S33C Table

Male・Experiment 1・4.0%NaCl<br>Shock・Training Context・Testing		
**Animal ID**	**Data**	**Outlier Determination**
0342	86.01	>5 SD beyond mean

0344	39.07	30.84	*Avg*
0357	33.78	10.65	*SD*
0367	21.75	84.09	*Avg + 5 SD*
0387	14.97		
0413	32.57		
0416	46.73		
0423	27.04		

S33D Table

Female · Experiment 2 · 0.4%NaCl<br>Shock · Neutral Context · Testing			
**Animal ID**	**Data**	**Outlier Determination**	
0608	3.56		
0610	9.26		
0613	3.16		
0622	6.15		
0626	3.12	6.35	*Avg*
0667	3.71	4.05	*SD*
0677	7.10	30.63	*Avg + 6 SD*
0678	14.73		
0682	32.69	>6 SD beyond mean	

S33E Table

Male · Experiment 2 · 0.4%NaCl<br>Shock · Neutral Context · Testing			
**Animal ID**	**Data**	**Outlier Determination**	
0445	2.31		
0447	2.58		
0514	0.58	2.04	*Avg*
0519	6.52	2.05	*SD*
0523	0.76	26.61	*Avg + 12 SD*
0529	2.57		
0697	27.97	>12 SD beyond mean	
0796	0.27		
0807	0.75		

S33F Table

Male・Experiment 3・0.4%NaCl<br>Shock・Training Context・Testing			
**Animal ID**	**Data**	**Outlier Determination**	
0073	69.01	79.18	*Avg*
0187	80.66	7.74	*SD*
0278	83.16	40.46	*Avg - 5 SD*
0312	80.07		
0324	77.38		
0428	92.45		
0434	38.13	>5 SD beyond mean	
0436	71.55		

S33G Table

Male・Experiment 3・4.0%NaCl<br>Osmolality			
**Animal ID**	**Data**	**Outlier Determination**	
0077	338	>7 SD beyond mean	
0188	315		
0306	316		
0311	322	316.29	*Avg*
0322	314	2.87	*SD*
0427	318	336.38	*Avg + 7 SD*
0435	314		
0438	315		

S33H Table

Female・Experiment 1・0.4%NaCl<br>No Shock・Training Context・Corticosterone Log			
**Animal ID**	**Data**	**Outlier Determination**	
0394	1.67		
0531	1.84		
0539	1.99		
0544	1.64	1.75	*Avg*
0550	1.48	0.19	*SD*
0556	1.87	0.82	*Avg - 5 SD*
0642	0.79	>5 SD beyond mean	

S33I Table

Female・Experiment 2・0.4%NaCl Shock・Training Context・Corticosterone Log			
**Animal ID**	**Data**	**Outlier Determination**	
0616	1.13		
0618	1.94	>7 SD beyond mean	
0620	1.17		
0625	0.86	1.03	*Avg*
0628	0.88	0.12	*SD*
0676	1.05	1.86	*Avg + 7 SD*
0681	1.14		
0683	1.04		
0771	0.96		

S33J Table

Male・Experiment 1・0.4%NaCl・Shock Training Context・Water:NaCl Ratio Wk 1			
**Animal ID**	**Data**	**Outlier Determination**	
0341	333.63		
0353	153.57		
0354	441.7	>6 SD beyond mean	
0368	229.87		
0389	218.90	231.82	*Avg*
0407	218.37	34.28	*SD*
0417	257.84	437.51	*Avg + 6 SD*
0419	210.53		

S33K Table

Male・Experiment 1・0.4%NaCl・Shock Neutral Context・Water:NaCl Ratio Wk 2			
**Animal ID**	**Data**	**Outlier Determination**	
0333	349.81	346.46	*Avg*
0343	342.23	84.92	*SD*
0350	301.87	1110.78	*Avg + 9 SD*
0358	262.82		
0376	383.72		
0390	486.90		
0408	1127.25	>5 SD beyond mean	
0418	224.22		

0421	420.07

S33L Table

Male・Experiment 1・0.4%NaCl・Shock Neutral Context・Water:NaCl Ratio Wk 3			
**Animal ID**	**Data**	**Outlier Determination**	
0333	302.97	275.09	*Avg*
0343	258.75	47.54	*SD*
0350	272.09	1225.95	*Avg + 20 SD*
0358	188.51		
0376	320.86		
0390	258.38		
0408	1228.7	>20 SD beyond mean	
0418	256.58		
0421	342.59		

S33M Table

Male・Experiment 1・4.0%NaCl・Shock Neutral Context・Water:NaCl Ratio Wk 2.5			
**Animal ID**	**Data**	**Outlier Determination**	
0329	75.44	64.79	*Avg*
0332	59.85	9.38	*SD*
0340	65.41	139.82	*Avg + 8 SD*
0351	140.65	>8 SD beyond mean	
0371	75.72		
0411	68.95		
0412	57.52		
0420	50.65		

S33N Table

Female・Experiment 2・0.4%NaCl・Shock Training Context・Water:NaCl Ratio Wk 4			
**Animal ID**	**Data**	**Outlier Determination**	
0616	375.63		
0618	900.00	>5 SD beyond mean	
0620	433.96		
0625	309.68		

0628	376.56	424.32	*Avg*
0676	474.04	86.14	*SD*
0681	377.40	855.01	*Avg + 5 SD*
0683	454.14		
0771	593.11		

S33O Table

Male • Experiment 2 • 0.4%NaCl • Shock Training Context • Water:NaCl Ratio Wk 2			
**Animal ID**	**Data**	**Outlier Determination**	
0451	310.26	329.64	*Avg*
0453	364.10	81.02	*SD*
0802	243.72	815.77	*Avg + 6 SD*
0512	257.58		
0516	234.38		
0520	459.49		
0526	381.22		
0702	386.36		
0795	848.89	>6 SD beyond mean	

S33P Table

Female • Experiment 3 • 0.4%NaCl • Shock Neutral Context • Water:NaCl Ratio Wk 2			
**Animal ID**	**Data**	**Outlier Determination**	
0559	777.14	>5 SD beyond mean	
0568	443.72	329.34	*Avg*
0570	416.26	74.83	*SD*
0589	245.56	703.51	*Avg + 5 SD*
0591	257.72		
0651	270.49		
0656	341.22		
0663	367.35		
0664	292.40		

S33Q Table

Female • Experiment 3 • 4.0%NaCl • Shock Neutral Context • Water:NaCl Ratio Wk 4

Animal ID	Data	Outlier Determination	
0561	75.48	75.50	*Avg*
0563	80.97	7.02	*SD*
0567	70.87	110.58	*Avg + 5 SD*
0573	111.94	>5 SD beyond mean	
0586	76.37		
0660	87.56		
0662	67.32		
0665	69.94		
