# Supplementary material for: Generalization of contextual fear is sex-specifically affected by high salt intake
Source: PLoS One. 2023 Jul 13;18(7):e0286221. doi: 10.1371/journal.pone.0286221 (PMC10343085; doi:10.1371/journal.pone.0286221)
Supplement: S1 Fig — (PDF) [file pone.0286221.s034.pdf]

Supplemental Material for

Generalization of contextual fear is sex-specifically affected by high salt intake

Jasmin N. Beaver[1,2], Brady L. Weber[1,2], Matthew T. Ford[1], Anna E. Anello[1,2], Kaden M. Ruffin[1], Sarah K. Kassis[1,2], T. Lee Gilman[1,2,3]*

[1]Department of Psychological Sciences, Kent State University, Kent, Ohio, United States of America

[2]Brain Health Research Institute, Kent State University, Kent, Ohio, United States of America

[3]Healthy Communities Research Institute, Kent State University, Kent, Ohio, United States of America

*Corresponding Author

Email: lgilman1@kent.edu (TLG)

S1 Figure

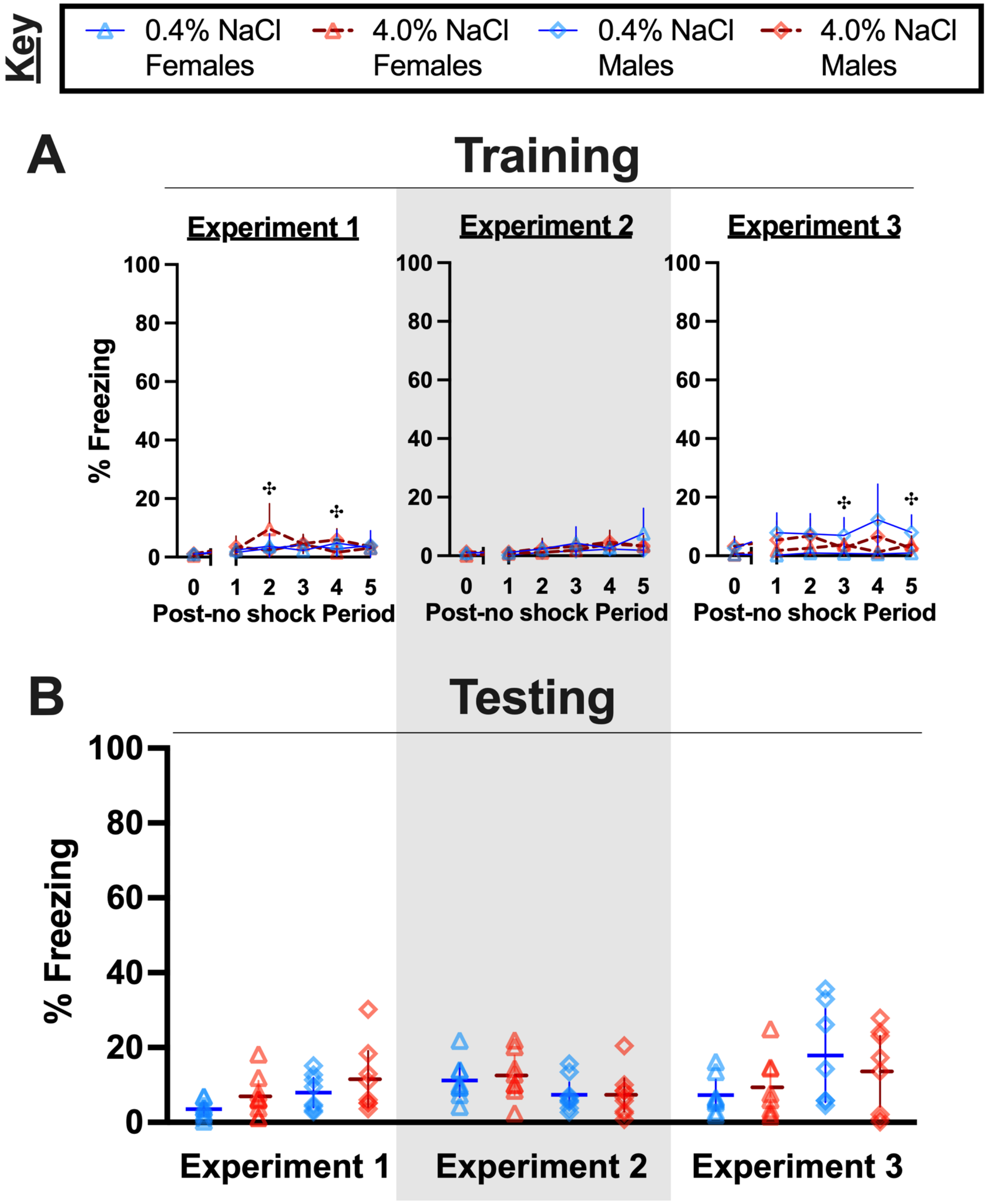
Key
0.4% NaCl Females
4.0% NaCl Females
0.4% NaCl Males
4.0% NaCl Males
A
Training
Experiment 1
Experiment 2
Experiment 3
% Freezing
100
80
60
40
20
0
0 1 2 3 4 5
Post-no shock Period
B
Testing
% Freezing
Experiment 1
Experiment 2
Experiment 3

S1 Fig. Fear behavior in no shock control mice across Experiments.

Females represented by triangles, males by diamonds; 0.4% NaCl represented by blue symbols and solid lines, 4.0% NaCl represented by red symbols and dashed lines. A) Percent freezing during 'training' of control mice exposed to the Training Context, but receiving no foot shocks for the duration of the 6 min 'training' session. B) Percent freezing during testing of control no shock mice exposed to the Training Context for minutes 2 through 6 of a 10 min testing session. Experiment 1: 0.4% NaCl females, n=9; 4.0% NaCl females, n=9; 0.4% NaCl males, n=8; 4.0% NaCl males, n=8. Experiment 2 (grey shading): 0.4% NaCl females, n=8; 4.0% NaCl females, n=8; 0.4% NaCl males, n=9; 4.0% NaCl males, n=9. Experiment 3: 0.4% NaCl females, n=8; 4.0% NaCl females, n=8; 0.4% NaCl males, n=7; 4.0% NaCl males, n=8. Data are graphed as mean ± 95% confidence interval. ‡indicates $p<0.05$ difference between males consuming 0.4% and 4.0% NaCl diets at indicated time points.
