# Supplementary material for: Generalization of contextual fear is sex-specifically affected by high salt intake
Source: PLoS One. 2023 Jul 13;18(7):e0286221. doi: 10.1371/journal.pone.0286221 (PMC10343085; doi:10.1371/journal.pone.0286221)
Supplement: S3 Fig — (PDF) [file pone.0286221.s036.pdf]

**Supplemental Material for**

# Generalization of contextual fear is sex-specifically affected by high salt intake

Jasmin N. Beaver[1,2], Brady L. Weber[1,2], Matthew T. Ford[1], Anna E. Anello[1,2], Kaden M. Ruffin[1], Sarah K. Kassis[1,2], T. Lee Gilman[1,2,3]*

[1]Department of Psychological Sciences, Kent State University, Kent, Ohio, United States of America

[2]Brain Health Research Institute, Kent State University, Kent, Ohio, United States of America

[3]Healthy Communities Research Institute, Kent State University, Kent, Ohio, United States of America

*Corresponding Author

Email: lgilman1@kent.edu (TLG)

S3 Figure

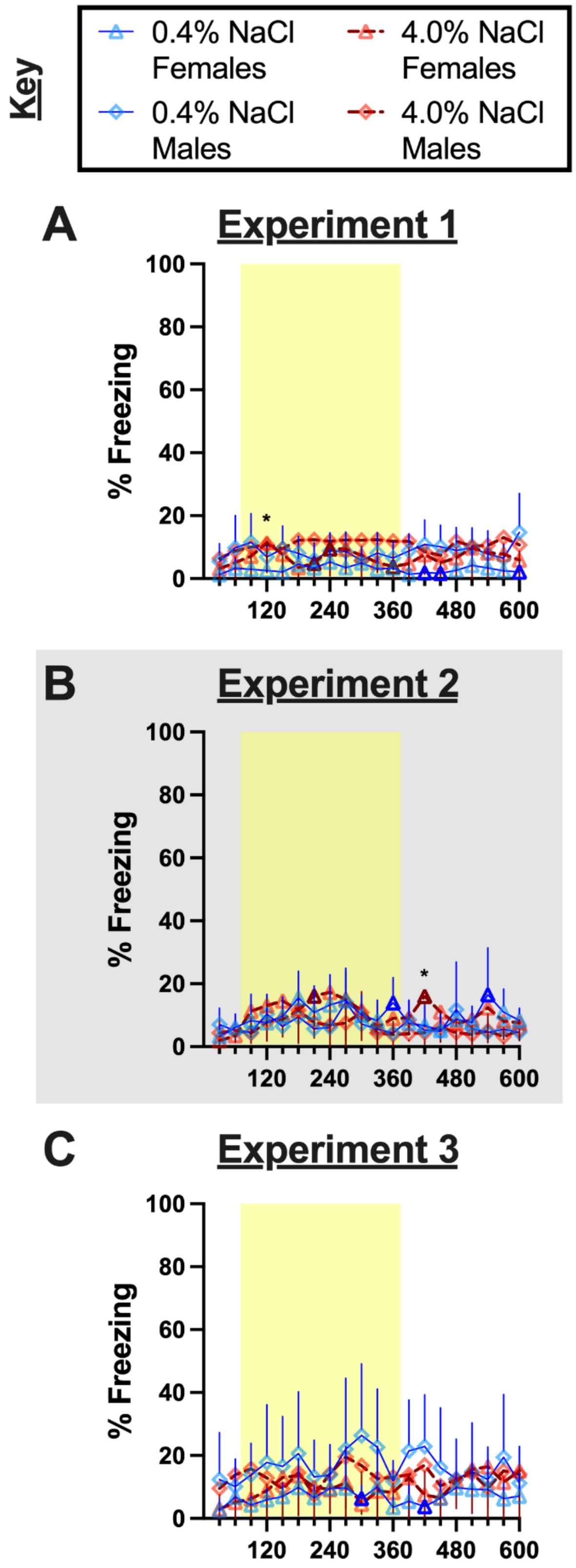

Key
0.4% NaCl Females
4.0% NaCl Females
0.4% NaCl Males
4.0% NaCl Males
A
Experiment 1
B
Experiment 2
C
Experiment 3
% Freezing
100
80
60
40
20
0
120
240
360
480
600
*

**S3 Fig. Time course of context fear expression testing in no shock groups**. Females represented by triangles, males by diamonds; 0.4% NaCl represented by blue symbols and solid lines, 4.0% NaCl represented by red symbols and dashed lines. Percent freezing for each 30 s bin of the 10 min testing session is graphed for all mice. Yellow shading indicates minutes 2 through 6, which were averaged and analyzed as our original measure of context fear expression. Testing occurred A) 48 h after training in Experiment 1, during which mice underwent two weeks of diet manipulation; B) 48 h after training in Experiment 2 (grey shading), during which mice underwent six weeks of diet manipulation; and C) four weeks after training in Experiment 3, during which mice underwent six total weeks of diet manipulation (training occurred after two weeks of diet manipulation). Experiment 1: 0.4% NaCl females, n=9; 4.0% NaCl females, n=9; 0.4% NaCl males, n=8; 4.0% NaCl males, n=8. Experiment 2 (grey shading): 0.4% NaCl females, n=8; 4.0% NaCl females, n=8; 0.4% NaCl males, n=9; 4.0% NaCl males, n=9. Experiment 3: 0.4% NaCl females, n=8; 4.0% NaCl females, n=8; 0.4% NaCl males, n=7; 4.0% NaCl males, n=8. Data are graphed as mean ± 95% confidence interval; pairwise comparisons were made using Bonferroni correction. Thick symbol borders on female data indicate significant ($p<0.05$) differences between females and males for that specific diet. *$p<0.05$ indicates significant differences between females on 0.4% versus 4.0% NaCl.
