# Supplementary material for: Generalization of contextual fear is sex-specifically affected by high salt intake
Source: PLoS One. 2023 Jul 13;18(7):e0286221. doi: 10.1371/journal.pone.0286221 (PMC10343085; doi:10.1371/journal.pone.0286221)
Supplement: S4 Fig — (PDF) [file pone.0286221.s037.pdf]

Supplemental Material for

Generalization of contextual fear is sex-specifically affected by high salt intake

Jasmin N. Beaver[1,2], Brady L. Weber[1,2], Matthew T. Ford[1], Anna E. Anello[1,2], Kaden M. Ruffin[1], Sarah K. Kassis[1,2], T. Lee Gilman[1,2,3]*

[1]Department of Psychological Sciences, Kent State University, Kent, Ohio, United States of America

[2]Brain Health Research Institute, Kent State University, Kent, Ohio, United States of America

[3]Healthy Communities Research Institute, Kent State University, Kent, Ohio, United States of America

*Corresponding Author

Email: lgilman1@kent.edu (TLG)

S4 Figure

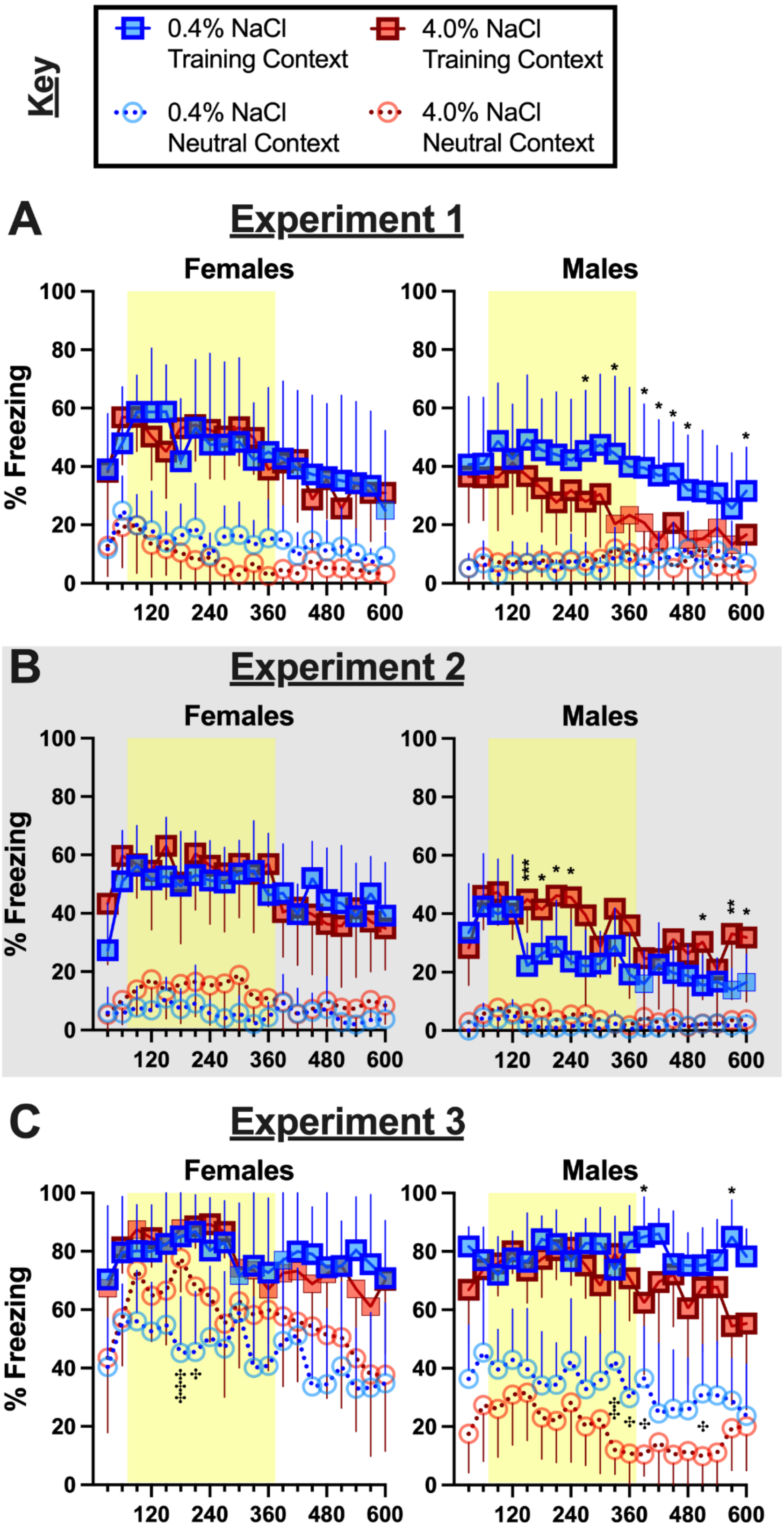
Key
0.4% NaCl Training Context
4.0% NaCl Training Context
0.4% NaCl Neutral Context
4.0% NaCl Neutral Context
A
Experiment 1
Females
Males
% Freezing
100
80
60
40
20
0
120 240 360 480 600
B
Experiment 2
Females
Males
% Freezing
C
Experiment 3
Females
Males
% Freezing

S4 Fig. Time course of context fear expression testing in shock groups. Mice assigned to 0.4% NaCl represented by blue symbols, mice assigned to 4.0% NaCl represented by red symbols; mice tested in Training Context represented by squares and solid lines, mice tested in Neutral Context represented by circles and dotted lines. Percent freezing for each 30 s bin of the 10 min testing session is graphed for all mice. Yellow shading indicates minutes 2 through 6, which were averaged and analyzed as our original measure of context fear expression. Testing occurred A) 48 h after training in Experiment 1, during which mice underwent two weeks of diet manipulation; B) 48 h after training in Experiment 2 (grey shading), during which mice underwent six weeks of diet manipulation; and C) four weeks after training in Experiment 3, during which mice underwent six total weeks of diet manipulation (training occurred after two weeks of diet manipulation). Experiment 1: 0.4% NaCl females Training Context, n=7; 0.4% NaCl females Neutral Context, n=9; 4.0% NaCl females Training Context, n=8; 4.0% NaCl females Neutral Context, n=9; 0.4% NaCl males Training Context, n=8; 0.4% NaCl males Neutral Context, n=9; 4.0% NaCl males Training Context, n=7; 4.0% NaCl males Neutral Context, n=8. Experiment 2: 0.4% NaCl females Training Context, n=9; 0.4% NaCl females Neutral Context, n=8; 4.0% NaCl females Training Context, n=8; 4.0% NaCl females Neutral Context, n=8; 0.4% NaCl males Training Context, n=9; 0.4% NaCl males Neutral Context, n=8; 4.0% NaCl males Training Context, n=8; 4.0% NaCl males Neutral Context, n=10. Experiment 3: 0.4% NaCl females Training Context, n=8; 0.4% NaCl females Neutral Context, n=9; 4.0% NaCl females Training Context, n=9; 4.0% NaCl females Neutral Context, n=8; 0.4% NaCl males Training Context, n=7; 0.4% NaCl males Neutral Context, n=8; 4.0% NaCl males Training Context, n=8; 4.0% NaCl males Neutral Context, n=8. Data are graphed as mean ± 95% confidence interval; pairwise comparisons were made using Bonferroni correction. Thick symbol borders on Training Context data indicate significant ($p<0.05$) differences between Training and Neutral Contexts for that specific diet/sex combination. *$p<0.05$, **$p<0.01$, ***$p<0.001$ indicate difference between 0.4% and 4.0% NaCl within Training Context and same

sex. ‡p<0.05, ‡‡p<0.01, ‡‡‡p<0.001 indicate difference between 0.4% and 4.0% NaCl within Neutral Context and same sex.
