# Supplementary material for: Generalization of contextual fear is sex-specifically affected by high salt intake
Source: PLoS One. 2023 Jul 13;18(7):e0286221. doi: 10.1371/journal.pone.0286221 (PMC10343085; doi:10.1371/journal.pone.0286221)
Supplement: S7 Fig — (PDF) [file pone.0286221.s040.pdf]

Supplemental Material for

Generalization of contextual fear is sex-specifically affected by high salt intake

Jasmin N. Beaver[1,2], Brady L. Weber[1,2], Matthew T. Ford[1], Anna E. Anello[1,2], Kaden M. Ruffin[1], Sarah K. Kassis[1,2], T. Lee Gilman[1,2,3]*

[1]Department of Psychological Sciences, Kent State University, Kent, Ohio, United States of America

[2]Brain Health Research Institute, Kent State University, Kent, Ohio, United States of America

[3]Healthy Communities Research Institute, Kent State University, Kent, Ohio, United States of America

*Corresponding Author

Email: lgilman1@kent.edu (TLG)

S7 Figure

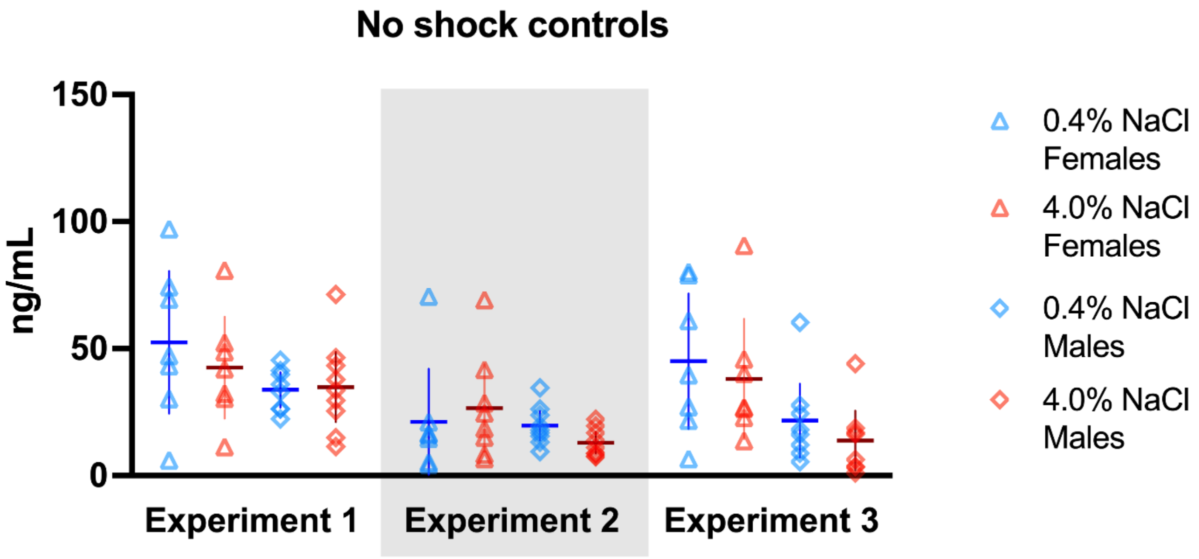

S7 Fig. Raw (pre-transformed) serum corticosterone levels of no shock control mice across Experiments.

Females represented by triangles, males by diamonds; 0.4% NaCl represented by blue symbols, 4.0% NaCl represented by red symbols. Experiment 1: 0.4% NaCl females, n=6; 4.0% NaCl females, n=7; 0.4% NaCl males, n=8; 4.0% NaCl males, n=9. Experiment 2 (grey shading): 0.4% NaCl females, n=7; 4.0% NaCl females, n=8; 0.4% NaCl males, n=9; 4.0% NaCl males, n=9. Experiment 3: 0.4% NaCl females, n=7; 4.0% NaCl females, n=7; 0.4% NaCl males, n=8; 4.0% NaCl males, n=8. Data are graphed as mean ± 95% confidence interval. These data were not statistically analyzed – log transformations were applied to normalize data distribution prior to analyzing statistically.
