# Supplementary material for: Generalization of contextual fear is sex-specifically affected by high salt intake
Source: PLoS One. 2023 Jul 13;18(7):e0286221. doi: 10.1371/journal.pone.0286221 (PMC10343085; doi:10.1371/journal.pone.0286221)
Supplement: S8 Fig — (PDF) [file pone.0286221.s041.pdf]

Supplemental Material for

Generalization of contextual fear is sex-specifically affected by high salt intake

Jasmin N. Beaver[1,2], Brady L. Weber[1,2], Matthew T. Ford[1], Anna E. Anello[1,2], Kaden M. Ruffin[1], Sarah K. Kassis[1,2], T. Lee Gilman[1,2,3*]

[1]Department of Psychological Sciences, Kent State University, Kent, Ohio, United States of America

[2]Brain Health Research Institute, Kent State University, Kent, Ohio, United States of America

[3]Healthy Communities Research Institute, Kent State University, Kent, Ohio, United States of America

*Corresponding Author

Email: lgilman1@kent.edu (TLG)

S8 Figure

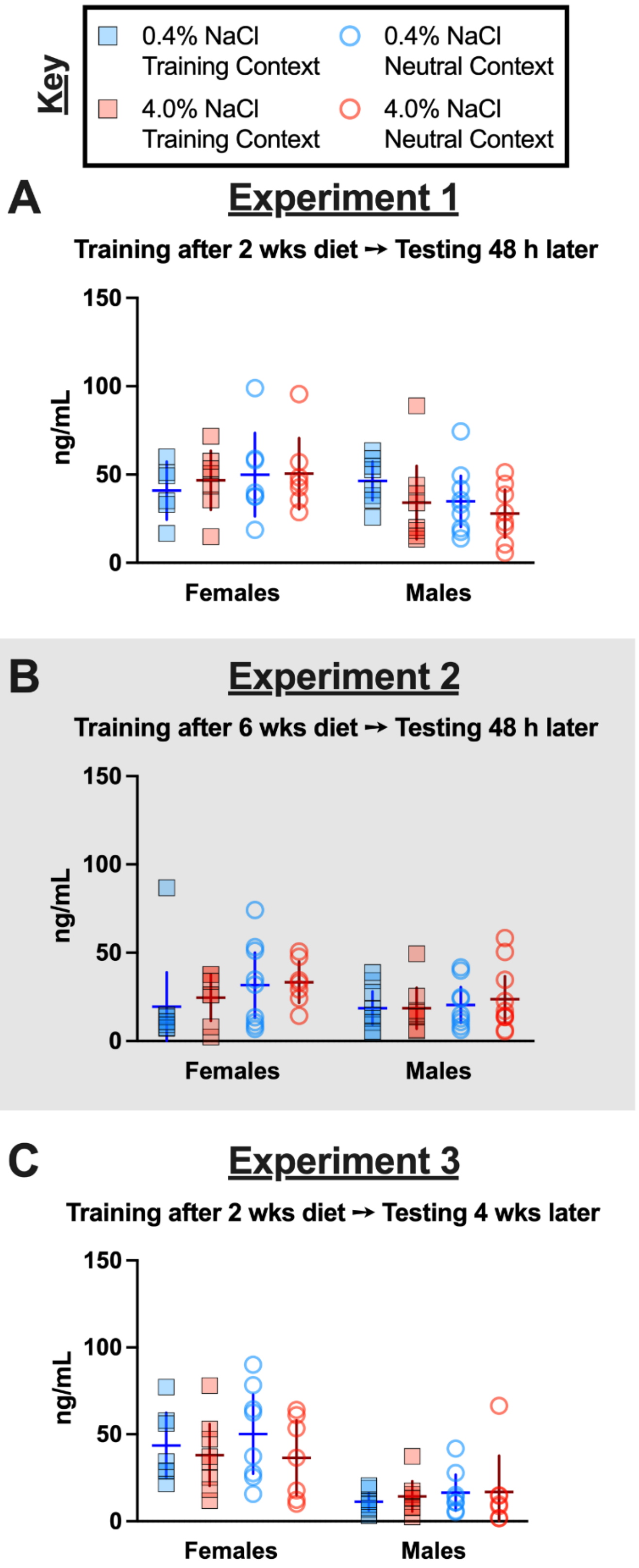
Key
0.4% NaCl Training Context
0.4% NaCl Neutral Context
4.0% NaCl Training Context
4.0% NaCl Neutral Context
A
Experiment 1
Training after 2 wks diet → Testing 48 h later
ng/mL
150
100
50
0
Females
Males
B
Experiment 2
Training after 6 wks diet → Testing 48 h later
ng/mL
150
100
50
0
Females
Males
C
Experiment 3
Training after 2 wks diet → Testing 4 wks later
ng/mL
150
100
50
0
Females
Males

S8 Fig. Raw (pre-transformed) serum corticosterone levels in fear conditioned mice across Experiments.

Mice assigned to 0.4% NaCl represented by blue symbols, mice assigned to 4.0% NaCl represented by red symbols; mice tested in Training Context represented by squares and solid lines, mice tested in Neutral Context represented by circles and dotted lines. Log-transformed serum corticosterone levels in context fear conditioned mice in A) Experiment 1, B) Experiment 2 (grey shading), and C) Experiment 3. Experiment 1: 0.4% NaCl females Training Context, n=6; 0.4% NaCl females Neutral Context, n=7; 4.0% NaCl females Training Context, n=7; 4.0% NaCl females Neutral Context, n=7; 0.4% NaCl males Training Context, n=8; 0.4% NaCl males Neutral Context, n=9; 4.0% NaCl males Training Context, n=8; 4.0% NaCl males Neutral Context, n=8. Experiment 2: 0.4% NaCl females Training Context, n=8; 0.4% NaCl females Neutral Context, n=9; 4.0% NaCl females Training Context, n=7; 4.0% NaCl females Neutral Context, n=7; 0.4% NaCl males Training Context, n=9; 0.4% NaCl males Neutral Context, n=9; 4.0% NaCl males Training Context, n=8; 4.0% NaCl males Neutral Context, n=10. Experiment 3: 0.4% NaCl females Training Context, n=7; 0.4% NaCl females Neutral Context, n=8; 4.0% NaCl females Training Context, n=8; 4.0% NaCl females Neutral Context, n=7; 0.4% NaCl males Training Context, n=8; 0.4% NaCl males Neutral Context, n=8; 4.0% NaCl males Training Context, n=8; 4.0% NaCl males Neutral Context, n=7. Data are graphed as mean ± 95% confidence interval. These data were not statistically analyzed – log transformations were applied to normalize data distribution prior to analyzing statistically.
