# Supplementary material for: Generalization of contextual fear is sex-specifically affected by high salt intake
Source: PLoS One. 2023 Jul 13;18(7):e0286221. doi: 10.1371/journal.pone.0286221 (PMC10343085; doi:10.1371/journal.pone.0286221)
Supplement: S12 Fig — (PDF) [file pone.0286221.s045.pdf]

**Supplemental Material for**

# Generalization of contextual fear is sex-specifically affected by high salt intake

Jasmin N. Beaver[1,2], Brady L. Weber[1,2], Matthew T. Ford[1], Anna E. Anello[1,2], Kaden M. Ruffin[1], Sarah K. Kassis[1,2], T. Lee Gilman[1,2,3*]

[1]Department of Psychological Sciences, Kent State University, Kent, Ohio, United States of America

[2]Brain Health Research Institute, Kent State University, Kent, Ohio, United States of America

[3]Healthy Communities Research Institute, Kent State University, Kent, Ohio, United States of America

*Corresponding Author

Email: lgilman1@kent.edu (TLG)

S12 Figure

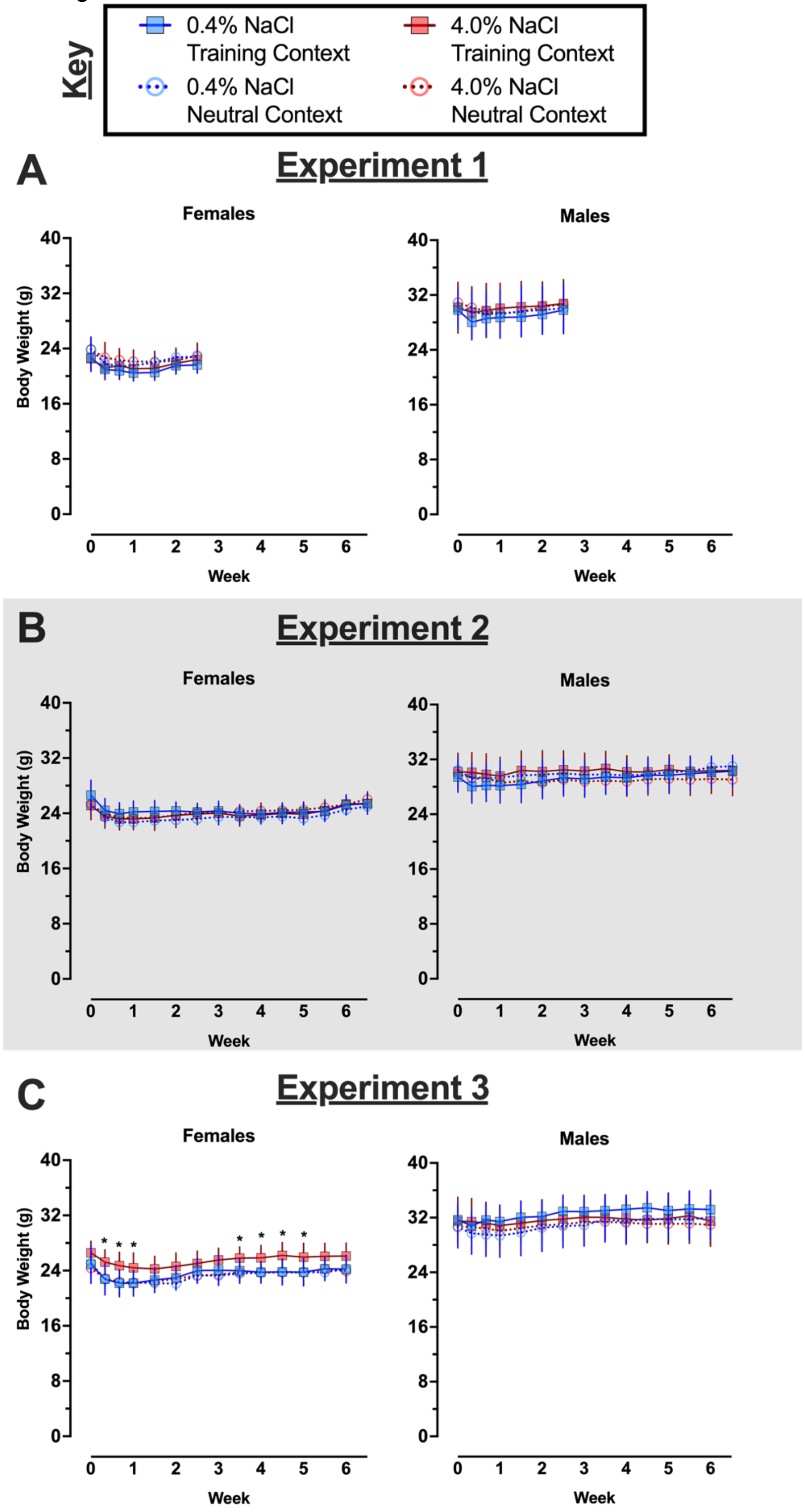
Key
0.4% NaCl Training Context
4.0% NaCl Training Context
0.4% NaCl Neutral Context
4.0% NaCl Neutral Context
A
Experiment 1
Females
Males
Body Weight (g)
Week
B
Experiment 2
Females
Males
Body Weight (g)
Week
C
Experiment 3
Females
Males
Body Weight (g)
Week

**S12 Fig. Body weight of context fear conditioned mice across Experiments.**

Mice assigned to 0.4% NaCl represented by blue symbols, mice assigned to 4.0% NaCl represented by red symbols; mice to be tested in Training Context represented by squares and solid lines, mice to be tested in Neutral Context represented by circles and dotted lines. Body weights were measured twice weekly of mice in A) Experiment 1, B) Experiment 2 (grey shading), and C) Experiment 3. Some data loss occurred on the very last weighing day for a subset of animals in C) Experiment 3, thus graphs and repeated measures statistical analyses for Experiment 3 body weights cease at week 6 to maximize inclusion of mice in repeated measures analyses. Experiment 1: 0.4% NaCl females Training Context, n=8; 0.4% NaCl females Neutral Context, n=9; 4.0% NaCl females Training Context, n=8; 4.0% NaCl females Neutral Context, n=9; 0.4% NaCl males Training Context, n=8; 0.4% NaCl males Neutral Context, n=9; 4.0% NaCl males Training Context, n=8; 4.0% NaCl males Neutral Context, n=8. Experiment 2: 0.4% NaCl females Training Context, n=9; 0.4% NaCl females Neutral Context, n=9; 4.0% NaCl females Training Context, n=8; 4.0% NaCl females Neutral Context, n=8; 0.4% NaCl males Training Context, n=9; 0.4% NaCl males Neutral Context, n=9; 4.0% NaCl males Training Context, n=8; 4.0% NaCl males Neutral Context, n=10. Experiment 3: 0.4% NaCl females Training Context, n=8; 0.4% NaCl females Neutral Context, n=9; 4.0% NaCl females Training Context, n=9; 4.0% NaCl females Neutral Context, n=8; 0.4% NaCl males Training Context, n=8; 0.4% NaCl males Neutral Context, n=8; 4.0% NaCl males Training Context, n=8; 4.0% NaCl males Neutral Context, n=8. Data are graphed as mean ± 95% confidence interval. *$p<0.05$ indicates difference between mice within the same sex consuming 0.4% NaCl versus 4.0% NaCl and tested in Training Context.
