# Supplementary material for: Generalization of contextual fear is sex-specifically affected by high salt intake
Source: PLoS One. 2023 Jul 13;18(7):e0286221. doi: 10.1371/journal.pone.0286221 (PMC10343085; doi:10.1371/journal.pone.0286221)
Supplement: S13 Fig — (PDF) [file pone.0286221.s046.pdf]

Supplemental Material for

Generalization of contextual fear is sex-specifically affected by high salt intake

Jasmin N. Beaver[1,2], Brady L. Weber[1,2], Matthew T. Ford[1], Anna E. Anello[1,2], Kaden M. Ruffin[1], Sarah K. Kassis[1,2], T. Lee Gilman[1,2,3]*

[1]Department of Psychological Sciences, Kent State University, Kent, Ohio, United States of America

[2]Brain Health Research Institute, Kent State University, Kent, Ohio, United States of America

[3]Healthy Communities Research Institute, Kent State University, Kent, Ohio, United States of America

*Corresponding Author

Email: lgilman1@kent.edu (TLG)

S13 Figure

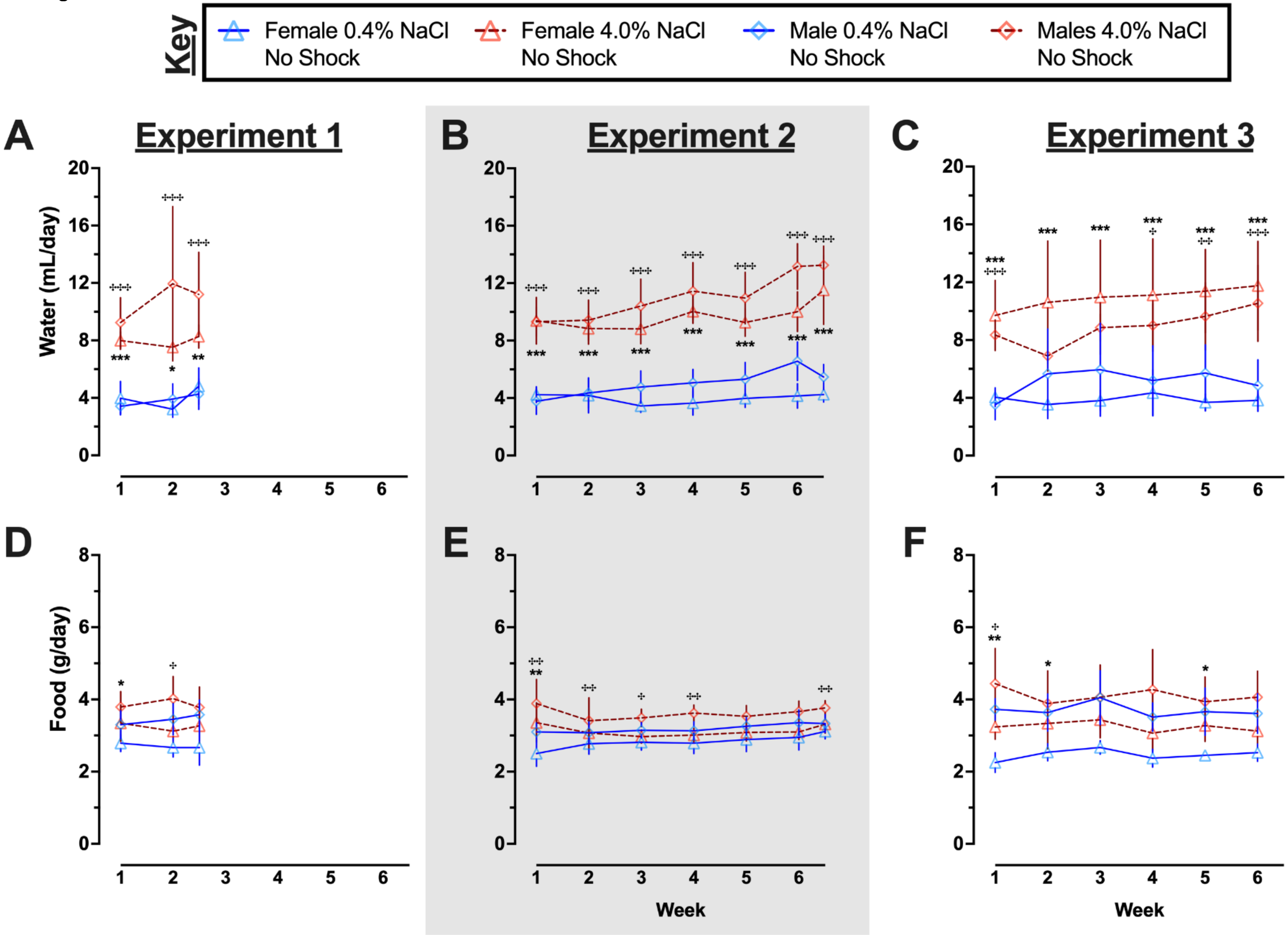

Key
Female 0.4% NaCl No Shock
Female 4.0% NaCl No Shock
Male 0.4% NaCl No Shock
Males 4.0% NaCl No Shock
A
Experiment 1
B
Experiment 2
C
Experiment 3
Water (mL/day)
D
E
F
Food (g/day)
Week
Week

S13 Fig. Average water and food consumed per day by control no shock mice across Experiments.

Females represented by triangles, males by diamonds; 0.4% NaCl represented by blue symbols and solid lines, 4.0% NaCl represented by red symbols and dashed lines. A, B, C) Water and D, E, F) food consumption was measured twice weekly, and full weeks were averaged; partial weeks at the conclusion of A, D) Experiment 1 and B, E) Experiment 2 (grey shading) are included in the graphs. Some data loss occurred on the very last weighing day for a subset of animals in C, F) Experiment 3, thus graphs and repeated measures statistical analyses for Experiment 3 consumption cease at week 6 to maximize inclusion of mice in repeated measures analyses. Experiment 1: 0.4% NaCl females, n=9; 4.0% NaCl females, n=9; 0.4% NaCl males, n=8; 4.0% NaCl males, n=9. Experiment 2: 0.4% NaCl females, n=8; 4.0% NaCl females, n=7; 0.4% NaCl males, n=9; 4.0% NaCl males, n=9. Experiment 3: 0.4% NaCl females, n=8; 4.0% NaCl females, n=8; 0.4% NaCl males, n=8; 4.0% NaCl males, n=8. Data are graphed as mean ± 95% confidence interval. *p<0.05, **p<0.01, ***p<0.001 indicate difference between females consuming 0.4% NaCl versus 4.0% NaCl. ✢p<0.05, ✢✢p<0.01, ✢✢✢p<0.001 indicate difference between males consuming 0.4% NaCl versus 4.0% NaCl.
