# Supplementary material for: Generalization of contextual fear is sex-specifically affected by high salt intake
Source: PLoS One. 2023 Jul 13;18(7):e0286221. doi: 10.1371/journal.pone.0286221 (PMC10343085; doi:10.1371/journal.pone.0286221)
Supplement: S14 Fig — (PDF) [file pone.0286221.s047.pdf]

Supplemental Material for

Generalization of contextual fear is sex-specifically affected by high salt intake

Jasmin N. Beaver[1,2], Brady L. Weber[1,2], Matthew T. Ford[1], Anna E. Anello[1,2], Kaden M. Ruffin[1], Sarah K. Kassis[1,2], T. Lee Gilman[1,2,3]*

[1]Department of Psychological Sciences, Kent State University, Kent, Ohio, United States of America

[2]Brain Health Research Institute, Kent State University, Kent, Ohio, United States of America

[3]Healthy Communities Research Institute, Kent State University, Kent, Ohio, United States of America

*Corresponding Author

Email: lgilman1@kent.edu (TLG)

S14 Figure

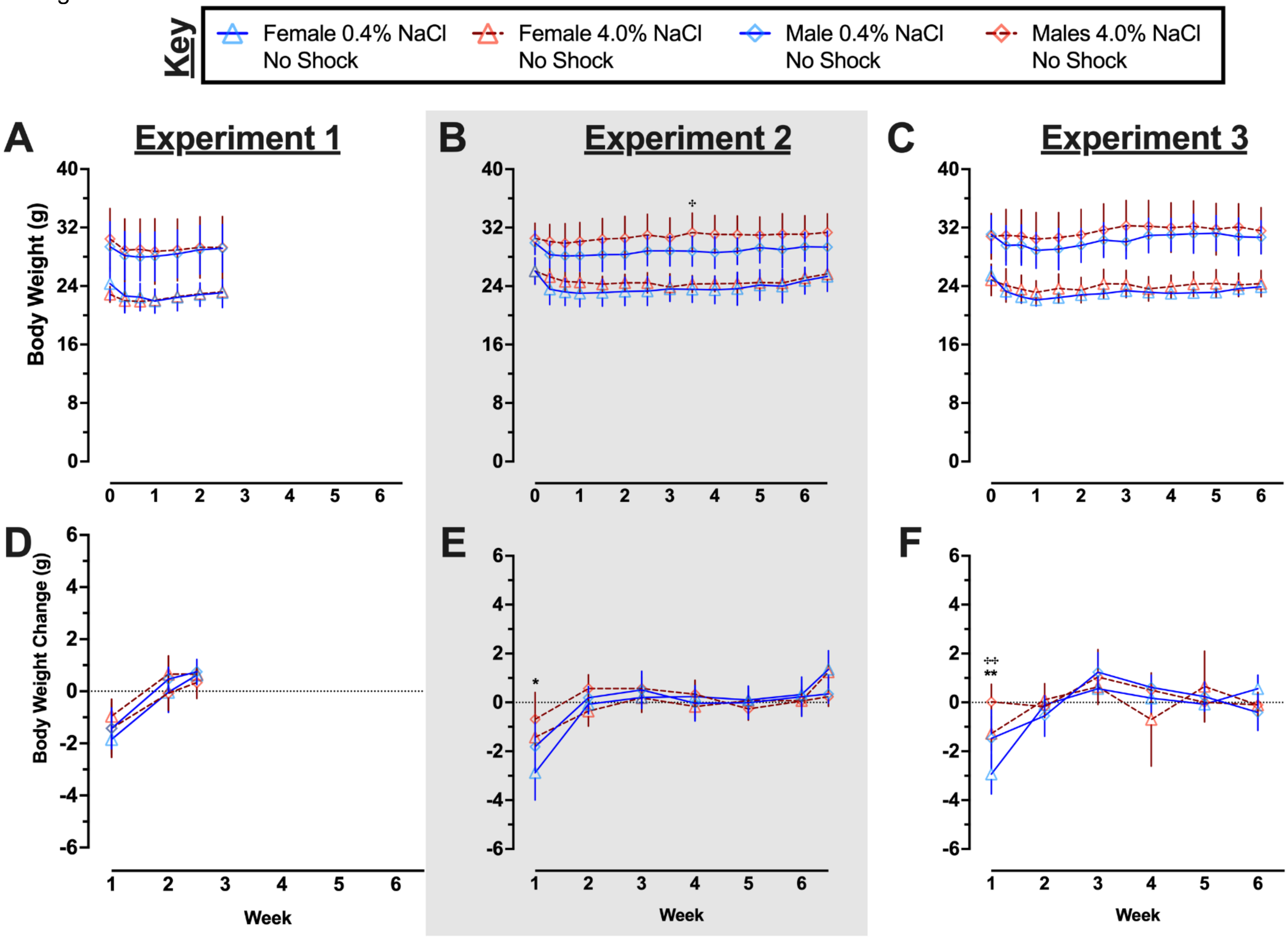
Key
Female 0.4% NaCl No Shock
Female 4.0% NaCl No Shock
Male 0.4% NaCl No Shock
Males 4.0% NaCl No Shock
A
Experiment 1
B
Experiment 2
C
Experiment 3
Body Weight (g)
D
E
F
Body Weight Change (g)
Week

S14 Fig. Body weights and body weight changes in control no shock mice across Experiments.

Females represented by triangles, males by diamonds; 0.4% NaCl represented by blue symbols and solid lines, 4.0% NaCl represented by red symbols and dashed lines. A, B, C) Body weights were measured twice weekly, and D, E, F) body weight changes were calculated across full weeks. Partial weeks at the conclusion of A, D) Experiment 1 and B, E) Experiment 2 (grey shading) are included in the graphs. Some data loss occurred on the very last weighing day for a subset of animals in C, F) Experiment 3, thus graphs and repeated measures statistical analyses for Experiment 3 consumption cease at week 6 to maximize inclusion of mice in repeated measures analyses. Experiment 1: 0.4% NaCl females, n=9; 4.0% NaCl females, n=9; 0.4% NaCl males, n=8; 4.0% NaCl males, n=9. Experiment 2: 0.4% NaCl females, n=8; 4.0% NaCl females, n=7; 0.4% NaCl males, n=9; 4.0% NaCl males, n=9. Experiment 3: 0.4% NaCl females, n=8; 4.0% NaCl females, n=8; 0.4% NaCl males, n=8; 4.0% NaCl males, n=8. Data are graphed as mean ± 95% confidence interval. *$p<0.05$, **$p<0.01$, indicate difference between females consuming 0.4% NaCl versus 4.0% NaCl. $^{+}p<0.05$, $^{++}p<0.01$, indicate difference between males consuming 0.4% NaCl versus 4.0% NaCl.
