# Supplementary material for: Generalization of contextual fear is sex-specifically affected by high salt intake
Source: PLoS One. 2023 Jul 13;18(7):e0286221. doi: 10.1371/journal.pone.0286221 (PMC10343085; doi:10.1371/journal.pone.0286221)
Supplement: S18 Fig — (PDF) [file pone.0286221.s051.pdf]

Supplemental Material for

Generalization of contextual fear is sex-specifically affected by high salt intake

Jasmin N. Beaver[1,2], Brady L. Weber[1,2], Matthew T. Ford[1], Anna E. Anello[1,2], Kaden M. Ruffin[1], Sarah K. Kassis[1,2], T. Lee Gilman[1,2,3]*

[1]Department of Psychological Sciences, Kent State University, Kent, Ohio, United States of America

[2]Brain Health Research Institute, Kent State University, Kent, Ohio, United States of America

[3]Healthy Communities Research Institute, Kent State University, Kent, Ohio, United States of America

*Corresponding Author

Email: lgilman1@kent.edu (TLG)

S18 Figure

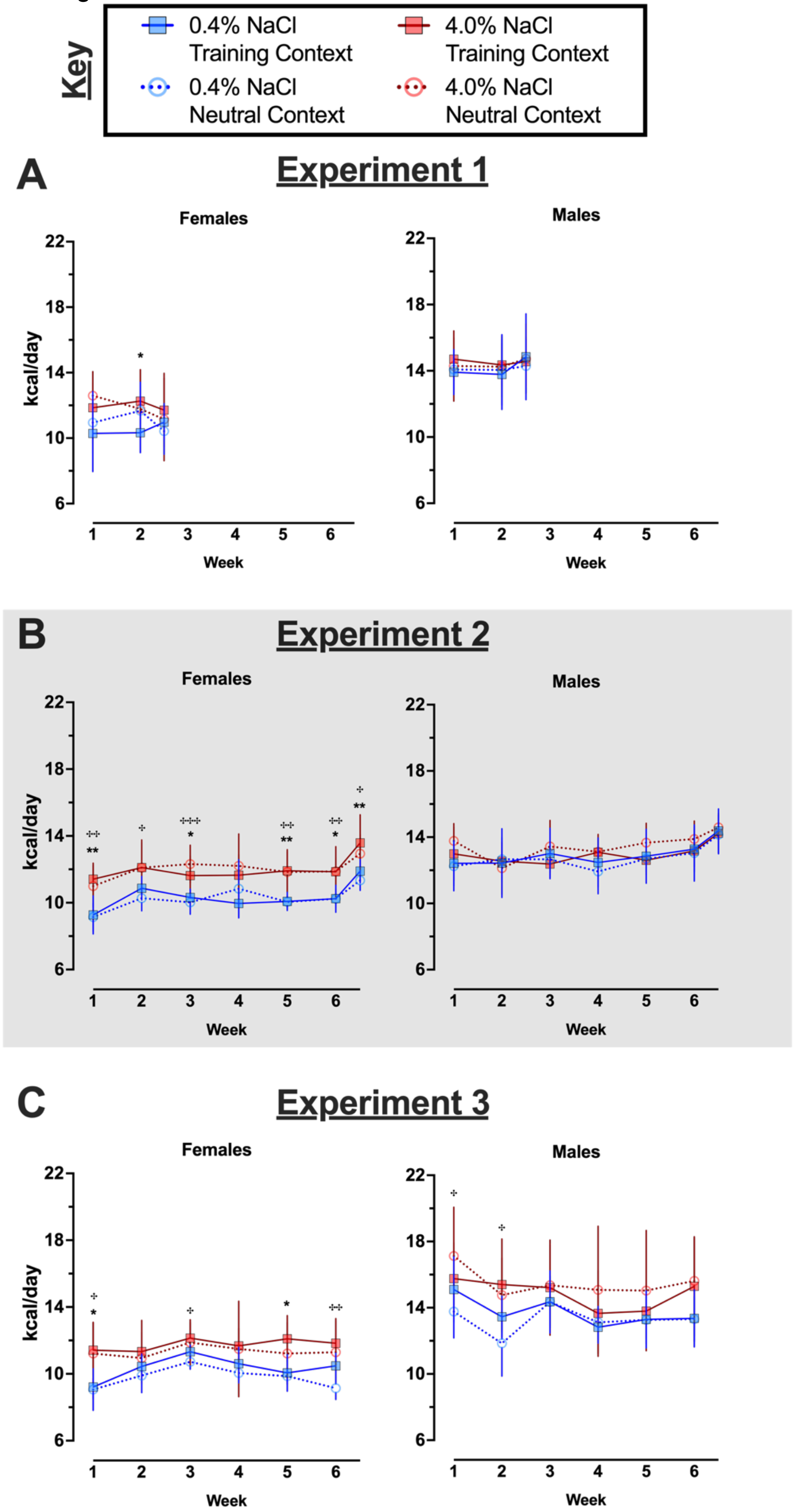
Key
0.4% NaCl Training Context
4.0% NaCl Training Context
0.4% NaCl Neutral Context
4.0% NaCl Neutral Context
A
Experiment 1
Females
Males
kcal/day
Week
B
Experiment 2
Females
Males
kcal/day
Week
C
Experiment 3
Females
Males
kcal/day
Week

S18 Fig. Average kcal consumed per day by context fear conditioned mice across Experiments.

Mice assigned to 0.4% NaCl represented by blue symbols, mice assigned to 4.0% NaCl represented by red symbols; mice to be tested in Training Context represented by squares and solid lines, mice to be tested in Neutral Context represented by circles and dotted lines. Consumption of kcal was calculated based upon the amount of each diet consumed per day and the diet’s respective kcal (3.89 kcal/g for 0.4% NaCl diet; 3.75 kcal/g for 4.0% NaCl diet). These calculations were made for each full week, plus the partial weeks at the conclusion of A) Experiment 1 and B) Experiment 2 (grey shading). Some data loss occurred on the very last weighing day for a subset of animals in C) Experiment 3, thus graphs and repeated measures statistical analyses for Experiment 3 calculations cease at week 6 to maximize inclusion of mice in repeated measures analyses. Experiment 1: 0.4% NaCl females Training Context, n=8; 0.4% NaCl females Neutral Context, n=9; 4.0% NaCl females Training Context, n=8; 4.0% NaCl females Neutral Context, n=9; 0.4% NaCl males Training Context, n=8; 0.4% NaCl males Neutral Context, n=9; 4.0% NaCl males Training Context, n=8; 4.0% NaCl males Neutral Context, n=8. Experiment 2: 0.4% NaCl females Training Context, n=9; 0.4% NaCl females Neutral Context, n=9; 4.0% NaCl females Training Context, n=8; 4.0% NaCl females Neutral Context, n=8; 0.4% NaCl males Training Context, n=9; 0.4% NaCl males Neutral Context, n=9; 4.0% NaCl males Training Context, n=8; 4.0% NaCl males Neutral Context, n=10. Experiment 3: 0.4% NaCl females Training Context, n=8; 0.4% NaCl females Neutral Context, n=9; 4.0% NaCl females Training Context, n=9; 4.0% NaCl females Neutral Context, n=8; 0.4% NaCl males Training Context, n=8; 0.4% NaCl males Neutral Context, n=8; 4.0% NaCl males Training Context, n=8; 4.0% NaCl males Neutral Context, n=8. Data are graphed as mean ± 95% confidence interval. *$p<0.05$, **$p<0.01$, ***$p<0.001$ indicate difference between mice within the same sex consuming 0.4% NaCl versus 4.0% NaCl and tested in Training Context. ‡$p<0.05$,

$^{✢✢}p<0.01$, $^{✢✢✢}p<0.001$ indicate difference between mice within the same sex consuming 0.4% NaCl versus 4.0% NaCl and tested in Neutral Context.
