# Supplementary material for: Generalization of contextual fear is sex-specifically affected by high salt intake
Source: PLoS One. 2023 Jul 13;18(7):e0286221. doi: 10.1371/journal.pone.0286221 (PMC10343085; doi:10.1371/journal.pone.0286221)
Supplement: S20 Fig — (PDF) [file pone.0286221.s053.pdf]

**Supplemental Material for**

# Generalization of contextual fear is sex-specifically affected by high salt intake

Jasmin N. Beaver[1,2], Brady L. Weber[1,2], Matthew T. Ford[1], Anna E. Anello[1,2], Kaden M. Ruffin[1], Sarah K. Kassis[1,2], T. Lee Gilman[1,2,3*]

[1]Department of Psychological Sciences, Kent State University, Kent, Ohio, United States of America

[2]Brain Health Research Institute, Kent State University, Kent, Ohio, United States of America

[3]Healthy Communities Research Institute, Kent State University, Kent, Ohio, United States of America

*Corresponding Author

Email: lgilman1@kent.edu (TLG)

S20 Figure

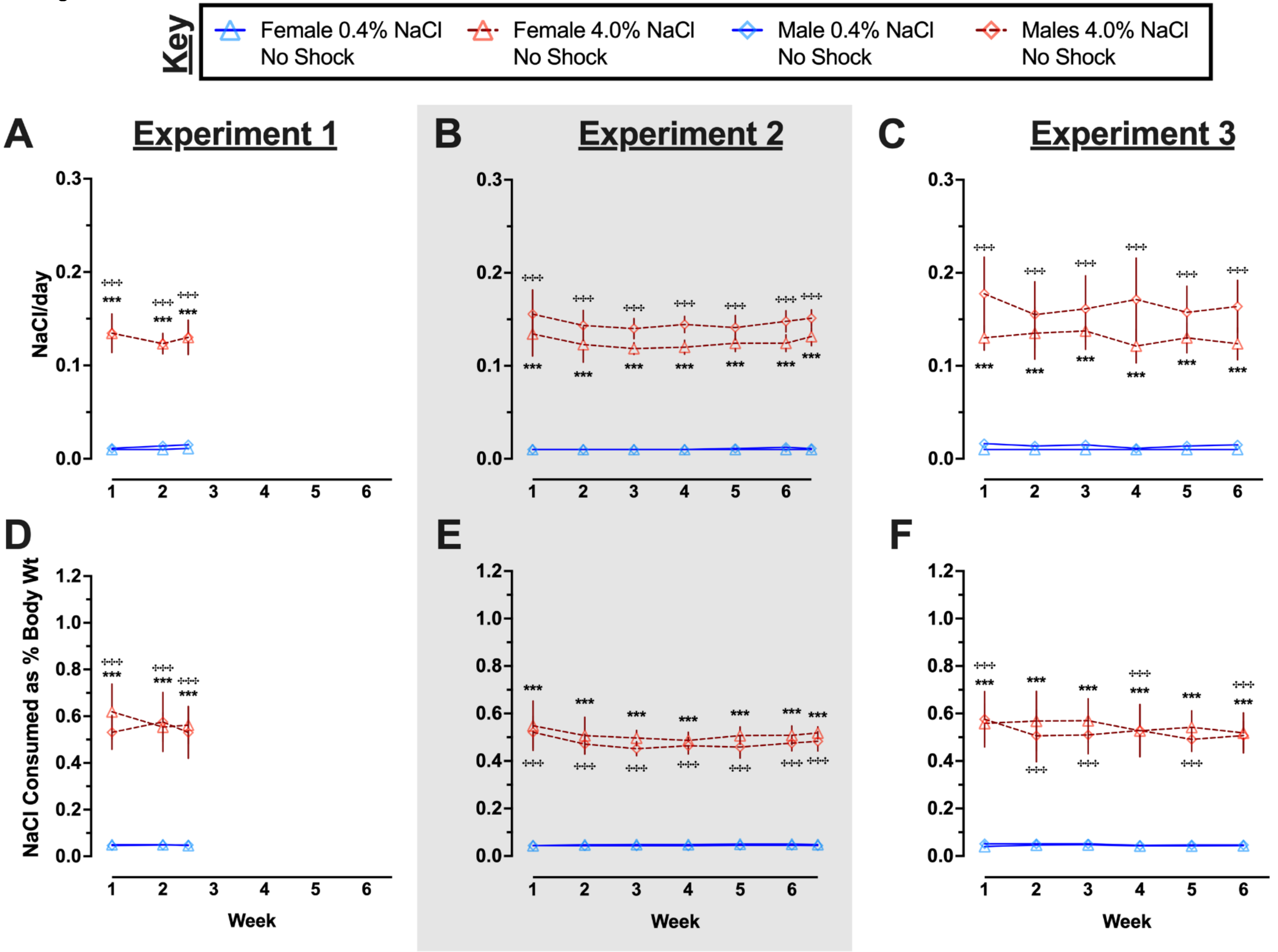

Key
Female 0.4% NaCl No Shock
Female 4.0% NaCl No Shock
Male 0.4% NaCl No Shock
Males 4.0% NaCl No Shock
A
Experiment 1
B
Experiment 2
C
Experiment 3
NaCl/day
0.3
0.2
0.1
0.0
D
E
F
NaCl Consumed as % Body Wt
1.2
1.0
0.8
0.6
0.4
0.2
0.0
1 2 3 4 5 6
Week

**S20 Fig. Average NaCl consumed per day and NaCl consumed as a percentage of body weight by control no shock mice across Experiments.**

Females represented by triangles, males by diamonds; 0.4% NaCl represented by blue symbols and solid lines, 4.0% NaCl represented by red symbols and dashed lines. A, B, C) NaCl consumed per day and D, E, F) NaCl consumption as a percentage of body weight were calculated for each full week, and for the partial week at the conclusion of A, D) Experiment 1 and B, E) Experiment 2 (grey shading). Some data loss occurred on the very last weighing day for a subset of animals in C, F) Experiment 3, thus graphs and repeated measures statistical analyses for Experiment 3 calculations cease at week 6 to maximize inclusion of mice in repeated measures analyses. Experiment 1: 0.4% NaCl females, n=9; 4.0% NaCl females, n=9; 0.4% NaCl males, n=8; 4.0% NaCl males, n=9. Experiment 2: 0.4% NaCl females, n=8; 4.0% NaCl females, n=7; 0.4% NaCl males, n=9; 4.0% NaCl males, n=9. Experiment 3: 0.4% NaCl females, n=8; 4.0% NaCl females, n=8; 0.4% NaCl males, n=8; 4.0% NaCl males, n=8. Data are graphed as mean ± 95% confidence interval. *$p<0.05$, **$p<0.01$, ***$p<0.001$ indicate difference between females consuming 0.4% NaCl versus 4.0% NaCl. $^{\ddagger}p<0.05$, $^{\ddagger\ddagger}p<0.01$, $^{\ddagger\ddagger\ddagger}p<0.001$ indicate difference between males consuming 0.4% NaCl versus 4.0% NaCl.
