# Supplementary material for: Generalization of contextual fear is sex-specifically affected by high salt intake
Source: PLoS One. 2023 Jul 13;18(7):e0286221. doi: 10.1371/journal.pone.0286221 (PMC10343085; doi:10.1371/journal.pone.0286221)
Supplement: S22 Fig — (PDF) [file pone.0286221.s055.pdf]

Supplemental Material for

Generalization of contextual fear is sex-specifically affected by high salt intake

Jasmin N. Beaver[1,2], Brady L. Weber[1,2], Matthew T. Ford[1], Anna E. Anello[1,2], Kaden M. Ruffin[1], Sarah K. Kassis[1,2], T. Lee Gilman[1,2,3]*

[1]Department of Psychological Sciences, Kent State University, Kent, Ohio, United States of America

[2]Brain Health Research Institute, Kent State University, Kent, Ohio, United States of America

[3]Healthy Communities Research Institute, Kent State University, Kent, Ohio, United States of America

*Corresponding Author

Email: lgilman1@kent.edu (TLG)

S22 Figure

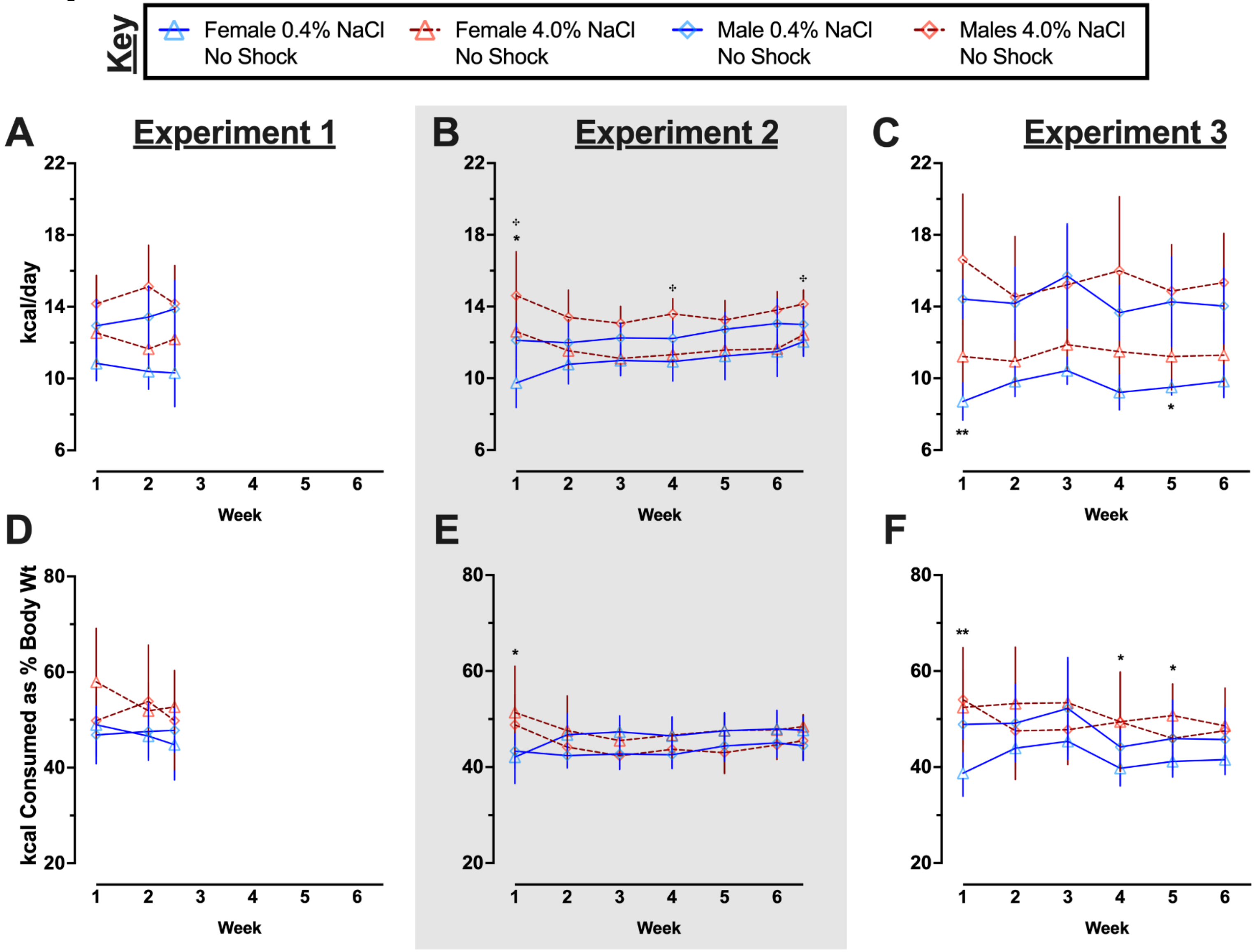

Key
Female 0.4% NaCl No Shock
Female 4.0% NaCl No Shock
Male 0.4% NaCl No Shock
Males 4.0% NaCl No Shock
A
Experiment 1
B
Experiment 2
C
Experiment 3
kcal/day
22
18
14
10
6
1
2
3
4
5
6
Week
D
E
F
kcal Consumed as % Body Wt
80
60
40
20

S22 Fig. Average kcal consumed per day and kcal consumed as a percentage of body weight by control no shock mice across Experiments.

Females represented by triangles, males by diamonds; 0.4% NaCl represented by blue symbols and solid lines, 4.0% NaCl represented by red symbols and dashed lines. A, B, C) Consumption of kcal per day and D, E, F) kcal consumption as a percentage of body weight were calculated for each full week, and for the partial week at the conclusion of A, D) Experiment 1 and B, E) Experiment 2 (grey shading). Some data loss occurred on the very last weighing day for a subset of animals in C, F) Experiment 3, thus graphs and repeated measures statistical analyses for Experiment 3 calculations cease at week 6 to maximize inclusion of mice in repeated measures analyses. Experiment 1: 0.4% NaCl females, n=9; 4.0% NaCl females, n=9; 0.4% NaCl males, n=8; 4.0% NaCl males, n=9. Experiment 2: 0.4% NaCl females, n=8; 4.0% NaCl females, n=7; 0.4% NaCl males, n=9; 4.0% NaCl males, n=9. Experiment 3: 0.4% NaCl females, n=8; 4.0% NaCl females, n=8; 0.4% NaCl males, n=8; 4.0% NaCl males, n=8. Data are graphed as mean ± 95% confidence interval. *p<0.05, **p<0.01, ***p<0.001 indicate difference between females consuming 0.4% NaCl versus 4.0% NaCl. ‡p<0.05, ‡‡p<0.01, ‡‡‡p<0.001 indicate difference between males consuming 0.4% NaCl versus 4.0% NaCl.
