# Supplementary material for: Generalization of contextual fear is sex-specifically affected by high salt intake
Source: PLoS One. 2023 Jul 13;18(7):e0286221. doi: 10.1371/journal.pone.0286221 (PMC10343085; doi:10.1371/journal.pone.0286221)
Supplement: S23 Fig — (PDF) [file pone.0286221.s056.pdf]

Supplemental Material for

Generalization of contextual fear is sex-specifically affected by high salt intake

Jasmin N. Beaver[1,2], Brady L. Weber[1,2], Matthew T. Ford[1], Anna E. Anello[1,2], Kaden M. Ruffin[1], Sarah K. Kassis[1,2], T. Lee Gilman[1,2,3]*

[1]Department of Psychological Sciences, Kent State University, Kent, Ohio, United States of America

[2]Brain Health Research Institute, Kent State University, Kent, Ohio, United States of America

[3]Healthy Communities Research Institute, Kent State University, Kent, Ohio, United States of America

*Corresponding Author

Email: lgilman1@kent.edu (TLG)

S23 Figure

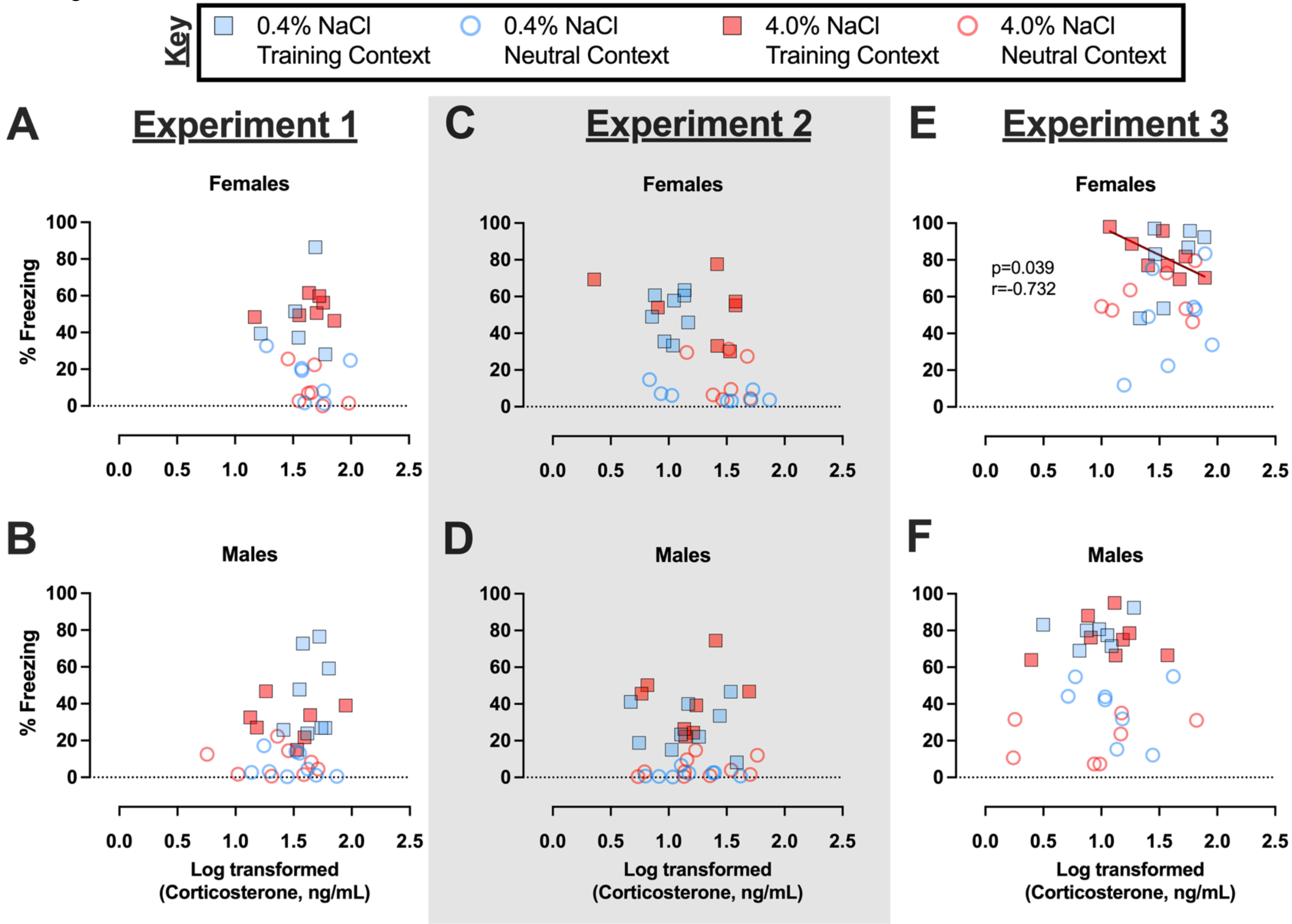

Key
0.4% NaCl Training Context
0.4% NaCl Neutral Context
4.0% NaCl Training Context
4.0% NaCl Neutral Context
A
Experiment 1
Females
% Freezing
B
Males
Log transformed (Corticosterone, ng/mL)
C
Experiment 2
D
E
Experiment 3
p=0.039
r=-0.732
F
0.0 0.5 1.0 1.5 2.0 2.5
0 20 40 60 80 100

S23 Fig. Correlations between context fear expression and log-transformed serum corticosterone levels across contexts and Experiments.

Mice assigned to 0.4% NaCl represented by blue symbols, mice assigned to 4.0% NaCl represented by red symbols; mice tested in Training Context represented by squares, mice tested in Neutral Context represented by circles. Individual log-transformed serum corticosterone levels were plotted on the x-axis against the same individual's contextual fear expression during minutes two through six of the 10 min testing session plotted on the y-axis. Data for each sex were graphed for A, B) Experiment 1, C, D) Experiment 2 (grey shading), and E, F) Experiment 3. Significant correlation indicated with solid line (female). Experiment 1: 0.4% NaCl females Training Context, n=5; 0.4% NaCl females Neutral Context, n=7; 4.0% NaCl females Training Context, n=7; 4.0% NaCl females Neutral Context, n=7; 0.4% NaCl males Training Context, n=8; 0.4% NaCl males Neutral Context, n=9; 4.0% NaCl males Training Context, n=7; 4.0% NaCl males Neutral Context, n=8. Experiment 2: 0.4% NaCl females Training Context, n=8; 0.4% NaCl females Neutral Context, n=8; 4.0% NaCl females Training Context, n=7; 4.0% NaCl females Neutral Context, n=7; 0.4% NaCl males Training Context, n=9; 0.4% NaCl males Neutral Context, n=8; 4.0% NaCl males Training Context, n=8; 4.0% NaCl males Neutral Context, n=10. Experiment 3: 0.4% NaCl females Training Context, n=7; 0.4% NaCl females Neutral Context, n=8; 4.0% NaCl females Training Context, n=8; 4.0% NaCl females Neutral Context, n=7; 0.4% NaCl males Training Context, n=7; 0.4% NaCl males Neutral Context, n=8; 4.0% NaCl males Training Context, n=8; 4.0% NaCl males Neutral Context, n=7.
