# Supplementary material for: Generalization of contextual fear is sex-specifically affected by high salt intake
Source: PLoS One. 2023 Jul 13;18(7):e0286221. doi: 10.1371/journal.pone.0286221 (PMC10343085; doi:10.1371/journal.pone.0286221)
Supplement: S24 Fig — (PDF) [file pone.0286221.s057.pdf]

Supplemental Material for

Generalization of contextual fear is sex-specifically affected by high salt intake

Jasmin N. Beaver[1,2], Brady L. Weber[1,2], Matthew T. Ford[1], Anna E. Anello[1,2], Kaden M. Ruffin[1], Sarah K. Kassis[1,2], T. Lee Gilman[1,2,3*]

[1]Department of Psychological Sciences, Kent State University, Kent, Ohio, United States of America

[2]Brain Health Research Institute, Kent State University, Kent, Ohio, United States of America

[3]Healthy Communities Research Institute, Kent State University, Kent, Ohio, United States of America

*Corresponding Author

Email: lgilman1@kent.edu (TLG)

S24 Figure

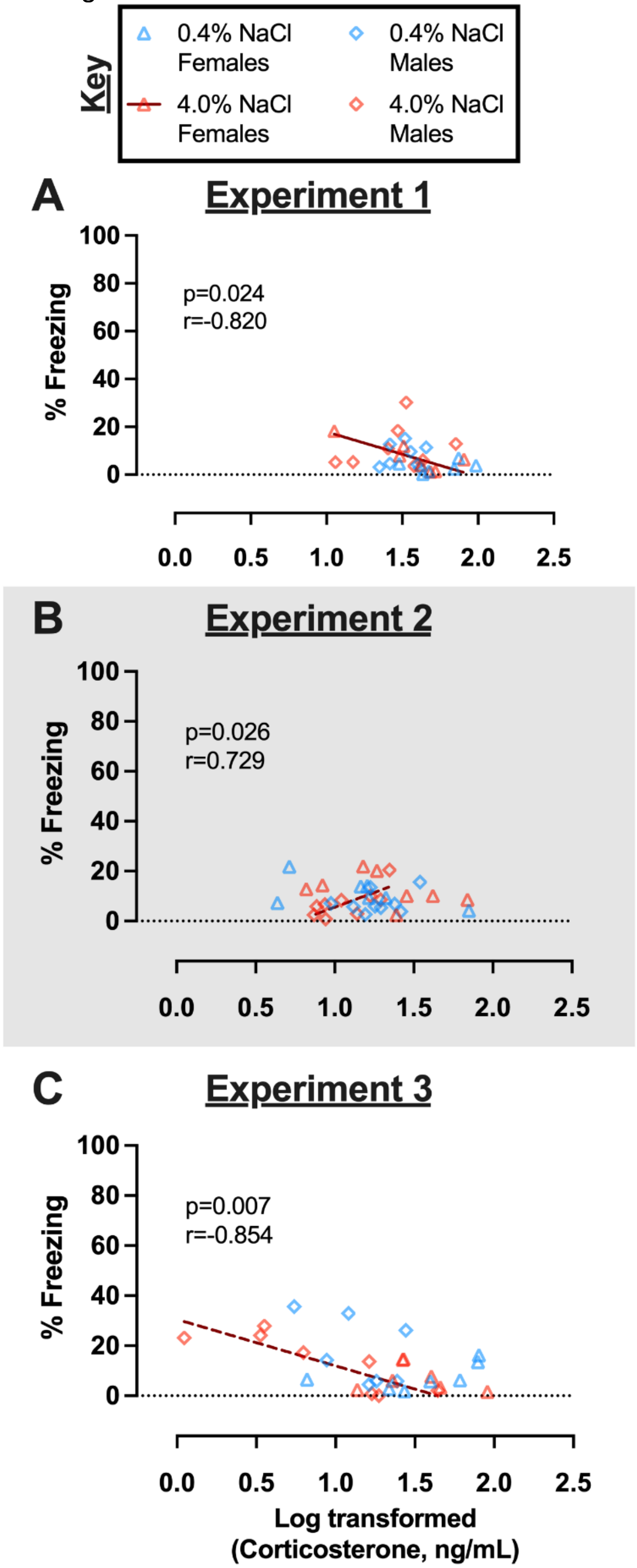

Key
0.4% NaCl Females
0.4% NaCl Males
4.0% NaCl Females
4.0% NaCl Males
A
Experiment 1
% Freezing
p=0.024
r=-0.820
100
80
60
40
20
0
0.0
0.5
1.0
1.5
2.0
2.5
B
Experiment 2
% Freezing
p=0.026
r=0.729
100
80
60
40
20
0
0.0
0.5
1.0
1.5
2.0
2.5
C
Experiment 3
% Freezing
p=0.007
r=-0.854
100
80
60
40
20
0
0.0
0.5
1.0
1.5
2.0
2.5
Log transformed
(Corticosterone, ng/mL)

S24 Figure. Correlations between context fear expression and log-transformed serum corticosterone levels in control no shock mice across Experiments.

Females represented by triangles, males by diamonds; 0.4% NaCl represented by blue symbols, 4.0% NaCl represented by red symbols. Individual log-transformed serum corticosterone levels were plotted on the x-axis against the same individual's contextual fear expression during minutes two through six of the 10 min testing session plotted on the y-axis. Data for each sex of control no shock mice were graphed for A) Experiment 1, B) Experiment 2 (grey shading), and C) Experiment 3. Significant correlations indicated with solid (female) or dashed (male) lines. Experiment 1: 0.4% NaCl females, n=6; 4.0% NaCl females, n=7; 0.4% NaCl males, n=8; 4.0% NaCl males, n=8. Experiment 2: 0.4% NaCl females, n=7; 4.0% NaCl females, n=8; 0.4% NaCl males, n=9; 4.0% NaCl males, n=9. Experiment 3: 0.4% NaCl females, n=7; 4.0% NaCl females, n=7; 0.4% NaCl males, n=7; 4.0% NaCl males, n=8.
