# Supplementary material for: Generalization of contextual fear is sex-specifically affected by high salt intake
Source: PLoS One. 2023 Jul 13;18(7):e0286221. doi: 10.1371/journal.pone.0286221 (PMC10343085; doi:10.1371/journal.pone.0286221)
Supplement: Attachment — Submitted filename: Response to Reviewers.pdf [file pone.0286221.s058.pdf]

Response to Reviewers - PONE-D-22-32310

Journal Requirements

When submitting your revision, we need you to address these additional requirements.

1. Please ensure that your manuscript meets PLOS ONE's style requirements, including those for file naming. The PLOS ONE style templates can be found at https://journals.plos.org/plosone/s/file?id=wjVg/PLOSOne_formatting_sample_main_body.pdf and https://journals.plos.org/plosone/s/file?id=ba62/PLOSOne_formatting_sample_title_authors_affiliations.pdf

Author response: We have now updated our title page, naming of Figure files, and the headings of our manuscript according to these requirements.

2. Thank you for stating the following financial disclosure:

Funding for this work was provided by Kent State University, and the Applied Psychology Center in the Department of Psychological Sciences at Kent State University.

Please state what role the funders took in the study. If the funders had no role, please state: "The funders had no role in study design, data collection and analysis, decision to publish, or preparation of the manuscript."
If this statement is not correct you must amend it as needed.
Please include this amended Role of Funder statement in your cover letter; we will change the online submission form on your behalf.

Author response: We have removed funding information from the manuscript file, and have added the Role of Funder in our cover letter. This states, as instructed above, "The funders had no role in study design, data collection and analysis, decision to publish, or preparation of the manuscript."

3. Thank you for stating the following in the Acknowledgments Section of your manuscript:

Funding for this work was provided by Kent State University, and the Applied Psychology Center in the Department of Psychological Sciences at Kent State University.

However, funding information should not appear in the Acknowledgments section or other areas of your manuscript. We will only publish funding information present in the Funding Statement section of the online submission form.
Please remove any funding-related text from the manuscript and let us know how you would like to update your Funding Statement. Currently, your Funding Statement reads as follows:

Funding for this work was provided by Kent State University, and the Applied Psychology Center in the Department of Psychological Sciences at Kent State University.

Please include your amended statements within your cover letter; we will change the online submission form on your behalf.

Author response: We have removed funding information from the manuscript file, and have added the Role of Funder in our cover letter. The full Funding Statement should read as follows:

> "*Funding for this work was provided by Kent State University, and the Applied Psychology Center in the Department of Psychological Sciences at Kent State University. The funders had no role in study design, data collection and analysis, decision to publish, or preparation of the manuscript.*"

4. Please amend your list of authors on the manuscript to ensure that each author is linked to an affiliation. Authors' affiliations should reflect the institution where the work was done (if authors moved subsequently, you can also list the new affiliation stating "current affiliation:…." as necessary).

Author response: We have revised our title page, linking each author to at least one individual affiliation at the time the work was done.

5. Please include captions for your Supporting Information files at the end of your manuscript, and update any in-text citations to match accordingly. Please see our Supporting Information guidelines for more information: http://journals.plos.org/plosone/s/supporting-information.

Author response: We have added in captions for Tables and Figures included in the Supporting Information file at the end of our manuscript. We've ensured that our in-text citations of same are still accurate.

Additional Editor Comments:

Please respond to each point AND highlight all changes.

[Note: HTML markup is below. Please do not edit.]

Author response: All revisions in the manuscript and Supporting Information file have been indicated with Track Changes, plus Comments reporting the specific Reviewer and their respective comment number below.

Reviewers' comments:

Reviewer's Responses to Questions

Comments to the Author

1. Is the manuscript technically sound, and do the data support the conclusions?

The manuscript must describe a technically sound piece of scientific research with data that supports the conclusions. Experiments must have been conducted rigorously, with appropriate controls, replication, and sample sizes. The conclusions must be drawn appropriately based on the data presented.

Reviewer #1: Yes

Reviewer #2: Partly

---

2. Has the statistical analysis been performed appropriately and rigorously?

Reviewer #1: Yes

Reviewer #2: Yes

---

3. Have the authors made all data underlying the findings in their manuscript fully available?

The PLOS Data policy requires authors to make all data underlying the findings described in their manuscript fully available without restriction, with rare exception (please refer to the Data Availability Statement in the manuscript PDF file). The data should be provided as part of the manuscript or its supporting information, or deposited to a public repository. For example, in addition to summary statistics, the data points behind means, medians and variance measures should be available. If there are restrictions on publicly sharing data—e.g. participant privacy or use of data from a third party—those must be specified.

Reviewer #1: Yes

Reviewer #2: Yes

---

4. Is the manuscript presented in an intelligible fashion and written in standard English?

PLOS ONE does not copyedit accepted manuscripts, so the language in submitted articles must be clear, correct, and unambiguous. Any typographical or grammatical errors should be corrected at revision, so please note any specific errors here.

Reviewer #1: Yes

Reviewer #2: Yes

---

5. Review Comments to the Author

Please use the space provided to explain your answers to the questions above. You may also include additional comments for the author, including concerns about dual publication, research ethics, or publication ethics. (Please upload your review as an attachment if it exceeds 20,000 characters)

Reviewer #1: This is an interesting study. The following issues should be addressed.

Author response: We thank Reviewer 1 for their time in reading our manuscript, and providing feedback to improve the report of our findings.

1. Fig. 2. Adjust the Y axis to reflect the low freezing levels seen. Also, it would be better to show these data as bar graphs so that group differences can be appreciated.

Author response: We appreciate that the low freezing levels in the no-shock groups are hard to see. Our goal in keeping the Y axes consistent (0-100% Freezing) across all fear behavior graphs is to emphasize how low the freezing levels by the no shock groups are, relative to shock groups. Likewise, we show individual data points for the no shock groups as we do for the shock groups, so that readers can observe and compare for themselves the individual data points.

Nonetheless, we see Reviewer 1's points, and have thus added S2 Figure to our Supporting Information so that they and future readers can see the data with a smaller Y axis (0-40% Freezing). We also made the testing data (panel B) into bar graphs along with aligned individual data points.

Further, all no shock graphs have also be revised at the request of Reviewer 2 (Comment 5), such that male and female sex symbols are no longer used. Thus, we've assigned triangle symbols to females, and diamond symbols to males.

S2 Figure is pasted below.

S2 Figure

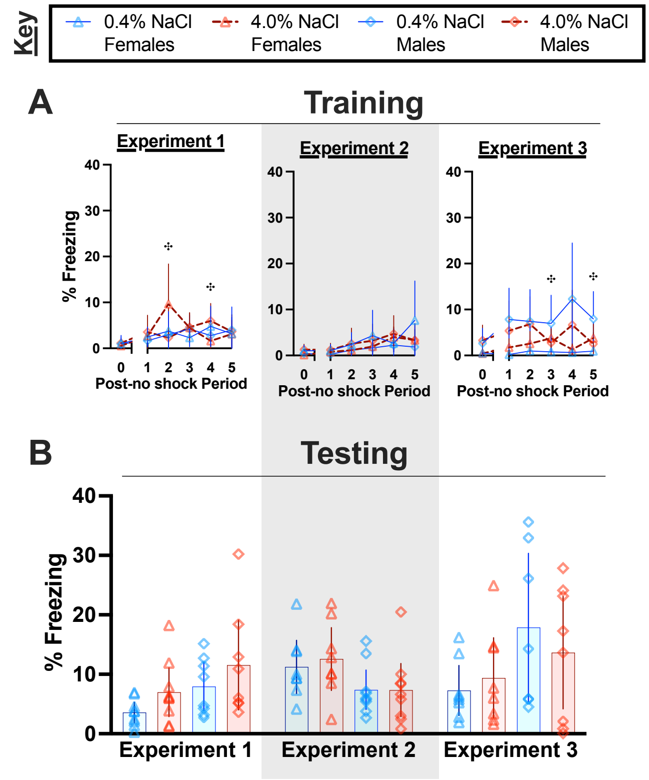

2. Only 5 seconds post the shock are analyzed. It seems better to analyze the largest ISI possible.

Author response: We suspect there is some misunderstanding here. Our manuscript states (page 7),

"*Freezing behavior was quantified as the average percent freezing across the first six 5 s bins (i.e., 30 s) that occurred immediately after the 5 s bin containing the foot shock.*"

In other words, data from context fear training are collected in 5 s bins. When the 1 s shock occurs, that is contained within a 5 s bin. The six 5 s bins immediately following the single 5 s bin containing the shock are analyzed as a 30 s block, with the average freezing across those six bins (each 5 s) being analyzed as a post-shock period. The exact equivalent time in the training procedure was also analyzed and graphed for no shock groups. In sum, we are analyzing 30 s post-shock, not 5 s post shock. We hope this resolves Reviewer 1's concern.

3. Females and males respond different to singly housing. Therefore, it is conceivable that in experiment 3 sex-dependent effects of singly housing might have contributed to the sex-dependent effects seen. This should be acknowledged.

Author response: We appreciate Reviewer 1's point, and have added text to our discussion of the limitations of our study. The revised text (page 23) is copied below.

> "*Limitations of this study include singly housing of mice to allow for tracking of individual food/water/salt/kcal consumption and changes in body weight,*

meaning that mice were excluded from social stimulation. Some data suggests behavioral responses to single housing differs across the sexes in mice and rats (see review [66]), so this might have influenced sex-specific behaviors observed in the present study. However, a meta-analysis of 293 male and female mouse studies indicates that singly housing mice reduces coefficients of variation in trait variability by 37% in both sexes [67], suggesting our housing condition may have reduced the variability of our data."

4. It seems better to move figure panels not showing any significance from the main figure to supplementary figures. Some figure, like figure 6, could be a main or supplementary table as well. Fig. 4 is the main figure and receives less attention now.

Author response: We understand the tendency to focus on significant findings, and to push non-significant (i.e., negative) findings away. One of the primary reasons we initially chose to submit our manuscript to PLOS ONE was their statement acknowledging the importance of sharing negative findings:

"*We evaluate research on the basis of scientific validity, strong methodology, and high ethical standards—not perceived significance. Multidisciplinary and interdisciplinary research, replication studies, negative and null results are all in scope.*"

Source: https://journals.plos.org/plosone/s/journal-information#loc-criteria-for-publication
Accessed 10 Apr 2023.

In line with this, we think it is important to keep some of our negative findings within the main manuscript. Moreover, we think it is the absence of changes in physiological indicators of stress (serum osmolality & corticosterone) that make our behavioral findings all the more interesting. We state the same in the last sentence of the first paragraph in our discussion. Consequently, while we have moved graphs and analyses for the no shock control group into the Supporting Information (S1, S2, S6, S11, S13 Tables; S1-S3, S5-S7 Figs.) at the advice of Reviewer 1, we respectfully prefer to keep the negative data graphs (Figs. 4-5) for the experimental shock groups in the main portion of the manuscript. In particular, we think showing the serum osmolality and corticosterone data for the shock groups are critical, given they provide context for interpreting our significant behavioral findings. Further, this conveys that negative findings can still be important findings, in line with the scope of PLOS ONE.

5. There seems more of a sex difference in the training context in Experiments 1 and 2 in Fig. 4 than in Experiment 3. One wonder whether this might have contributed to what is seen in Experiment 3. In females, the pattern in the neutral context seems definitely similar in Experiments 2 and 3.

Author response: We thank Reviewer 1 for their close attention to our manuscript, and their careful thoughtfulness about our findings.

Based on our statistical analyses of the data presented in what is now Figure 3 (now reported in S7 Table), the effect size for the significant Sex × Context interaction for Experiment 3 is partial η^2=**0.142**, whereas for this same interaction in Experiment 2 the effect size is partial η^2=**0.076**. There is not a significant Sex × Context interaction for Experiment 1, but the significant main effect of sex for Experiment 1 has an effect size of partial η^2=**0.117**.

Taken together, we interpret this to indicate that the contribution of sex to these outcomes is strongest in Experiment 3, and weakest in Experiment 2, with Experiment 1 landing in the middle. Of course, for the first two, sex must be considered with context in mind. Because there is no significant Sex × Context interaction for Experiment 1, we conclude that there is no sex difference dependent upon context for Experiment 1. Thus, it does not appear that what is happening in Experiment 1 would necessarily contribute to what is happening in Experiment 3. Moreover, to address Reviewer 1's point about the similarity of behavioral patterns in females tested in the neutral context in Experiments 2 and 3, we would need to do some four-way ANOVAs or other more advanced statistics. Based on Reviewer 1's Comment 9, we take this to be undesirable. Visually, we agree with Reviewer 1 that these do look to have similar patterns. But overall, we think that the differences observed in Experiment 3, relative to Experiments 1 and 2, are likely largely attributable to the time gap (4 wks) necessary to elicit fear generalization and the ability of the sex of the animal to interact with fear generalization processes during that lengthy period.

6. The salt effect in females in the neutral context seems subtle and not different between the two salt diet groups. The salt effect in males in the neutral context seems more pronounced.

Author response: We again want to express our gratitude for Reviewer 1 time investment in thinking about our data. We think Reviewer 1 is referring to panel C of what is now Figure 3 for this comment, so our response is operating upon this premise.

We agree that the female behavior in the neutral context for Experiment 3 is not statistically different. We think an assessment of "subtle" is a matter of personal interpretation, and the right of every reader to decide. Statistically speaking, females on the low salt diet and tested in the neutral context exhibited generalized fear behavior that was statistically lower than fear behavior by low salt diet females tested in the training context. In contrast, females on the high salt diet and tested in the neutral context exhibited generalized fear behavior that was statistically indistinguishable from fear behavior by high salt diet females tested in the training context. This difference is what we focused on for the females, as well as the directionally opposite change observed for males. Males on the high salt diet and tested in the neutral context did have significantly lower freezing, both compared to males on the high salt diet tested in the training context, and compared to females on the high salt diet tested in the neutral context.

In terms of the effect of salt diet consumption being "more pronounced" in males than females, again this is a descriptor that we leave to readers to conclude. In evaluating the overlap of the 95% confidence intervals across diet conditions within each sex tested in the neutral context, the sexes visually appear equivalent – it is only the directionality of high salt vs. low salt comparisons that differ across the sexes. This is backed up by our statistical analyses, as within each sex in panel C, there is not a post-hoc difference across any salt treatment. Rather, there are significant interactions between Sex × Context and Sex × Diet in Experiment 3 that lead to these exciting findings.

7. It would be better to show the real cort data so that others can compare with other designs.

Author response: We recognize Reviewer 1's desire to see the serum corticosterone data prior to it being log-transformed to enable normalized distributions for statistical analyses. Consequently, we have added S7 and S8 Figures two figures to our Supporting Information containing the raw serum corticosterone values, graphed for both shock and no shock groups.

Importantly, these data were not statistically analyzed because of their non-normal distribution, hence why we log-transformed the data prior to statistical analyses.

S7 and S8 Figures with these raw serum corticosterone data are also copied below.

S7 Figure

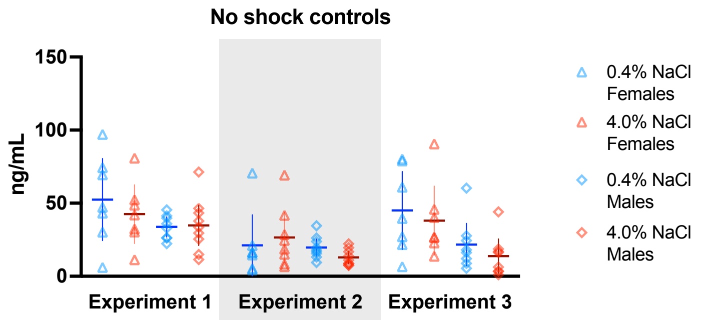

S8 Figure

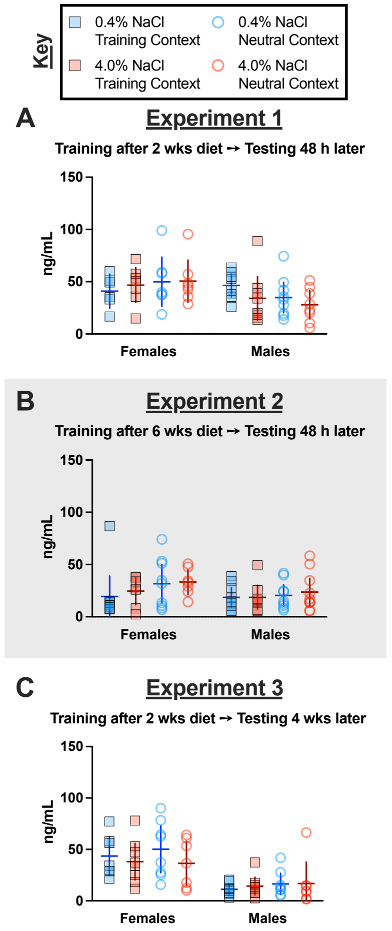

8. It would be better to remove p values from the figures, especially if they do not reach significance.

Author response: We have removed the three instances where p values were present on graphs.

9. The text in the results section is written very stats heavy. While all correct, this makes it harder to read for those less versed in statistical interactions. The authors are encouraged to rephrase some of the text to make this manuscript more readable. The tables with the stats data are good and allow focusing less on stats result in the text.

Author response: We thank Reviewer 1 for sharing this perspective. As a consequence, we have largely overhauled the writing in the Results section, removing statistical jargon when possible, and making the text more accessible and understandable in the context of the broader research question. Given the large size of this content, we have not pasted it below. The revised Results section is found on pages 11-19 of the revised manuscript.

Reviewer #2: This manuscript evaluates that possibility that a forced, high-salt diet affects contextual fear acquisition, recall and generalization in male and female C57BL/6J mice. I think there is some important data here, and it should be seen. Most of my concerns are with how the data, statistics, etc. are presented. Given that this is a straight-forward experiment, it would benefit to attempt to streamline what is being presented to the reader. I found myself paying too much attention to small details the were being presented/suggested rather than being able to focus on the main message (or two) as I read through the Results and Discussion presentation.

Author response: We thank Reviewer 2 for taking the time to review our manuscript, and for their comments to make communication of our study's findings better.

Please note – we have added numbers for the specific comments below, to better direct the Reviewers and Editors to where in the text revisions were made to address each of Reviewer 2's comments.

Specific comments:

1. Introduction: Lines 68-70 – I think it would be useful to provide some specific detail on how hippocampus/amygdala are affected by high-salt.

Author response: We agree with Reviewer 2, and have added text to outline specific changes observed in these two brain regions following consumption of high salt. The expanded text (pages 3-4) is copied below.

"*Growing evidence indicates the hippocampus and amygdala are affected by excess salt consumption in rodents [24,26,35–41], including: reductions in nicotinic receptor levels [35]; increased markers of oxidative stress [24,26,37,41], neuronal activation [42], and neuroinflammation [42]; reduced long-term potentiation [40]; and increased microvasculature leakiness [36]. Other brain regions remain underexplored.*"

2. Consider providing a clear "N per group/sex" in the Methods.

Author response: We have added text to the Statistical Analyses subsection of the Methods that states the numbers of mice for each set of analyses/graphs covered in the manuscript. Because the amount varies for each combination, we provide ranges of numbers, and refer readers to the Figure legends containing specific numbers for each individual group. The revised text (page 9) is copied below.

"*Numbers of mice per sex/shock/diet/context condition varied, and exact numbers are present in the respective figure legends for each group of data. Broadly: no shock fear behavior n=7-9; shock fear behavior n=7-10; shock serum osmolality n=8-9; shock serum corticosterone n=7-9.*"

3. Describe in more detail why 5x shock was used. 1 or two is sufficient to engender contextual fear.

Author response: We have added a sentence in our Context Fear Conditioning subsection of the Methods to explain why we used five shocks – this is standard for facilitation of contextual fear generalization over time. To compare Experiment 3 mice to mice in Experiments 1 and 2, we kept the shock numbers the same. The added text (page 7) is copied below.

"*Training with five foot shocks is optimal for eliciting time-dependent contextual fear generalization in rodents [44–46].*"

4. Page 8, Line 158 – Much more thorough justification needs to be provided for why the authors are selectively presenting/analyzing data from minutes 2-6 out of the 10-minute test. Without this, it appears that the data has been "cherry picked" to show some effects.

Author response: We assure Reviewer 2 that we have not "cherry picked" our data, and never expected to convey that impression given how transparent we have been with all of our data and analyses.

This five minute time frame is a standard one we use when analyzing context fear behavior. It is best explained in the words of one of our previous publications: "Testing consisted of a 10 min exposure to the training or neutral context, during which freezing was measured during minutes 2 through 6 in order to allow for contextual recognition, acclimation to the chamber and to avoid any extinction effects" Lynch et al., 2017 *Neuropsychopharmacol* 42(4):914–24.

In other words, our goals is to capture context fear expression during a five minute period that is not confounded by the mouse initially orienting themselves to the environment (minute 1) nor by extinction learning that becomes apparent during the second half of the testing session, given

the mouse has been present in the context and nothing aversive (e.g., foot shock) has occurred (minutes 6-10).

To assure Reviewer 2, Reviewer 1, and the Editors, we have added graphs (S3 and S4 Figs.) and analyses (S6 and S8-S10 Tables) of the time courses of context fear expression in the Supporting Information. Given the large size of this content, we have not pasted it below.

5. Figure 2 – It is confusing to refer to a “post-shock period” when these experiments did not employ shock. Also, I find using the sex-symbols as data points to be distracting. Could you more simply use blue/red squares and circles?

Author response: We thank Reviewer 2 for pointing out the confusion. Our intention was to convey that we were extracting the exact same time points during ‘training’ for graphing of no shock data to compare with those of shock data. We have now revised the X-axis labels, of what is now S1 Fig., to read “Post-no shock Period” to make it clear that no shock animals did not receive a shock.

Similarly, our rationale for using female and male sex symbols was to differentiate that these graphs contained data from no shock groups, whereas data presented for shock groups used squares and circles. Consequently, we have removed the sex symbols for all control no shock graphs, and instead assigned triangle symbols to females, and diamond symbols to males (S1-S3, S5-S7, S13, S14, S20-S22, S24 Figs.).

6. Consider placing the tables in a data supplement. There are 10 of them, several with multiple parts, and they are essentially all ANOVA tables.

Author response: We sympathize with Reviewer 2’s sentiments regarding the tables and all the statistics they contain as being overwhelming. We thank Reviewer 2 for this recommendation, which we have followed.

7. Page 26, Line 526 – The overall statistical analyses appear to be rigorous. The notion of a “trend” presented in the Discussion detracts from this.

Author response: We have omitted mention of a trend in the discussion. The edited sentence (page 20), which previously mentioned the trend is copied below.

> “*Their observations differ from ours, which did not observe significant effects of high salt diet on contextual fear expression in the Training Context.*”

8. Page 27, Line 548 – The above is also true for the notion of a “bimodal” distribution, which would be very difficult to show with that number of data points.

Author response: We removed the entire sentence that mentioned the apparent bimodally distributed data, as well as the subsequent sentence that referred back to the bimodal distribution (page 21).

9. Page 28, Line 579 – Same for “non-significant trends”

Author response: We removed the portion of this sentence that referred to "non-significant trends. The edited sentence (page 22) appears below.

> "*No significant correlations between cort levels and fear expression in the Neutral Context were detected for either sex in Experiment 3.*"

10. How do the authors know that 0.8mA is not painful?

Author response: We take Reviewer 2's point that we cannot know the inner experience of the mice. This statement was based on our own experiences, as in our lab we always manually feel the shock to be administered first before training any mice on a given experimental day. Nonetheless, we appreciate that the absence of pain for us does not mean absence of pain for the mice, and thus we have removed reference to pain in this sentence. The revised sentence (page 22) appears below.

> "*Though the shock level (0.8 mA) and duration (1 sec) that we use are intended to only elicit discomfort/distress, it remains possible that osmotic stress could skew sensory perception of this brief aversive experience*."

11. While I think the authors are likely correct about the lack of social simulation and light-phase testing in their experiment, bringing this up distracts from the overall message regarding the data at hand. For example, it begs the question why reverse-phase lighting was not used in the first place?

Author response: We simultaneously agree with Reviewer 2 that mention of such considerations can distract from the main findings, but also think that acknowledging limitations of every study is important for the scientific method. To try to reconcile these two important perspectives, we have added a sub-heading for the referenced paragraph to make it clear that the content therein outlines <u>limitations</u> specifically. Additionally, we have removed the sentence referring to light:dark cycle, but retained the housing statement given Reviewer 1's Comment 3 so that we address their concern as well.

So that the Reviewers and Editors are aware, we personally would much prefer to use reverse-phase lighting in our lab, but unfortunately our Animal Facility Manager has taken an adamant stance against it for personal reasons. Once I am no longer probationary faculty and have significant grant funding in hand, this is definitely an issue I will be revisiting with administration at my institution.

The revised and newly labeled Limitations sub-section of the Discussion is now located on page 23 of the revised manuscript.

12. Page 28, Line 566 – Sentence beginning "Nonetheless…". I would like the authors to expand more upon this. From a biological standpoint, I'm left hanging and wondering "how would this be possible?". Perhaps the authors can draw on other work for examples outside of hypertension/anxiety.

Author response: We thank Reviewer 2 for the opportunity to expand upon our postulation by providing some literature support. Our original sentence, referenced above, plus added supporting sentences (page 22), are included below.

"*Nonetheless, it may be that in males, high salt-mediated cardiovascular pathology is necessary for emergence of increased anxiety-related behaviors, whereas in females excess salt is sufficient. This would parallel findings indicating greater metabolic disruptions in men relative to women with comorbid obesity and mental health disorders [57–59] (see review [60]), greater incidence of depression and anxiety in men versus women with multiple sclerosis [61], and increased incidence of a biomarker of cardiovascular disease in men versus women with depression [62]. However, some literature suggests this is not a consistent finding across disease conditions [63].*"

13. Do the authors think their findings would generalize to more classic mouse tests of "anxiety", such as EPM or open field?

Author response: We understand Reviewer 2's question, and have added a sentence and modified the subsequent sentence in our Introduction to address this. The majority of the literature (Ernsberger et al., 1983 Guo et al., 2017; Ge et al., 2017; Gilman et al., 2019) indicates that high salt intake does not affect characteristics of trait-like anxiety that tests like the elevated plus maze and open field query. One study did find some indications of enhanced trait-like anxiety after high salt intake, but only in aged male rats, not in young male rats (Chugh et al., 2013). In contrast, these effects of excess salt consumption appear to be selective for acquired, or state, anxiety-like behaviors (i.e., fear generalization). The revisions (page 3) in the Introduction are included below.

"*Trait anxiety appears largely unaffected by excess salt consumption [23–27] (reviewed in [28]). Far less understood is how excess salt intake might affect neuropsychiatric symptoms like generalized (i.e., non-specific, state) anxiety, though some researchers have begun to recognize this possibility [29,30].*"

---

6. PLOS authors have the option to publish the peer review history of their article (what does this mean?). If published, this will include your full peer review and any attached files.

If you choose "no", your identity will remain anonymous but your review may still be made public.

Do you want your identity to be public for this peer review? For information about this choice, including consent withdrawal, please see our Privacy Policy.

Reviewer #1: No

Reviewer #2: No
